# A Data Set and Deep Learning Algorithm for the Detection of Masses and Architectural Distortions in Digital Breast Tomosynthesis Images

**DOI:** 10.1001/jamanetworkopen.2021.19100

**Published:** 2021-08-16

**Authors:** Mateusz Buda, Ashirbani Saha, Ruth Walsh, Sujata Ghate, Nianyi Li, Albert Święcicki, Joseph Y. Lo, Maciej A. Mazurowski

**Affiliations:** 1 Department of Radiology, Duke University Medical Center, Durham, North Carolina; 2 Department of Electrical and Computer Engineering, Duke University, Durham, North Carolina; 3 Department of Computer Science, Duke University, Durham, North Carolina; 4 Department of Biostatistics and Bioinformatics, Duke University Medical Center, Durham, North Carolina

## Abstract

**Importance:**

Breast cancer screening is among the most common radiological tasks, with more than 39 million examinations performed each year. While it has been among the most studied medical imaging applications of artificial intelligence, the development and evaluation of algorithms are hindered by the lack of well-annotated, large-scale publicly available data sets.

**Objectives:**

To curate, annotate, and make publicly available a large-scale data set of digital breast tomosynthesis (DBT) images to facilitate the development and evaluation of artificial intelligence algorithms for breast cancer screening; to develop a baseline deep learning model for breast cancer detection; and to test this model using the data set to serve as a baseline for future research.

**Design, Setting, and Participants:**

In this diagnostic study, 16 802 DBT examinations with at least 1 reconstruction view available, performed between August 26, 2014, and January 29, 2018, were obtained from Duke Health System and analyzed. From the initial cohort, examinations were divided into 4 groups and split into training and test sets for the development and evaluation of a deep learning model. Images with foreign objects or spot compression views were excluded. Data analysis was conducted from January 2018 to October 2020.

**Exposures:**

Screening DBT.

**Main Outcomes and Measures:**

The detection algorithm was evaluated with breast-based free-response receiver operating characteristic curve and sensitivity at 2 false positives per volume.

**Results:**

The curated data set contained 22 032 reconstructed DBT volumes that belonged to 5610 studies from 5060 patients with a mean (SD) age of 55 (11) years and 5059 (100.0%) women. This included 4 groups of studies: (1) 5129 (91.4%) normal studies; (2) 280 (5.0%) actionable studies, for which where additional imaging was needed but no biopsy was performed; (3) 112 (2.0%) benign biopsied studies; and (4) 89 studies (1.6%) with cancer. Our data set included masses and architectural distortions that were annotated by 2 experienced radiologists. Our deep learning model reached breast-based sensitivity of 65% (39 of 60; 95% CI, 56%-74%) at 2 false positives per DBT volume on a test set of 460 examinations from 418 patients.

**Conclusions and Relevance:**

The large, diverse, and curated data set presented in this study could facilitate the development and evaluation of artificial intelligence algorithms for breast cancer screening by providing data for training as well as a common set of cases for model validation. The performance of the model developed in this study showed that the task remains challenging; its performance could serve as a baseline for future model development.

## Key Points

**Question:**

Can a curated, annotated, and publicly available data set of digital breast tomosynthesis (DBT) volumes be created for the development and validation of breast cancer computer-aided detection algorithms?

**Findings:**

In this diagnostic study, a curated and annotated data set of DBT studies that contained 22 032 reconstructed DBT volumes from 5060 patients was made publicly available. A deep learning algorithm for breast cancer detection was developed and tested, with a sensitivity of 65% on a test set.

**Meaning:**

In this study, the publicly available data set, alongside the deep learning model, could significantly advance the research on machine learning tools in breast cancer screening and medical imaging in general.

## Teaser

This diagnostic study presents a large-scale data set of digital breast tomosynthesis images to facilitate the development and evaluation of artificial intelligence algorithms for breast cancer screening and develops and tests a baseline deep learning model for breast cancer detection.

## Introduction

Deep learning emerged mainly due to rapid increases in access to computational resources and large-scale labeled data.^1^ Medical imaging is a natural application of deep learning algorithms.^2^ However, well-curated data are scarce, which poses a challenge in training and validating deep learning models. Annotated medical data are limited for a number of reasons. First, the number of available medical images is much lower than the number of available natural images. This is particularly an issue when investigating a condition with fairly low prevalence, such as breast cancer in a screening setting (<1% of screening examinations result in a cancer diagnosis). Second, access to medical imaging data is guided by a number of strict policies given that they contain patients’ medical information. Sharing of medical imaging data requires an often nontrivial and time-consuming effort to deidentify the data as well as ensure compliance with requirements from the institution that is sharing the data and beyond. Finally, annotation of medical imaging data typically requires the work of radiologists, who already have high demands on their time.

As a result, the amount of well-annotated large-scale medical imaging data that are publicly available is limited. This is certainly a problem when training deep learning models, but it also results in a lack of transparency when evaluating model performance.

Limited reproducibility of results has been particularly visible in mammography research, arguably the most common radiology application of artificial intelligence (AI) in the last 2 decades.^3,4,5,6^ Researchers use different, often not publicly available, data sets and solve related but different tasks.^7^ Moreover, studies have different evaluation strategies, which makes it difficult to reliably compare methods and results. An AI system must be extensively validated before application in clinical practice. A common shortcoming in many studies is that the test set was obtained from a single institution and a limited number of devices.^8^ In addition, some studies make exclusions from the data, which further obscure the true performance of the algorithms.

In this study, we aimed to address some of these challenges. First, we curated and annotated a data set of more than 22 000 three-dimensional (3D) digital breast tomosynthesis (DBT) volumes from 5060 patients. DBT is a new modality for breast cancer screening that, instead of projection images (as in mammography), delivers multiple cross-sectional slices for each breast and offers better performance.^9^ We are making this data set publicly available at the Cancer Imaging Archive,^10^ a public data hosting service for medical images of various modalities together with community analyses that facilitate the usability of shared data sets. This will allow other groups to improve the training of their algorithms as well as test their algorithms on the same data set, which could improve both the quality of the models and comparison between different algorithms. This could also allow groups that have access to strong machine learning expertise but no clinical data to contribute to the development of clinically useful algorithms.

In addition, we developed and made publicly available a single-phase deep learning model for the detection of abnormal results in DBT that can serve as a baseline for future development or be used for fine-tuning in solving other medical imaging tasks. To our knowledge, this is the first published single-phase deep learning model for DBT. Given that the major challenge of developing the model for this task is a very limited number of positive locations, we evaluated and compared different methods for addressing this issue.

## Methods

### Data Set

**Figure 1.**
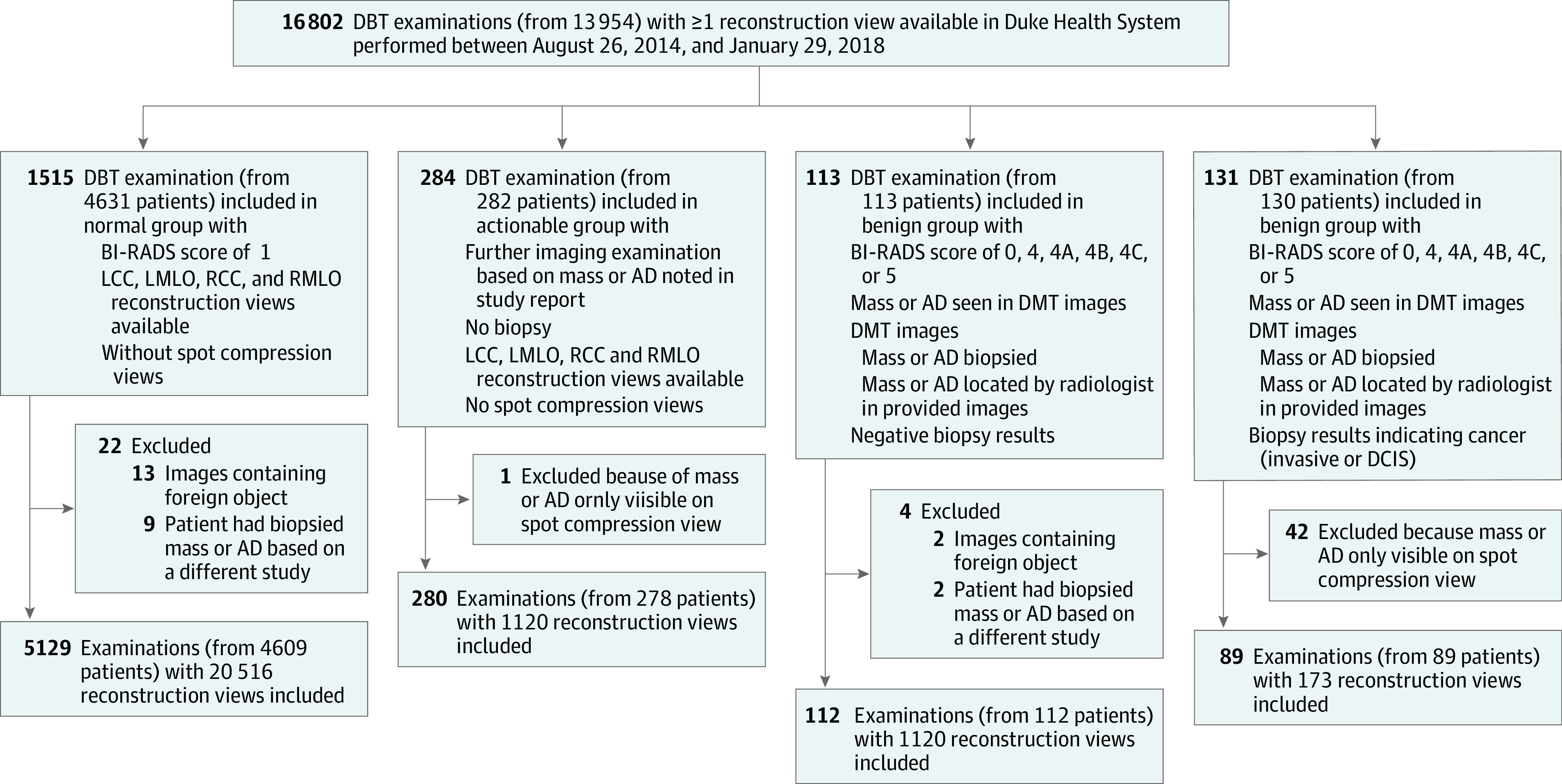
Patient Flowchart AD indicates architectural distortion; BI-RADS, Breast Imaging-Reporting and Data System; DBT, digital breast tomosynthesis; LCC, left craniocaudal; LMLO, left mediolateral oblique; RCC, right craniocaudal; RMLO, right mediolateral oblique.

This study was approved by the Duke University Health System institutional review board with a waiver of informed consent due its retrospective nature. We analyzed DBT volumes obtained from Duke Health System, following the Standards for Reporting of Diagnostic Accuracy (STARD) reporting guideline. Specifically, Duke Health Systems Duke Enterprise Data Unified Content Explorer tool was queried to obtain all radiology reports having the word *tomosynthesis* and all pathology reports having the word *breast* within the search dates of January 1, 2014, to January 30, 2018. The image download based on the study dates and medical record numbers obtained from the radiology reports resulted in an initial collection of 16 802 DBT studies from 13 954 patients performed between August 26, 2014, and January 29, 2018, with at least 1 of the 4 reconstruction volumes (ie, left craniocaudal [LCC], right craniocaudal [RCC], left mediolateral oblique [LMLO], and right mediolateral oblique [RMLO]) available. From this cohort, we divided the studies into 4 groups, as shown in the patient flowchart (Figure 1) and described below.

The normal group included 5129 screening studies from 4609 patients without any abnormal findings that were not subject to further imaging or pathology examinations related to the study in question. Specifically, in this group we included studies that had a Breast Imaging-Reporting and Data System (BI-RADS) score of 1; had LCC, LMLO, RCC, and RMLO reconstruction views available; did not use the words *mass* or *distortion* in the corresponding radiology report, and did not contain spot compression among the 4 views. Spot compression was established based on text processing of radiology reports combined with visual inspection of images. Studies with images containing foreign objects other than implants and markers (n = 13) and studies from patients who had biopsied mass or architectural distortion based on a different DBT study (n = 9) were excluded.

The actionable group included 280 studies from 278 patients that resulted in further imaging examination based on a mass or architectural distortion noted in the study report. Specifically, we included studies that had a recommendation for a further imaging examination based on a mass or architectural distortion noted in the study report; did not result in a biopsy; had LCC, LMLO, RCC, and RMLO reconstruction views available; and did not contain spot compression among the 4 views. Spot compression was established in the same manner as in the normal group. Studies with images containing foreign objects other than implants and markers (n = 2) and studies from patients that had biopsied mass or architectural distortion based on a different DBT study (n = 2) were excluded.

The benign group included 112 studies from 112 patients containing benign masses or architectural distortions biopsied based on this DBT examination. Specifically, in this group we included studies that had a BI-RADS score of 0, 4, 4A, 4B, 4C, or 5; had a mass or architectural distortion that was seen in the DBT imaging study in question that was identified using laterality and/or location noted in a related breast pathology report and was biopsied; had benign results of all biopsies per the pathology reports; and a radiologist was able to retrospectively locate at least 1 of the biopsied benign masses or architectural distortions in the reconstruction views from the study. One study for which the biopsied mass was visible only on spot compression views was excluded.

The cancer group included 89 studies from 89 patients with at least 1 cancerous mass or architectural distortion that was biopsied based on this DBT examination. Specifically, we included studies that had a mass or architectural distortion seen in the DBT images that was identified using laterality and/or location noted in a related breast pathology report and was biopsied; had at least 1 biopsied mass or architectural distortion corresponding to cancer (invasive or ductal carcinoma in situ) per the pathology report; and a radiologist was able to retrospectively locate at least 1 of the biopsied cancerous mass or architectural distortion in the reconstruction views from the study. Studies for which all cancerous masses or architectural distortions were visible only on spot compression views (n = 42) were excluded. More details on the exclusion of cases from the initial population are provided in eAppendix 1 in the Supplement.

### Training, Validation, and Test Sets

In total, our data set contained 22 032 reconstructed volumes that belonged to 5610 studies from 5060 patients. It was randomly split into training, validation, and test sets in a way that ensured no overlap of patients between the subsets. The test set included 460 studies from 418 patients. For the validation set, we selected 312 studies from 280 patients, and the remaining 4838 studies from 4362 patients were in the training set. The selection of cases from the benign and cancer groups into the test and validation sets was performed to assure a similar proportion of masses and architectural distortions. Descriptive statistics for all the subsets are provided in Table 1.

**Table 1.** Descriptive Statistics of the Data Set Used for Training, Validation, and Testing

Characteristics	No.		
	Training set	Validation set	Test set
Patients			
Total	4362	280	418
Normal group, No. (%)	4109 (94.2)	200 (71.4)	300 (71.8)
Actionable group, No. (%)	178 (4.1)	40 (14.2)	60 (18.9)
Benign group, No. (%)	62 (1.4)	20 (7.1)	30 (7.2)
Cancer group, No. (%)	39 (0.9)	20 (7.1)	30 (7.2)
Studies	4838	312	460
Reconstruction volumes	19 148	1163	1721
Bounding boxes for cancerous lesions	87	37	66
Bounding boxes for benign lesions	137	38	70
Bounding box diagonal, mean (SD), pixels	344 (195)	307 (157)	317 (166)

### Image Annotation

Study images along with the corresponding radiology and pathology reports for each biopsied case were shown to 2 radiologists at our institution (R.W. and S.G.) for annotation. We asked the radiologists to identify masses and architectural distortions that were biopsied and to put a rectangular box enclosing them in the central slice using a custom software developed by a researcher (N.L.) in our laboratory. Each case was annotated by 1 of 2 experienced radiologists. The first radiologist, with 25 years of experience in breast imaging (R.W.), annotated 124 cases, whereas the second radiologist, with 18 years of experience in breast imaging (S.G.), annotated 77 cases. This way we obtained 190 bounding boxes for cancerous lesions in 173 reconstruction views and 245 bounding boxes for benign lesions in 223 reconstruction views. There were 336 and 99 bounding boxes for masses and architectural distortions, respectively, across cancerous and benign lesions.

### Baseline Algorithm

#### Preprocessing

First, we applied a basic preprocessing by window leveling images based on information from the Digital Imaging and Communications in Medicine file header. Then, each slice was downscaled by a factor of 2 using 2 × 2 local mean filter to reduce computational and memory footprint. After that, we eroded nonzero image pixels with a filter of 5-pixel radius for skin removal. Finally, we extracted the largest connected component of nonzero pixels for segmenting the breast region.

#### Detection Algorithm

For a baseline method to detect lesions, we used a single-phase fully convolutional neural network for 2-D object detection^11^ with DenseNet^12^ architecture. The model processes each 2-D input slice independently. Following this,^11^ raw model predictions correspond to a grid in the input slice image with cells sized 96 × 96 pixels. For each cell, the network outputs a confidence score for containing the center point of a box and 4 values defining the location and dimensions of the predicted box. A bounding box is defined by offset from the cell center point as well as scale in relation to a square anchor box sized 256 × 256 pixels.^13^ Each cell was restricted to predicting exactly 1 bounding box.

The network was optimized using Adam,^14^ with an initial learning rate of 0.001 and batch size of 16 for 100 epochs over positive examples and early stopping strategy with a patience of 25 epochs. Weights were randomly initialized using the Kaiming method,^15^ and biases in the last layer were set according to Lin et al.^16^

For training, we sampled positive slices containing ground truth boxes from volumes belonging to the biopsied groups. The number of positive slices (ie, slices containing a tumor) was established as the square root of the average dimension in pixels of the box drawn by a radiologist on the center slice of the tumor. The ground truth 3-D box was defined by the 2-D rectangle drawn by the radiologist with the third dimension defined by the number of slices, as described previously. Then, we randomly cropped a slice image to a size of 1056 × 672 pixels, which resulted in an output grid sized 11 × 7 pixels so that the cropped slice image included the entire ground truth bounding box. For validation, the slice span of ground truth boxes was reduced by a factor of 2 compared with the training phase, and we fixed selected slice and cropped slice image regions for each case. This was done to ensure comparable validation performance was measured based on the same input slice for all runs and across epochs. All hyperparameters and algorithmic strategies described previously were decided on the validation set.

During inference, we used entire image slices as the input and padded them with zeros when necessary to match the label grid size. To obtain predictions for a volume, we split it into halves and combined slice-based predictions for each half by averaging them. Then, we applied the following postprocessing. First, predicted boxes for which fewer than half the pixels were in the breast region were discarded to eliminate false-positive predictions outside of the breast. Then, we applied a nonmaximum suppression algorithm^17^ by merging all pairs of predicted boxes that had a confidence score ratio of less than 10 and an intersection over union greater than 50%. The confidence score of a resulting box was a maximum of scores from the 2 merged boxes.

#### Experiments

To provide an insight into the effects of different hyperparameters on the performance, we performed a grid search over different network sizes and objectness loss functions that address the problem of class imbalance.^18^ Our problem was characterized by a significant imbalance between the bounding boxes corresponding to lesions and background class that the network learns to distinguish in the training process. The 4 tested loss functions for addressing this problem were: (1) binary cross-entropy, (2) weighted binary cross-entropy, (3) focal loss,^16^ and (4) reduced focal loss.^19^ Weighted binary cross-entropy assigns different weights to positive and negative examples based on class prevalence. Focal loss is a parametrized loss function that reduces the importance of examples that are correctly classified without high confidence, as shown in eAppendix 1 in the Supplement. Finally, reduced focal loss is equivalent to binary cross-entropy for examples misclassified with a confidence lower that 0.5, and after this threshold, loss value is gradually reduced to focal loss. For bounding box localization loss, we used mean squared error as in Redmon et al.^11^ In total, we trained 768 models, and the results from all runs are provided in eAppendix 2 in the Supplement. The code for all experiments and network architecture together with the trained model weights are publicly available.^20^

In the grid search, model selection was based on the sensitivity at 2 false positives per slice computed on the validation set after every epoch. For each loss function, we selected the best performing model for 3-D evaluation on the entire validation set. Following this 3-D evaluation, the model with the highest sensitivity at 2 false positives per DBT volume on the validation set was used to generate predictions on the test set for the final evaluation.

#### Final Model Evaluation on the Test Set

For the final evaluation of the baseline detection algorithm, we used the free-response receiver operating characteristic (FROC) curve, which shows the sensitivity of the model in relation to the number of false-positive predictions placed in slice images, volumes, or cases. A predicted box was considered a true positive if the distance between its center point and the center of a ground truth box was either smaller than half of the ground truth box diagonal or smaller than 100 pixels. The additional 100 pixels condition was implemented to prevent punishing correct detections for very small lesions with unclear boundaries. In terms of the third dimension, the ground truth bounding box was assumed to span 25% of volume slices before and after the ground truth center slice, and the predicted box center slice was required to be included in this range to be considered a true positive.

In addition to the volume-based evaluation described above, we evaluated the accuracy of model predictions using breast-based FROC. In this case, a prediction for a breast was considered true positive if any lesion on any view for this breast was detected according to the criteria described above. This metric most accurately reflects the model performance in a clinical setting.

### Statistical Analysis

For the final evaluation of the baseline detection algorithm, we used the FROC curve, which shows the sensitivity of the model in relation to the number of false-positive predictions placed in slice images, volumes, or cases. Sensitivity values are reported together with 95% CIs, which were computed using bootstrapping with 2000 bootstraps. For this, we used an open-source statistical tool implemented in Python.^21^

## Results

The number of patients in the data set was 5060, with 5059 women (100.0%) and 1 man (<0.1%). The mean (SD) age at the date of patient’s first examination included in our data set was 55 (11) years. Age statistics were computed based on 5059 patients. The date of birth for 1 patient was unknown. Table 2 provides demographic characteristics for patients in our data set.

**Table 2.** Characteristics of Patients in the Data set

Characteristic	Participants, No. (%)
Age, mean (SD), y	55 (11)
Missing age	1 (<0.1)
Sex	
Women	5059 (100.0)
Men	1 (<0.1)
Race	
White	3700 (73.1)
Black or African American	957 (18.9)
Asian	180 (3.6)
American Indian or Alaskan Native	11 (0.2)
Native Hawaiian or other Pacific Islander	2 (<0.1)
Other^a^	52 (1.0)
≥2 races	56 (1.1)
Not reported, declined, or unavailable	102 (2.0)

^a^ The other category was present in the original data, and it was not specified what groups were included.

### Performance on the Validation Set

All tested loss functions performed similarly, with the best configuration for each loss achieving greater than 78% sensitivity at 2 false positives per slice. Using the best model from the grid search for each loss function in the 2-D per-slice evaluation, we ran inference and evaluated selected models on the entire validation set using the 3-D per-volume evaluation. The best performance, with 60% sensitivity at 2 false positives per DBT volume, was achieved by the network trained using focal loss. In comparison, sensitivity at the same threshold achieved by binary cross-entropy and weighted binary cross-entropy was 59%, whereas reduced focal loss obtained 58%. The model trained using focal loss was selected for evaluation on the test set. More details on the grid search results and FROC curves on the validation set are provided in eAppendix 1 in the Supplement.

### Performance on the Test Set

**Figure 2.**
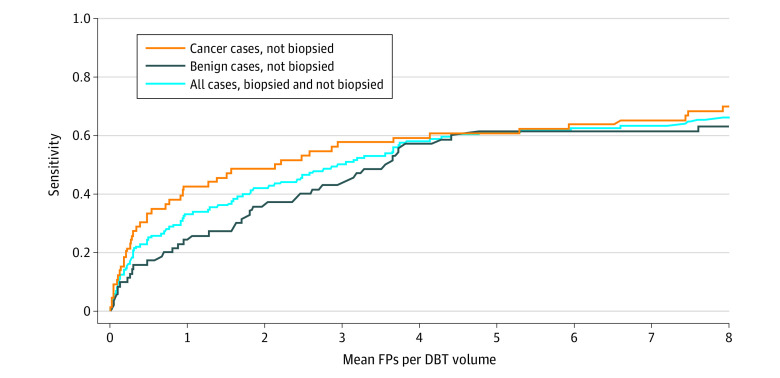
Free-Response Receiver Operating Characteristic Curve Showing Performance on the Test Set of a Model Trained Using Focal Loss DBT indicates digital breast tomosynthesis; FP, false positive.

Using a model trained by optimizing focal loss function, we generated predictions for the test set. The model achieved a sensitivity of 42% (95% CI, 35%-50%) at 2 false positives per DBT volume as shown on the FROC curve in Figure 2. Better performance was reached on the cancer cases than on benign cases.

**Figure 3.**
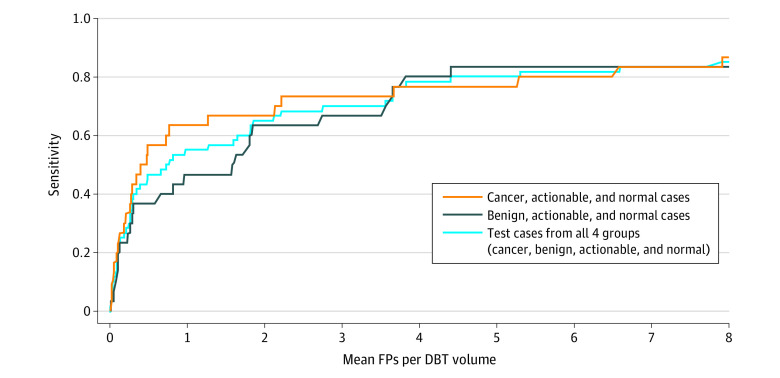
Breast-Based Free-Response Receiver Operating Characteristic Curve for the Test Set DBT indicates digital breast tomosynthesis; FP, false positive.

Finally, we evaluated the selected model using breast-based FROC computed on the test set. In this case, sensitivity at 2 false positives per DBT volume for test cases with cancer and all test cases was 67% (95% CI, 53%-80%) and 65% (95% CI, 56%-74%), respectively. The breast-based FROC curve for the test set is shown in Figure 3.

## Discussion

In this study, we described a large-scale data set of DBT examinations containing data for 5060 patients that we shared publicly. We also trained the first single-phase detection model for this data set that will serve as a baseline for future development.

Our study included annotations for both masses and architectural distortions. Those abnormal findings appear differently in DBT images and therefore constitute a more challenging task for an automated algorithm. A model that focuses on a single task (such as many previously published models for breast imaging) could show overoptimistic performance. This more inclusive data set more accurately represents true clinical practice of breast cancer screening. Furthermore, our data set, which includes normal and actionable cases, is representative of a screening cohort.

Our detection model was developed using only 124 and 175 bounding boxes for cancerous and benign lesions, respectively. No pretraining on other data sets or similar modalities was used. In addition, our detection method is a single-phase deep convolutional neural network, which does not require multiple steps for generating predictions. We showed that a moderate performance can be achieved with a limited training data. In comparison, a previous study^22^ reported sensitivity less than 20% at 2 false positives per volume for a model trained from scratch using only DBT data without pretraining on a much larger data set of mammograms. In another study,^23^ a sensitivity of greater than 80% at 2 false positives per volume was reached for a data set containing only architectural distortions. In Fan et al,^24^ a 3-D deep learning model was developed that achieved 90% sensitivity at 0.8 false positives per volume on a data set containing only abnormal images with masses.

The methods for evaluating performance of detection algorithms vary. The method used in this study is robust to models predicting large bounding boxes as opposed to evaluation methods that consider a predicted box as a true positive if it contains the center point of the ground truth box. In our study, the center point of the predicted box was required to be contained in the ground truth box as well. Furthermore, we were solving a 3-D detection task, which generates a higher number of false positives than 2-D detection tasks. While the performance of our model is not comparable with the performance of radiologists, our goal was to set a baseline for a model that is trained only on the provided data and without access to large-scale computer clusters.

### Limitations

This study had limitations. First, the data set contains images that were collected from a single institution. Second, we did not include annotations for calcifications and/or microcalcifications because they are notably different visual structures in the context of a computer vision detection system. Detection of calcifications was outside of our research goals when assembling this data set. This may produce a different composition of DBT volumes than typically encountered in a clinical setting. Third, the number of biopsied cases was much smaller than the number of images without bounding boxes. However, this reflects the prevalence of cancers in screening populations.

Images in the data set were interpreted by several radiologists, and the assignment of studies to groups was made, among other criteria, based on BI-RADS score, which is known to have high interreader variability. Moreover, for the first 6 to 12 months of DBT adoption at our institution, radiologists relied on both DBT and mammography for BI-RADS score assignment, and they gradually moved to diagnosis based on DBT and C-view.

Given that our criteria for a normal examination was the assessment of a radiologist for that examination, there exists a slight possibility that a cancer was detected in a follow-up examination that could then be retrospectively visible on the examination that was considered normal. However, this is a highly unlikely scenario.

Additionally, our baseline model achieved slightly better performance on test cases from the cancer group compared with the benign group. This could be explained by the fact that cancerous lesions in our data set or in general are easier to detect by a computer vision algorithm.

## Conclusions

In this study, we curated and annotated a publicly available data set of DBT volumes for future training and validation of AI tools. All the factors described previously make this data set a challenging but realistic benchmark for the future development of methods for detecting masses and architectural distortions in DBT volumes. These factors, including different types of abnormal results, exclusions of different types of cases, and different evaluation metrics, make it difficult to compare our method with those previously presented in the literature.^22,25,26^ This further underlines the importance of the data set shared in this study.

## Notes

Supplement. **eAppendix 1.** Supplemental Methods
eReferences.
**eAppendix 2.** Results From All Model Runs
