# Supplementary material for: A Data Set and Deep Learning Algorithm for the Detection of Masses and Architectural Distortions in Digital Breast Tomosynthesis Images
Source: JAMA Netw Open. 2021 Aug 16;4(8):e2119100. doi: 10.1001/jamanetworkopen.2021.19100 (PMC8369362; doi:10.1001/jamanetworkopen.2021.19100)
Supplement: Supplement. — eAppendix 1. Supplemental Methods eReferences. eAppendix 2. Results From All Model Runs [file jamanetwopen-e2119100-s001.pdf]

Supplemental Online Content

Buda M, Saha A, Walsh R, et al. A data set and deep learning algorithm for the detection of masses and architectural distortions in digital breast tomosynthesis images. *JAMA Netw Open.* 2021;4(8):e2119100. doi:10.1001/jamanetworkopen.2021.19100

eAppendix 1. Supplemental Methods

eReferences.

eAppendix 2. Results From All Model Runs

This supplemental material has been provided by the authors to give readers additional information about their work.

© 2021 Buda M et al. *JAMA Network Open.*

eAppendix 1. Supplemental Methods

1 Tested loss functions

To provide an insight into the effects of different hyper-parameters on the performance, we performed a grid search over different network sizes and objectness loss functions that address the problem of class imbalance [1]. Our problem is characterized by a significant imbalance between the bounding boxes corresponding to lesions and background class that the network learns to distinguish in the training process. The 4 tested loss functions for addressing this problem were: (i) binary cross-entropy,(ii) weighted binary cross-entropy, (iii) focal loss [2], and (iv) reduced focal loss [3]. The 4 tested loss functions are shown in Figure 1. and formulations are provided in Table 1.

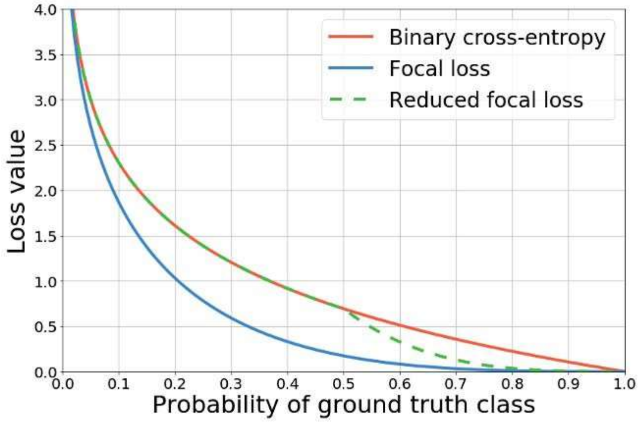

Figure 1: Loss functions tested in the grid search experiment.

Binary cross-entropy is a standard loss function used for training neural networks for binary classification problems. Weighted binary cross-entropy assigns more weight to positive examples which translates to more contribution to the model weights' (parameters) update coming from these examples. In our case, we set the weight of positive examples to the prevalence of negative examples (cellsthat do not contain an object). This way, the magnitude of contribution from positive and negative examples to the model weights' update was equalized. Focal loss function has a scaling factor that re-duces value for well classified examples ($p_t > 0.5$) and gives relatively more value for difficult examples.

© 2021 Buda M et al. *JAMA Network Open.*

Reduced focal loss is equal to binary cross entropy for values of p_t that are lower than threshold θ. Above that threshold, reduced focal loss value is smoothly reduced to focal loss, as shown in Figure 1.

Loss function	Formula
binary cross-entropy	$CE(p,y) = -\log(p_t)$, where $p_t = \begin{cases} p & \text{if } y=1 \\ 1-p & \text{otherwise} \end{cases}$
weighted binary cross-entropy	$WCE(p,y) = \begin{cases} -w \cdot \log(p_t) & \text{if } y=1 \\ -(1-w)\cdot \log(p_t) & \text{otherwise} \end{cases}$
focal loss	$FL(p,y) = -\alpha(1-p_t)^{\gamma}\log(p_t)$
reduced focal loss	$RFL(p,y) = -\alpha f(\theta)\log(p_t)$, where $f(\theta) = \begin{cases} 1 & \text{if } p_t < \theta \\ \left(\frac{1-p_t}{\theta}\right)^{\gamma} & \text{otherwise} \end{cases}$

Table 1: Formulas of the loss functions tested in the grid search experiment.

2 Hyper-parameters grid search experiment

In total, we trained 768 models and the results from all runs are provided in the Appendix A. In thehyper-parameter grid search experiments, models were trained on the biopsied cases from the training set. Since we trained the models on 2D input data (slice images), we randomly sampled slice imagescontaining ground truth boxes. Evaluation was done after every epoch on positive slice images frombiopsied cases in the validation set. For every epoch and every model, the same positive slice from each DBT volume was selected to ensure comparable performance. For each set of hyper-parameters, selection of the best snapshot of model weights was based on sensitivity at 2 false positives (FP) perslice.

For each loss function we selected the best performing model for 3D evaluation on the entire validation set. Following this 3D evaluation, the model with the highest sensitivity at 2 FPs per

© 2021 Buda M et al. *JAMA Network Open.*

DBTvolume on the validation set was used to generate predictions on the test set for the final evaluation.The entire process of selecting the final model for 3D evaluation on the test set in shown in Figure 2.

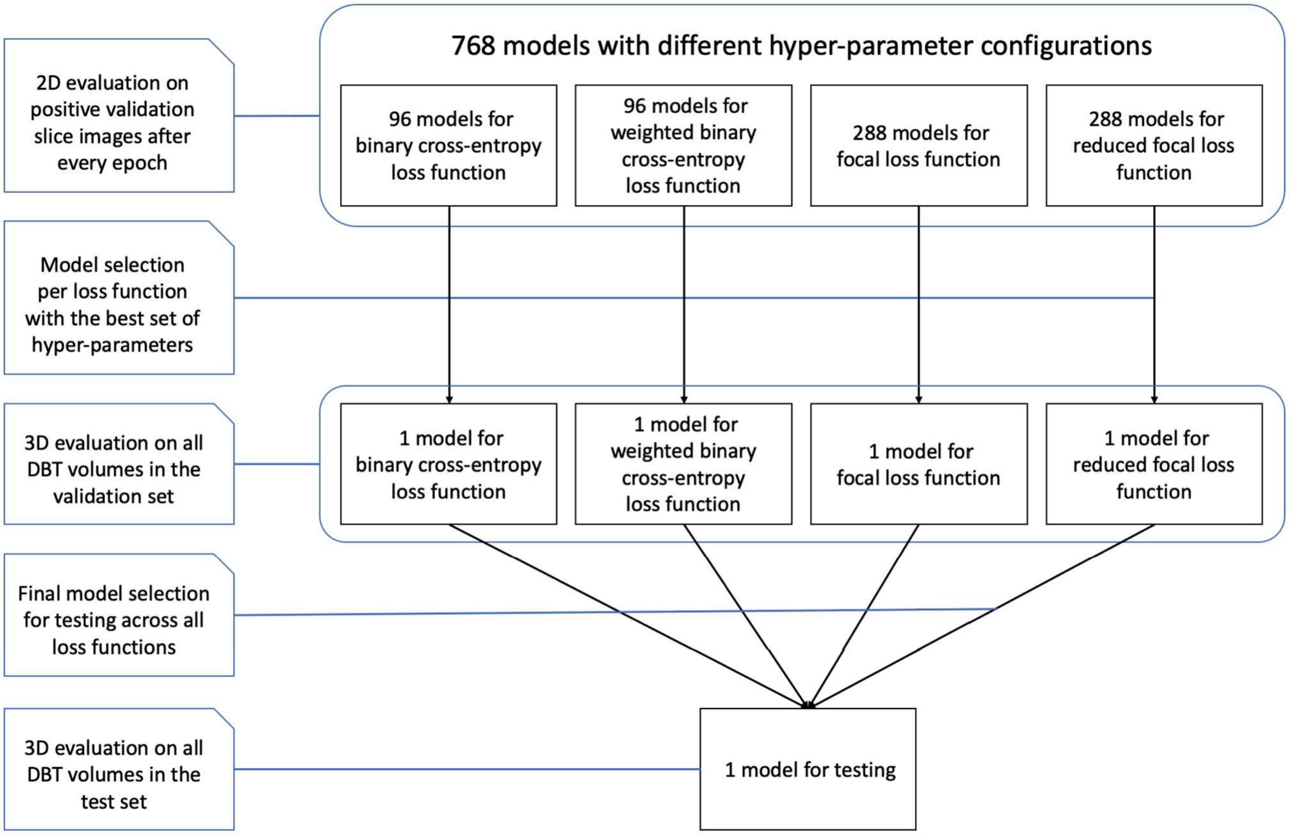

Figure 2: Model selection process.

For measuring performance of detection models we used the free-response receiver operating characteristic (FROC) curve which shows sensitivity of the model in relation to the number of false positive (FP) predictions placed in slice images, volumes, or cases. Points on the FROC curve correspond to model sensitivity and the number of FPs at different thresholds for predicted scores. Each predictedbox is associated with a score value ranging from 0.0 to 1.0. Higher score value means that a model is more confident about its prediction. For the score threshold value of 1.0, there are no predictions with predicted score higher than 1.0, which means that sensitivity is 0.0 and the number of FPs is also 0.0. We compute sensitivity and the number of FPs for all possible threshold values. As we reduce the threshold, if some of the boxes with predicted score value above the

© 2021 Buda M et al. *JAMA Network Open.*

threshold are true positive predic- tions, sensitivity increases. Similarly, as we reduce the threshold, if some of the boxes with predicted score value above the threshold are false positives, the number of FPs increases. In the 2D evaluation, the number of FPs was computed per slice image. Slice image is a cross section of DBT volume. In the 3D evaluation, the number of FPs was computed per DBT volume.

3 Results

Figure 3. shows a box plot summarizing the evaluation of different loss functions on the validation set using a 2D per-slice evaluation. All tested loss functions performed similarly with the best configuration for each loss achieving over 78% sensitivity at 2 FP per slice. Table 2. compares performance of the best model between the 4 tested loss functions.

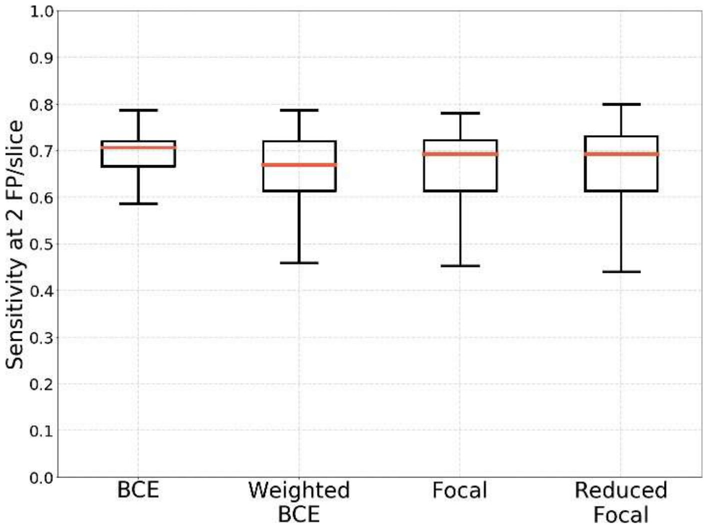

Figure 3: Comparison of different loss functions to address the class imbalance problem: the evaluation on positive cases using sensitivity at 2 FP per slice. BCE = binary cross-entropy.

Loss function	binary cross-entropy	weighted binary cross-entropy	focal loss	reduced focal loss
Sensitivity at 2 FPs per slice	79%	79%	78%	80%
Sensitivity at 2 FPs per DBT volume	59%	59%	60%	58%

Table 2: Comparison of the best models for the 4 tested loss functions on the validation set.

© 2021 Buda M et al. *JAMA Network Open.*

Using the best model from the grid search for each loss function in the 2D per-slice evaluation, we ran inference and evaluated selected models on the entire validation set using the 3D per-volume evaluation. The best performance of 60% sensitivity at 2 FP per DBT volume was achieved by the network trained using focal loss. FROC curves for the selected model on the validation set is shown in Figure 4.

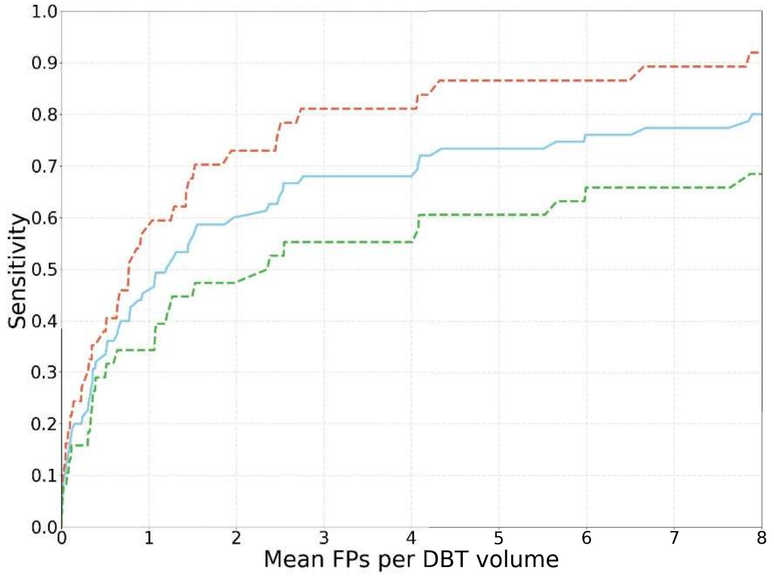

Figure 4: FROC curves showing performance on validation set of a model trained using focal loss. Red curve corresponds to cancer and not biopsied cases, green to benign and not biopsied cases, and blue curve is for allcases (biopsied and not biopsied).

4 Exclusions from initial population

Duke Health Systems' DEDUCE (Duke Enterprise Data Unified Content Explorer) tool was queried to obtain all radiology reports having the word 'tomosynthesis' and all pathology reports having the word 'breast' within the search dates of January 1, 2014 to January 30, 2018. The image download based on the study dates and medical record numbers obtained from the radiology reports resulted in an initial collection of 16 802 studies from 13 954 patients performed between August 26, 2014 and January 29, 2018 with at least one of the four reconstruction volumes: left craniocaudal (LCC), right craniocaudal (RCC), left mediolateral oblique (LMLO), right mediolateral oblique (RMLO) available. We excluded

© 2021 Buda M et al. *JAMA Network Open.*

11 192 cases that did not follow the inclusion criteria for any of the four groups. The primary reasonsfor exclusions are

- having less than four reconstruction views (for normal, actionable, and benign groups),
- BIRADS 2 and 3 (for normal, benign, and cancer groups),
- spot compression,
- imaging exams or biopsies due to the presence of calcifications/microcalcifications (for actionable, benign, and cancer groups).

Furthermore, cases were excluded for referring to multiple imaging studies/modalities (DBT + MRI, DBT + previous DBT, DBT + Ultrasound) in the radiology reports and causing ambiguity to determine if an abnormality was actually seen in the DBT. We also excluded benign, bilateral masses that end up being cysts based on comparison with previous DBT exam(s).

eReferences.

[1] Mateusz Buda, Atsuto Maki, and Maciej A Mazurowski. A systematic study of the class imbalance problem in convolutional neural networks. *Neural Networks*, 106:249–259, 2018.

[2] Tsung-Yi Lin, Priya Goyal, Ross Girshick, Kaiming He, and Piotr Dollár. Focal loss for dense object detection. In *Proceedings of the IEEE international conference on computer vision*, pages 2980–2988, 2017.

[3] Nikolay Sergievskiy and Alexander Ponamarev. Reduced focal loss: 1st place solution to xview object detection in satellite imagery. *arXiv preprint arXiv:1903.01347*, 2019.

© 2021 Buda M et al. *JAMA Network Open.*

eAppendix 2. Results From All Model Runs

Run ID	Name	Source Type	Source Name	User	Status	alpha
c3362eeadcd041779d2aa8		LOCAL	param_searc	root	FINISHED	1
27d1b997791f4e4e900774		LOCAL	param_searc	root	FINISHED	1
3805634a92274f28874baf		LOCAL	param_searc	root	FINISHED	1
9adf2bb9ffa9449694875b9		LOCAL	param_searc	root	FINISHED	1
6b86a2df065a4a7094d623		LOCAL	param_searc	root	FINISHED	1
4742e2a23ae4440187ed14		LOCAL	param_searc	root	FINISHED	1
6b013a612f254238a72f99		LOCAL	param_searc	root	FINISHED	1
35dba5f646024085af829d		LOCAL	param_searc	root	FINISHED	1
263c42eb8c36419f9de592		LOCAL	param_searc	root	FINISHED	1
9c19fff5c6d64565a368f39		LOCAL	param_searc	root	FINISHED	1
8ce4998089d74bf5890791		LOCAL	param_searc	root	FINISHED	1
8ce7edcbd3cb4c168e61f4		LOCAL	param_searc	root	FINISHED	1
76b67567f1d04518a3f13a		LOCAL	param_searc	root	FINISHED	1
d0312120ca474cdbb34640		LOCAL	param_searc	root	FINISHED	1
0a50316b9dd4435fa6b33c		LOCAL	param_searc	root	FINISHED	1
99f8a334a7af468ebf97ac9		LOCAL	param_searc	root	FINISHED	1
757178e6254843569899ec		LOCAL	param_searc	root	FINISHED	1
6205f423f890476b8e9cd9		LOCAL	param_searc	root	FINISHED	1
8e235f270ef843d49fdff58		LOCAL	param_searc	root	FINISHED	1
f4b8e7ccc41b4ac2952fc38		LOCAL	param_searc	root	FINISHED	1
fb950ac669c44de5ac55c3f		LOCAL	param_searc	root	FINISHED	1
3077ac93d2804a368fa54d		LOCAL	param_searc	root	FINISHED	1
635172a4f75c4879b414b9		LOCAL	param_searc	root	FINISHED	1
389f74481b4642018d4532		LOCAL	param_searc	root	FINISHED	1
7e8eb682369b479b89c0ca		LOCAL	param_searc	root	FINISHED	1
30ff827a9b404a17aaed5a8		LOCAL	param_searc	root	FINISHED	1
19220d97d03b4da6843e6		LOCAL	param_searc	root	FINISHED	1
08be910ee13545099917d		LOCAL	param_searc	root	FINISHED	1
e917cc5755cb4898845833		LOCAL	param_searc	root	FINISHED	1
9382030378c94c1cb4930a		LOCAL	param_searc	root	FINISHED	1
43d1b0942b5f4c18b3f930		LOCAL	param_searc	root	FINISHED	1
d873a732f6d14cbb983dec		LOCAL	param_searc	root	FINISHED	1
12652ab2d59940b593115		LOCAL	param_searc	root	FINISHED	1
ff0b2f92bc554e308cf5539		LOCAL	param_searc	root	FINISHED	1
db25dfc4dc454ac29da328		LOCAL	param_searc	root	FINISHED	1
38b839d680884a6496826		LOCAL	param_searc	root	FINISHED	1
30ffc90c556f4eb6af00c1ea		LOCAL	param_searc	root	FINISHED	1
393a2ce8c01b4d4284f4d3		LOCAL	param_searc	root	FINISHED	1

© 2021 Buda M et al. *JAMA Network Open.*

cc40e42189f94ca4aaa6dc3	LOCAL	param_searc	root	FINISHED	1
a885bb7f1f244059be8557	LOCAL	param_searc	root	FINISHED	1
5b6b3ca33c7b4ad9a6d7f6	LOCAL	param_searc	root	FINISHED	1
a28e750fa509490eb1a643	LOCAL	param_searc	root	FINISHED	1
627a1cb73a0044df8d915c	LOCAL	param_searc	root	FINISHED	1
a4b93eb1b3d74028bb935	LOCAL	param_searc	root	FINISHED	1
70f753391f0f4fbcb945fe97	LOCAL	param_searc	root	FINISHED	1
3a61761490344cc895fe34	LOCAL	param_searc	root	FINISHED	1
b6e350edf3b74cbba39adb	LOCAL	param_searc	root	FINISHED	1
2d479eb98cbf4ab7bb1ad9	LOCAL	param_searc	root	FINISHED	1
266c5a496ec640d7afe180	LOCAL	param_searc	root	FINISHED	0.5
7bdab82aba5240e6bea624	LOCAL	param_searc	root	FINISHED	0.5
546da823fb4e475c92e45e	LOCAL	param_searc	root	FINISHED	0.5
c2ff203934c34e789c91703	LOCAL	param_searc	root	FINISHED	0.5
45f0a37d440f4571bcb768	LOCAL	param_searc	root	FINISHED	0.5
acd95a73b2184124b43d6d	LOCAL	param_searc	root	FINISHED	0.5
a0a3f3149e1644bfaa0ee7c	LOCAL	param_searc	root	FINISHED	0.5
c515aaa891e24276a1fb9a	LOCAL	param_searc	root	FINISHED	0.5
4d045fedeae34ae39391e7	LOCAL	param_searc	root	FINISHED	0.5
67f9d16787cc4b32940195	LOCAL	param_searc	root	FINISHED	0.5
51fe2bec08d64ecaa041eb	LOCAL	param_searc	root	FINISHED	0.5
04df749efe7740069d82dd	LOCAL	param_searc	root	FINISHED	0.5
1d3b36bd58ff42c380d9e1	LOCAL	param_searc	root	FINISHED	0.5
268f00d7f18c4f1c82a0a73	LOCAL	param_searc	root	FINISHED	0.5
032b076d16f34ee093b4f8	LOCAL	param_searc	root	FINISHED	0.5
1099dfd8380c45fca47c3e9	LOCAL	param_searc	root	FINISHED	0.5
cf88f5138668461ab38f7fd	LOCAL	param_searc	root	FINISHED	0.5
62d4595854234069b164f0	LOCAL	param_searc	root	FINISHED	0.5
0a1ca9772d0444cfbe9bc6	LOCAL	param_searc	root	FINISHED	0.5
ceb0bb72b0054e03a6d451	LOCAL	param_searc	root	FINISHED	0.5
b644e3b0b60f4331b59141	LOCAL	param_searc	root	FINISHED	0.5
dca5f1b39b3443788d188c	LOCAL	param_searc	root	FINISHED	0.5
f404b49279384531ad496e	LOCAL	param_searc	root	FINISHED	0.5
76a57a6975eb4546a019f3	LOCAL	param_searc	root	FINISHED	0.5
7ec9b26d953646848112f5	LOCAL	param_searc	root	FINISHED	0.5
552a810f06e24012871e5d	LOCAL	param_searc	root	FINISHED	0.5
9d1f4d172e114991abe882	LOCAL	param_searc	root	FINISHED	0.5
18d67db069544c63860746	LOCAL	param_searc	root	FINISHED	0.5
816b7a7c6f8f435cbebdcd8	LOCAL	param_searc	root	FINISHED	0.5
e778e05e922b45ae9d55d4	LOCAL	param_searc	root	FINISHED	0.5
60d6edb5432146ac815b33	LOCAL	param_searc	root	FINISHED	0.5

© 2021 Buda M et al. *JAMA Network Open.*

4bfc1701ba73449eb3f041	LOCAL	param_searc	root	FINISHED	0.5
d7d75186becb4f37ba26a3	LOCAL	param_searc	root	FINISHED	0.5
dca447e2a7c2495089c88ff	LOCAL	param_searc	root	FINISHED	0.5
5f60f43889b846abb20884	LOCAL	param_searc	root	FINISHED	0.5
06d1d1109e844ebebe54e	LOCAL	param_searc	root	FINISHED	0.5
ff03f455abbc4d6f83b1ae3	LOCAL	param_searc	root	FINISHED	0.5
095d9f83f66f4c8a85f5349	LOCAL	param_searc	root	FINISHED	0.5
6d479f9639344eb7bf9902	LOCAL	param_searc	root	FINISHED	0.5
084503d8d79941ffb184d1	LOCAL	param_searc	root	FINISHED	0.5
6211b85006fb4e17909fb7	LOCAL	param_searc	root	FINISHED	0.5
327ac14c9bd046c28e1f05	LOCAL	param_searc	root	FINISHED	0.5
890a4ec00b6845b1a919d0	LOCAL	param_searc	root	FINISHED	0.5
7a520e8de7f94a7f881723	LOCAL	param_searc	root	FINISHED	0.5
7aae6ca24de744469bcef7	LOCAL	param_searc	root	FINISHED	0.5
7ad2a265ed3649318b7814	LOCAL	param_searc	root	FINISHED	0.5
b09cc16ba50b43ec857301	LOCAL	param_searc	root	FINISHED	0.5
27af3c043b1c4228932794	LOCAL	param_searc	root	FINISHED	0.5
5305c69a84454d458ed608	LOCAL	param_searc	root	FINISHED	0.25
aa64f571a2194e32ab1825	LOCAL	param_searc	root	FINISHED	0.25
15f12c293295468a91445c	LOCAL	param_searc	root	FINISHED	0.25
3aa042f26a6e43988b7756	LOCAL	param_searc	root	FINISHED	0.25
8d1269b65c474bec8595ac	LOCAL	param_searc	root	FINISHED	0.25
e24e50ad0b0742ee8c775f	LOCAL	param_searc	root	FINISHED	0.25
a876968047ed4670b86016	LOCAL	param_searc	root	FINISHED	0.25
6867b9ea6f814db580d6d4	LOCAL	param_searc	root	FINISHED	0.25
ea8f15b475dc4758974e7f	LOCAL	param_searc	root	FINISHED	0.25
e1c0a26d48cb44c19f7f442	LOCAL	param_searc	root	FINISHED	0.25
eba44e0bbe9546e79a0d7f	LOCAL	param_searc	root	FINISHED	0.25
4ca787edb6124feda4a209	LOCAL	param_searc	root	FINISHED	0.25
350fac73a2054312820511	LOCAL	param_searc	root	FINISHED	0.25
fad63c8e6cce48cc824745a	LOCAL	param_searc	root	FINISHED	0.25
b1367900d41547f1a2c917	LOCAL	param_searc	root	FINISHED	0.25
9c70df4efead43c3b57ae2e	LOCAL	param_searc	root	FINISHED	0.25
9af7559d38464cebbe9a10	LOCAL	param_searc	root	FINISHED	0.25
76ab910f7d524664851146	LOCAL	param_searc	root	FINISHED	0.25
abf235710514440eab8c46	LOCAL	param_searc	root	FINISHED	0.25
5fcc0506e76542ab8576f96	LOCAL	param_searc	root	FINISHED	0.25
d77c1ec2f7914c19882b54	LOCAL	param_searc	root	FINISHED	0.25
5b81d7d580c2437b8b863f	LOCAL	param_searc	root	FINISHED	0.25
314323f6687d4af1bd78a2	LOCAL	param_searc	root	FINISHED	0.25
50653730c14f4bc391780e	LOCAL	param_searc	root	FINISHED	0.25

© 2021 Buda M et al. *JAMA Network Open.*

bde1a42dec764ab7b454bc	LOCAL	param_searc	root	FINISHED	0.25
185fd3407e9b46918929ea	LOCAL	param_searc	root	FINISHED	0.25
3a8862bd16b54ea78b33b	LOCAL	param_searc	root	FINISHED	0.25
ab68800db9f247ba9b957e	LOCAL	param_searc	root	FINISHED	0.25
e2f5f25300d640e9a9fc1bd	LOCAL	param_searc	root	FINISHED	0.25
51d20db1c4cd4af5b10939	LOCAL	param_searc	root	FINISHED	0.25
ea0f73f336bb44108c3949	LOCAL	param_searc	root	FINISHED	0.25
d0697c1b1fd34bc9a0230e	LOCAL	param_searc	root	FINISHED	0.25
80f4af4b1f4d4d92b1602a3	LOCAL	param_searc	root	FINISHED	0.25
b74f75c92d0b490c8dceaf6	LOCAL	param_searc	root	FINISHED	0.25
38ec0b9968f74747803cf71	LOCAL	param_searc	root	FINISHED	0.25
d9a4c0c608fc4de093859ca	LOCAL	param_searc	root	FINISHED	0.25
ee1964bdb95d4fb4941379	LOCAL	param_searc	root	FINISHED	0.25
4fc733d48e9444d594dc86	LOCAL	param_searc	root	FINISHED	0.25
4eaa1633df224646910cc1	LOCAL	param_searc	root	FINISHED	0.25
26dd0203adeb4e7a98a536	LOCAL	param_searc	root	FINISHED	0.25
fb88ec4eafd54693b46d7cd	LOCAL	param_searc	root	FINISHED	0.25
cbf9ad209c5f4e99b4ad305	LOCAL	param_searc	root	FINISHED	0.25
1e997a80f2fe4227a14eb1	LOCAL	param_searc	root	FINISHED	0.25
3f20e97714694552820796	LOCAL	param_searc	root	FINISHED	0.25
73fb69e1f7dd484eaea1f5e	LOCAL	param_searc	root	FINISHED	0.25
0b89a082569847e790cbf7	LOCAL	param_searc	root	FINISHED	0.25
75b4cfabac0a4e53a72237	LOCAL	param_searc	root	FINISHED	0.25
8a0cd824c98048f7b14f3a8	LOCAL	param_searc	root	FINISHED	0.25
0a8eca50aa8e46f192ddb9	LOCAL	param_searc	root	FINISHED	1
7b4154607faa411ca084cf5	LOCAL	param_searc	root	FINISHED	1
1dc5584a02954131a0df79	LOCAL	param_searc	root	FINISHED	1
8acb0ec616e24c23aab7a8	LOCAL	param_searc	root	FINISHED	1
b8f080b0ff23430bba33572	LOCAL	param_searc	root	FINISHED	1
6d976fa80f0d46018804e3	LOCAL	param_searc	root	FINISHED	1
90e90be7e2fa4e2db54e8d	LOCAL	param_searc	root	FINISHED	1
f4cdae54272f4e31b40122	LOCAL	param_searc	root	FINISHED	1
a4fe06033ad9473b833d22	LOCAL	param_searc	root	FINISHED	1
050b625f4821493da612c5	LOCAL	param_searc	root	FINISHED	1
d06ee5d3bb264120bdfbc9	LOCAL	param_searc	root	FINISHED	1
700960bb6a4943c68458c7	LOCAL	param_searc	root	FINISHED	1
2d2ff2f37439498bb7b3d4f	LOCAL	param_searc	root	FINISHED	1
4e19c686ed8f477683a1ee	LOCAL	param_searc	root	FINISHED	1
f4b17465e9df4039ab48fe8	LOCAL	param_searc	root	FINISHED	1
5ebf7fe1365c47f898d69d6	LOCAL	param_searc	root	FINISHED	1
a33885dd956b40b8872fe1	LOCAL	param_searc	root	FINISHED	1

© 2021 Buda M et al. *JAMA Network Open.*

8490709fa4614c8da8068f1	LOCAL	param_searc	root	FINISHED	1
5b92ce13eb4f44cd9d4f112	LOCAL	param_searc	root	FINISHED	1
bc58b8cb41e94b47b8ef3f	LOCAL	param_searc	root	FINISHED	1
0a09a6b257e74eb38bfe7f	LOCAL	param_searc	root	FINISHED	1
3026bff587ac413f8fe1310	LOCAL	param_searc	root	FINISHED	1
67f4b3e4138743df938823	LOCAL	param_searc	root	FINISHED	1
defa5d2095304935abf799	LOCAL	param_searc	root	FINISHED	1
776115a1f4a24beabbf660	LOCAL	param_searc	root	FINISHED	1
6fb580613ff140ff8f245866	LOCAL	param_searc	root	FINISHED	1
f29c62e84b55410fa86e415	LOCAL	param_searc	root	FINISHED	1
13e79c43f58d4706afdd47	LOCAL	param_searc	root	FINISHED	1
fcad71537da34ccd9a72f09	LOCAL	param_searc	root	FINISHED	1
da7dbfddb8eb4a95ba6941	LOCAL	param_searc	root	FINISHED	1
fbff5b634ae24b1b99f34bd	LOCAL	param_searc	root	FINISHED	1
859b315bf1f54850af7c01d	LOCAL	param_searc	root	FINISHED	1
cf966aaeb42b429ba49c9e	LOCAL	param_searc	root	FINISHED	1
6f10749e4b3948e3a96a44	LOCAL	param_searc	root	FINISHED	1
106804ce8844448e85a49d	LOCAL	param_searc	root	FINISHED	1
e8f626435a434875b37f72	LOCAL	param_searc	root	FINISHED	1
722d031698cd4714bc5f35	LOCAL	param_searc	root	FINISHED	1
0df1f48ce3fc4d989d41e4a	LOCAL	param_searc	root	FINISHED	1
cda1ded12a224b4ebd5d40	LOCAL	param_searc	root	FINISHED	1
c2920c5ed16c4574a74d00	LOCAL	param_searc	root	FINISHED	1
c9d3e0e62e32426992c993	LOCAL	param_searc	root	FINISHED	1
dfb391a1ee5f4e309ba36b	LOCAL	param_searc	root	FINISHED	1
31e188aa533a4670a26a28	LOCAL	param_searc	root	FINISHED	1
742b8712d5744e029cd3ef	LOCAL	param_searc	root	FINISHED	1
fdd51605fc8c40afaf127c60	LOCAL	param_searc	root	FINISHED	1
37102db1173e424195bb8	LOCAL	param_searc	root	FINISHED	1
b4adb25fc1284176bd3589	LOCAL	param_searc	root	FINISHED	1
a8d002cbe17d457db180fa	LOCAL	param_searc	root	FINISHED	1
8df30679831248869f7bdd	LOCAL	param_searc	root	FINISHED	0.5
ec4b85398d3f409c8bad8c	LOCAL	param_searc	root	FINISHED	0.5
41337da9f96d4f0ba02e1b	LOCAL	param_searc	root	FINISHED	0.5
d4756eef0164467ab9323c	LOCAL	param_searc	root	FINISHED	0.5
382809e3a2f040f9bbf9fb7	LOCAL	param_searc	root	FINISHED	0.5
3d62328a1c134a93a1e92e	LOCAL	param_searc	root	FINISHED	0.5
f809941de16a456f9fe5943	LOCAL	param_searc	root	FINISHED	0.5
006997a115404c948f119a	LOCAL	param_searc	root	FINISHED	0.5
f3299c1e575b4b1a836e7e	LOCAL	param_searc	root	FINISHED	0.5
34c51b613a7142e5b3f6e0	LOCAL	param_searc	root	FINISHED	0.5

© 2021 Buda M et al. *JAMA Network Open.*

2c8d9dd734fb4581a8a819	LOCAL	param_searc	root	FINISHED	0.5
36cfc177c68d4679a39324f	LOCAL	param_searc	root	FINISHED	0.5
58c2dfdd81d54b36b16834	LOCAL	param_searc	root	FINISHED	0.5
60965e150ae243448ae267	LOCAL	param_searc	root	FINISHED	0.5
7413bffe794744389ef8c2c	LOCAL	param_searc	root	FINISHED	0.5
39fd92f8f4174a58a1303ac	LOCAL	param_searc	root	FINISHED	0.5
67fcbe5ccd1a44e19e3781f	LOCAL	param_searc	root	FINISHED	0.5
e844cc6b0ece4b32a56763	LOCAL	param_searc	root	FINISHED	0.5
25bf56b7e40748ee9cc77e	LOCAL	param_searc	root	FINISHED	0.5
9f07883129c840a48aa422	LOCAL	param_searc	root	FINISHED	0.5
0b7a23cb763c43d1a6ac41	LOCAL	param_searc	root	FINISHED	0.5
46d2a1a6da61485f8a6e46	LOCAL	param_searc	root	FINISHED	0.5
c74cd70d339f4ce9b1ac866	LOCAL	param_searc	root	FINISHED	0.5
29f4cc2a1c9d4b88b7a21d	LOCAL	param_searc	root	FINISHED	0.5
520e026b1ac443eebdc737	LOCAL	param_searc	root	FINISHED	0.5
f4f763c1dd954fb7b6f5f8d	LOCAL	param_searc	root	FINISHED	0.5
b3f7a417bc5848e5b5317b	LOCAL	param_searc	root	FINISHED	0.5
12eca17e85824f33af0c022	LOCAL	param_searc	root	FINISHED	0.5
f5f2cebd350b4b1abd363c	LOCAL	param_searc	root	FINISHED	0.5
b8c318e410c549a4abd76a	LOCAL	param_searc	root	FINISHED	0.5
d8b2e940cd3f44e88fd698	LOCAL	param_searc	root	FINISHED	0.5
1dc9e221082f4c39b97e95	LOCAL	param_searc	root	FINISHED	0.5
fe2eb039a76d451196df8c	LOCAL	param_searc	root	FINISHED	0.5
85586388a62a411b8d9352	LOCAL	param_searc	root	FINISHED	0.5
3d7c929fd3284ade9a3956	LOCAL	param_searc	root	FINISHED	0.5
9b8268b5dce946f687cfcdf	LOCAL	param_searc	root	FINISHED	0.5
ccdd54c649594b5a931ca8	LOCAL	param_searc	root	FINISHED	0.5
8159fc75aafc40bf8f1c2744	LOCAL	param_searc	root	FINISHED	0.5
e6f569f3161f4d2b9d50480	LOCAL	param_searc	root	FINISHED	0.5
82461a9e1786452c9aa194	LOCAL	param_searc	root	FINISHED	0.5
c503102507fb48d2960ecc	LOCAL	param_searc	root	FINISHED	0.5
bb5a5d26d1bc4b83ab512f	LOCAL	param_searc	root	FINISHED	0.5
21333efdabe14182bf308d	LOCAL	param_searc	root	FINISHED	0.5
209b1e2f17b840a5bc2743	LOCAL	param_searc	root	FINISHED	0.5
7ad90ec840cb44f7a78a27	LOCAL	param_searc	root	FINISHED	0.5
e85480c4a17c40bd8ea480	LOCAL	param_searc	root	FINISHED	0.5
d42f1bf3213a40c58e46cb2	LOCAL	param_searc	root	FINISHED	0.5
038714449fa849d290cec5	LOCAL	param_searc	root	FINISHED	0.5
097b159327d34eb5aff367	LOCAL	param_searc	root	FINISHED	0.25
df056d8bfe4946b4be8c71	LOCAL	param_searc	root	FINISHED	0.25
7f795f3bc343420ba801d2	LOCAL	param_searc	root	FINISHED	0.25

© 2021 Buda M et al. *JAMA Network Open.*

2779f018914a4d989b6d75	LOCAL	param_searc	root	FINISHED	0.25
7c487fd50caa4c89b8c1a14	LOCAL	param_searc	root	FINISHED	0.25
f1037a5e9e924c9babb9d1	LOCAL	param_searc	root	FINISHED	0.25
eaf8c09f33b54221898b97	LOCAL	param_searc	root	FINISHED	0.25
c0da7e166c8846bb817cd0	LOCAL	param_searc	root	FINISHED	0.25
9f3ed860f94445f8b3a3ac6	LOCAL	param_searc	root	FINISHED	0.25
7fb21d3e1bbc44c3b66dffd	LOCAL	param_searc	root	FINISHED	0.25
745b37790c144520995874	LOCAL	param_searc	root	FINISHED	0.25
9fa2e998b5264164941fd4	LOCAL	param_searc	root	FINISHED	0.25
7f5e7d3c9f5b49e0934d7d	LOCAL	param_searc	root	FINISHED	0.25
b74eb3313f9843f390f5097	LOCAL	param_searc	root	FINISHED	0.25
2096f1ca541741659bf00a6	LOCAL	param_searc	root	FINISHED	0.25
b8973f4b662d4f65b8f5c42	LOCAL	param_searc	root	FINISHED	0.25
6a568125e682466fa0f3b4	LOCAL	param_searc	root	FINISHED	0.25
bed82b4696e84f4e9de554	LOCAL	param_searc	root	FINISHED	0.25
d8c87bbf3e654ad7a0cd7f6	LOCAL	param_searc	root	FINISHED	0.25
5432e370a4b04cc8ad26db	LOCAL	param_searc	root	FINISHED	0.25
4b0507dff001467a88ab35	LOCAL	param_searc	root	FINISHED	0.25
de34848405294143ba910a	LOCAL	param_searc	root	FINISHED	0.25
3b69d0e735844e0aa94f69	LOCAL	param_searc	root	FINISHED	0.25
be73d49989f145b1b9ec3d	LOCAL	param_searc	root	FINISHED	0.25
ca6366af4b0f42daab81fa0	LOCAL	param_searc	root	FINISHED	0.25
df87fb3720fa430aadc1285	LOCAL	param_searc	root	FINISHED	0.25
bc5d76d546364c2bafc2a8	LOCAL	param_searc	root	FINISHED	0.25
64f721f05d5842c68981c8d	LOCAL	param_searc	root	FINISHED	0.25
07dff55b70ab463d8030a3	LOCAL	param_searc	root	FINISHED	0.25
6c04dd9e34a44fa2b1e505	LOCAL	param_searc	root	FINISHED	0.25
a7ce65caed374629b9592b	LOCAL	param_searc	root	FINISHED	0.25
bd9e3a54ddc640df8067b1	LOCAL	param_searc	root	FINISHED	0.25
61b5be258f464043a6b215	LOCAL	param_searc	root	FINISHED	0.25
72513658ec974beab2e039	LOCAL	param_searc	root	FINISHED	0.25
0b7cac65565f4328879582	LOCAL	param_searc	root	FINISHED	0.25
5e5dcbecb0b546a2944bfa	LOCAL	param_searc	root	FINISHED	0.25
efbc54be26d74927b7d87b	LOCAL	param_searc	root	FINISHED	0.25
b098d2a40495423db0f132	LOCAL	param_searc	root	FINISHED	0.25
f5a905dffbfc48a49271a09f	LOCAL	param_searc	root	FINISHED	0.25
866e037a96e54193be1bc8	LOCAL	param_searc	root	FINISHED	0.25
d4d79a3c74144a7f9158eb	LOCAL	param_searc	root	FINISHED	0.25
0d57295492214e1abfc304	LOCAL	param_searc	root	FINISHED	0.25
adafd0ec085749a6bf44699	LOCAL	param_searc	root	FINISHED	0.25
b505be9d9dcd45afa463a5	LOCAL	param_searc	root	FINISHED	0.25

© 2021 Buda M et al. *JAMA Network Open.*

b0b4476c41c041ebac0472	LOCAL	param_searc	root	FINISHED	0.25
5645a649793d495fb8f484	LOCAL	param_searc	root	FINISHED	0.25
8398172e4f044ee4a5ae9f	LOCAL	param_searc	root	FINISHED	0.25
90b3d9d091484c568e308d	LOCAL	param_searc	root	FINISHED	0.25
51f721ef37084faea722292	LOCAL	param_searc	root	FINISHED	1
dabea235ad444efc8ce168	LOCAL	param_searc	root	FINISHED	1
c9256f1d14af4792918b11	LOCAL	param_searc	root	FINISHED	1
6777a7c536e34621a1878d	LOCAL	param_searc	root	FINISHED	1
9eb610bbbaa443b39a8ef0	LOCAL	param_searc	root	FINISHED	1
760b4001de084560ba069	LOCAL	param_searc	root	FINISHED	1
0e39ca61767548839350fc	LOCAL	param_searc	root	FINISHED	1
3b306ab55b5946a592b18	LOCAL	param_searc	root	FINISHED	1
6f2f501c84d043468188d9	LOCAL	param_searc	root	FINISHED	1
999742841f05470f8ddd66	LOCAL	param_searc	root	FINISHED	1
52e19f2e45dc4dc7bf08868	LOCAL	param_searc	root	FINISHED	1
802dc632d413411f9c06a1	LOCAL	param_searc	root	FINISHED	1
3c597673b7ea4d929ed181	LOCAL	param_searc	root	FINISHED	1
838595b1bef34769aee3cc	LOCAL	param_searc	root	FINISHED	1
89ddce2d4746457c8bc444	LOCAL	param_searc	root	FINISHED	1
bd410d7f85ff411ba22d791	LOCAL	param_searc	root	FINISHED	1
f7221b398199464aa09d3a	LOCAL	param_searc	root	FINISHED	1
eacb3c428e80448f9b753c	LOCAL	param_searc	root	FINISHED	1
a4b77182d45c499ea6e5dc	LOCAL	param_searc	root	FINISHED	1
4bcaef15dceb46c9925674	LOCAL	param_searc	root	FINISHED	1
66e6739f3ea24062a65316	LOCAL	param_searc	root	FINISHED	1
be60be3ec6c445a7a6bdb5	LOCAL	param_searc	root	FINISHED	1
6638d16e038442a2a3e8d1	LOCAL	param_searc	root	FINISHED	1
6d8b9faa6e3945beb96843	LOCAL	param_searc	root	FINISHED	1
36471c8aedba4aecbadd82	LOCAL	param_searc	root	FINISHED	1
77ddccf6a1ad4490a0d71f7	LOCAL	param_searc	root	FINISHED	1
239e4d0c0ee7425fa40501	LOCAL	param_searc	root	FINISHED	1
aa77962b19fe4007b8d897	LOCAL	param_searc	root	FINISHED	1
ebbde2379ac947e9be8634	LOCAL	param_searc	root	FINISHED	1
5d52d21fb66e49a8b2eeac	LOCAL	param_searc	root	FINISHED	1
c53e45a3fe424a69af40d64	LOCAL	param_searc	root	FINISHED	1
ada677ecc3ce467884d873	LOCAL	param_searc	root	FINISHED	1
77fa81ffb7c44af596a4ca68	LOCAL	param_searc	root	FINISHED	0.5
5b37d5ca4049435cb32f01	LOCAL	param_searc	root	FINISHED	0.5
790dd0cf59ac4d1e9794a1	LOCAL	param_searc	root	FINISHED	0.5
5c3a0c48c7d54b2fab9851	LOCAL	param_searc	root	FINISHED	0.5
fc433a5340624960855091	LOCAL	param_searc	root	FINISHED	0.5

© 2021 Buda M et al. *JAMA Network Open.*

998dba3607854c7ea43812	LOCAL	param_searc root	FINISHED	0.5
06d0010f6ee246628591f1	LOCAL	param_searc root	FINISHED	0.5
bffb1843445f4c0ea31b96a	LOCAL	param_searc root	FINISHED	0.5
2d77ece324b74f688b2ee9	LOCAL	param_searc root	FINISHED	0.5
4c55865ae91b4642b3d25c	LOCAL	param_searc root	FINISHED	0.5
a04419a41f2a40d6953325	LOCAL	param_searc root	FINISHED	0.5
a255c94af5c6468295a017	LOCAL	param_searc root	FINISHED	0.5
2161cd5f710f47b9bfc823e	LOCAL	param_searc root	FINISHED	0.5
3ecfff5321064913914ff4f6	LOCAL	param_searc root	FINISHED	0.5
2c41872dfb28441b8458ac	LOCAL	param_searc root	FINISHED	0.5
f894f73748bf4c7796547d1	LOCAL	param_searc root	FINISHED	0.5
86956af10aed4c4581287b	LOCAL	param_searc root	FINISHED	0.5
58653aa5790849c6ada70f	LOCAL	param_searc root	FINISHED	0.5
30b7340245614985a384b	LOCAL	param_searc root	FINISHED	0.5
6819418ce1dc4494a8a318	LOCAL	param_searc root	FINISHED	0.5
c5ff03cc3b2443ac98d4700	LOCAL	param_searc root	FINISHED	0.5
57178e0b1d0546eb82eda	LOCAL	param_searc root	FINISHED	0.5
235c6c582c0a44e8bf5ab5	LOCAL	param_searc root	FINISHED	0.5
035bf80cabbe4a88806444	LOCAL	param_searc root	FINISHED	0.5
bc997e8dc37d4dfc9b6010	LOCAL	param_searc root	FINISHED	0.5
897596c769ff43f0b0d10e3	LOCAL	param_searc root	FINISHED	0.5
a6f2bad854da403db59cd3	LOCAL	param_searc root	FINISHED	0.5
a74dc66df3564dbcb6fd25	LOCAL	param_searc root	FINISHED	0.5
95277920d62b4496ba5bc4	LOCAL	param_searc root	FINISHED	0.5
384e170820ae46209f89a8	LOCAL	param_searc root	FINISHED	0.5
017533249b93432d934c59	LOCAL	param_searc root	FINISHED	0.5
a7b5b1d40fe04a46bbc81a	LOCAL	param_searc root	FINISHED	0.5
1e44d5d1add14b4bb3a35	LOCAL	param_searc root	FINISHED	0.25
6fe5137ca72045cca5d8e51	LOCAL	param_searc root	FINISHED	0.25
4abd90dace2442ec95b370	LOCAL	param_searc root	FINISHED	0.25
8d0ca55f257e49e7a8e344	LOCAL	param_searc root	FINISHED	0.25
d5a91efbffca427b805d513	LOCAL	param_searc root	FINISHED	0.25
7a46cb5ba76a4db599386f	LOCAL	param_searc root	FINISHED	0.25
60cf905235864df7b0bceb6	LOCAL	param_searc root	FINISHED	0.25
c7712893c805428b8f8e37	LOCAL	param_searc root	FINISHED	0.25
300fb76a35234879b07246	LOCAL	param_searc root	FINISHED	0.25
07501dd1714044fc9e1f36	LOCAL	param_searc root	FINISHED	0.25
dc10eff6772c4e07bc0b233	LOCAL	param_searc root	FINISHED	0.25
f9539d2b9e684bfb940c21	LOCAL	param_searc root	FINISHED	0.25
543f4451740d4e43870b66	LOCAL	param_searc root	FINISHED	0.25
9164b886a9994889a68a51	LOCAL	param_searc root	FINISHED	0.25

© 2021 Buda M et al. *JAMA Network Open.*

1f5837a0dd08426eb2c460	LOCAL	param_searc	root	FINISHED	0.25
28d3ad6f76a54046adb4ab	LOCAL	param_searc	root	FINISHED	0.25
07a7534715574a748dfc84	LOCAL	param_searc	root	FINISHED	0.25
89f2871c386742d3a1ac0e	LOCAL	param_searc	root	FINISHED	0.25
a3ea32efe6bb4452b92029	LOCAL	param_searc	root	FINISHED	0.25
12d1a6f490794095aee079	LOCAL	param_searc	root	FINISHED	0.25
ad71d801df4c445ebca26c	LOCAL	param_searc	root	FINISHED	0.25
d6da76d281e04e8cae929a	LOCAL	param_searc	root	FINISHED	0.25
022d8c75b45b4540b00a6e	LOCAL	param_searc	root	FINISHED	0.25
d0820887cba7426a9201ea	LOCAL	param_searc	root	FINISHED	0.25
9d662b83a4b24f9594f5ba	LOCAL	param_searc	root	FINISHED	0.25
50daea21cadb4024bdb054	LOCAL	param_searc	root	FINISHED	0.25
f34e14ce756a4d15baf22d2	LOCAL	param_searc	root	FINISHED	0.25
c54d56c87a72491ebd39e3	LOCAL	param_searc	root	FINISHED	0.25
4508ebd62b074a1880f84e	LOCAL	param_searc	root	FINISHED	0.25
433f2ea68ae2413bb37c96	LOCAL	param_searc	root	FINISHED	0.25
b95ff1baf76d4f1eb57f5a9	LOCAL	param_searc	root	FINISHED	0.25
ed18e9d4a65f41e5b07e25	LOCAL	param_searc	root	FINISHED	0.25
59dde7f6311744d2b3c325	LOCAL	param_searc	root	FINISHED	1
6419ac33c57a4b45bc8673	LOCAL	param_searc	root	FINISHED	1
73a30f5badaa4bd8a69df2	LOCAL	param_searc	root	FINISHED	1
26f4c6b20d8c47f1a36d9f0	LOCAL	param_searc	root	FINISHED	1
436f55e080fe45a5a85cf4a	LOCAL	param_searc	root	FINISHED	1
7e7774413ff44564935c04	LOCAL	param_searc	root	FINISHED	1
8c8c4bcbd7414c0488c9dc	LOCAL	param_searc	root	FINISHED	1
c1bbfd98846f498aac464e4	LOCAL	param_searc	root	FINISHED	1
4c51dd37683140a0a98f89	LOCAL	param_searc	root	FINISHED	1
5ffab48b87e34cb0bf2f890	LOCAL	param_searc	root	FINISHED	1
a4bb5dbc49474ab99cb22b	LOCAL	param_searc	root	FINISHED	1
fdc650f50174420d86e3c89	LOCAL	param_searc	root	FINISHED	1
4ea7c220ec084ff99a69622	LOCAL	param_searc	root	FINISHED	1
2f737cb7e52e4f16a65a15d	LOCAL	param_searc	root	FINISHED	1
ad3261f7758a4decb36887	LOCAL	param_searc	root	FINISHED	1
0720973e01ba429284f382	LOCAL	param_searc	root	FINISHED	1
9ffd85da9303455cb9191d	LOCAL	param_searc	root	FINISHED	1
951ce1056bfe472887550b	LOCAL	param_searc	root	FINISHED	1
9df096b115e64524b00c21	LOCAL	param_searc	root	FINISHED	1
13c8fcbf345541818cee8dd	LOCAL	param_searc	root	FINISHED	1
d8c3f4a785ec4dcdb5325d	LOCAL	param_searc	root	FINISHED	1
e679a33a1d334c31bc3b44	LOCAL	param_searc	root	FINISHED	1
db563ca78d0346e1a4a270	LOCAL	param_searc	root	FINISHED	1

© 2021 Buda M et al. *JAMA Network Open.*

e1ebcf5007cf48ef8485034	LOCAL	param_searc	root	FINISHED	1
d213866d1733487e8f7bd5	LOCAL	param_searc	root	FINISHED	1
41f17a704d824cc2a06978	LOCAL	param_searc	root	FINISHED	1
4b88ebb2d7a7405d9194a	LOCAL	param_searc	root	FINISHED	1
66163b3c0c464bcca0cf539	LOCAL	param_searc	root	FINISHED	1
af262f9b63644cf388597e7	LOCAL	param_searc	root	FINISHED	1
7ff5f8153a2546e8991bb61	LOCAL	param_searc	root	FINISHED	1
c2ba87def7184769a6171f7	LOCAL	param_searc	root	FINISHED	1
3fbbea3cce124042901f455	LOCAL	param_searc	root	FINISHED	1
8a45a6e1bff44eaab32a84d	LOCAL	param_searc	root	FINISHED	1
ace835b580bc41518c7375	LOCAL	param_searc	root	FINISHED	1
1d605b5d72824a5a816582	LOCAL	param_searc	root	FINISHED	1
f5216a3010be41ba9b07e6	LOCAL	param_searc	root	FINISHED	1
d01b7c0dabc24b3b87dbce	LOCAL	param_searc	root	FINISHED	1
b80ad095a3d944a7a2143d	LOCAL	param_searc	root	FINISHED	1
3ddb21adb4ff49b3b3e30d	LOCAL	param_searc	root	FINISHED	1
3e126b0f8e1c4225a2aff61	LOCAL	param_searc	root	FINISHED	1
ce25d922b7d642ba84a8e2	LOCAL	param_searc	root	FINISHED	1
e99cc71e712b454b900e3d	LOCAL	param_searc	root	FINISHED	1
46fe5826447e41a3a2f686	LOCAL	param_searc	root	FINISHED	1
e0c5dd65e44a4643bff7fbd	LOCAL	param_searc	root	FINISHED	1
e764df1ea991485490b45f	LOCAL	param_searc	root	FINISHED	1
0e2abe4d6eae4b16b42f51	LOCAL	param_searc	root	FINISHED	1
908b40a4732f496486f73f6	LOCAL	param_searc	root	FINISHED	1
7b9ac44fe2b04d259ed539	LOCAL	param_searc	root	FINISHED	1
cd686ee1ac414d31ac2750	LOCAL	param_searc	root	FINISHED	0.5
f7d4ae5621eb4eae8d34c8	LOCAL	param_searc	root	FINISHED	0.5
e894659c34c2416a8cd100	LOCAL	param_searc	root	FINISHED	0.5
73b94414413c4d6c8cfe99	LOCAL	param_searc	root	FINISHED	0.5
59518148527f4f38b94951	LOCAL	param_searc	root	FINISHED	0.5
e65b8fcdf14b4f37b99a7ae	LOCAL	param_searc	root	FINISHED	0.5
8b8cbffac58840e2b2b3629	LOCAL	param_searc	root	FINISHED	0.5
2cffd8e9967b4f068b96326	LOCAL	param_searc	root	FINISHED	0.5
08697b6fee834dcdb8d13a	LOCAL	param_searc	root	FINISHED	0.5
b0cd336ff04f48dfadc31e5	LOCAL	param_searc	root	FINISHED	0.5
276c6f18d20f48cdb644bd	LOCAL	param_searc	root	FINISHED	0.5
632103379665469fb76e3c	LOCAL	param_searc	root	FINISHED	0.5
5b34d7d098ce4399a16e8d	LOCAL	param_searc	root	FINISHED	0.5
9c58599ca05a48e28770c7	LOCAL	param_searc	root	FINISHED	0.5
c90c7a8c426d4a95b3fa75	LOCAL	param_searc	root	FINISHED	0.5
14dd269a209541ba84f013	LOCAL	param_searc	root	FINISHED	0.5

© 2021 Buda M et al. *JAMA Network Open.*

c7e33dd32430497b8a50da	LOCAL	param_searc root	FINISHED	0.5
74058123577540cd843ad2	LOCAL	param_searc root	FINISHED	0.5
432c9cddf91743ab938f983	LOCAL	param_searc root	FINISHED	0.5
cc38828ccc5a45e282b341	LOCAL	param_searc root	FINISHED	0.5
b278265a66b0475ea87eb4	LOCAL	param_searc root	FINISHED	0.5
b31cdee4434d47d48b5277	LOCAL	param_searc root	FINISHED	0.5
3f2c1dcfcec14107b16f141f	LOCAL	param_searc root	FINISHED	0.5
1487192efeeb4d558a570a	LOCAL	param_searc root	FINISHED	0.5
24adad179a7a43619b2148	LOCAL	param_searc root	FINISHED	0.5
2ee3461b62ba4d1580620	LOCAL	param_searc root	FINISHED	0.5
f3e40f13fc524b3b92b9664	LOCAL	param_searc root	FINISHED	0.5
605eb3990d124c79af98eb	LOCAL	param_searc root	FINISHED	0.5
0df0f1afb378495e8dde4ee	LOCAL	param_searc root	FINISHED	0.5
b01f71d51d0745d19235e0	LOCAL	param_searc root	FINISHED	0.5
eb9f11d5ebd243b5873f8b	LOCAL	param_searc root	FINISHED	0.5
b4eec257b8fa4ee084c2ca1	LOCAL	param_searc root	FINISHED	0.5
d7ae7b7dbdf84a39889339	LOCAL	param_searc root	FINISHED	0.5
611c3edddd544ab2aad4d8	LOCAL	param_searc root	FINISHED	0.5
85a37ee0378e4902b1b9d5	LOCAL	param_searc root	FINISHED	0.5
c89adab4f3614fbc8b1b590	LOCAL	param_searc root	FINISHED	0.5
96f980fa5bb447fbbf625bd	LOCAL	param_searc root	FINISHED	0.5
fb0620515cc940b187d762	LOCAL	param_searc root	FINISHED	0.5
5c292f4f98dd4a07a04db5	LOCAL	param_searc root	FINISHED	0.5
426a5fb92e3b4abab11fb4f	LOCAL	param_searc root	FINISHED	0.5
98d9c65e4bfd47f4a23fb8c	LOCAL	param_searc root	FINISHED	0.5
262578edb41f4a05880c13	LOCAL	param_searc root	FINISHED	0.5
5adbeb22aea34293942e21	LOCAL	param_searc root	FINISHED	0.5
bb2879e7143247499c9649	LOCAL	param_searc root	FINISHED	0.5
a4d1e62f7a6a41da97ecffc	LOCAL	param_searc root	FINISHED	0.5
b017d306910d4ab69d278	LOCAL	param_searc root	FINISHED	0.5
56fd70465b864336b94456	LOCAL	param_searc root	FINISHED	0.5
10de06c3f2d7483eb1c1a4	LOCAL	param_searc root	FINISHED	0.5
a867c25e2a4c413ab9e718	LOCAL	param_searc root	FINISHED	0.25
2d68a7f96c9342bab8ec3d	LOCAL	param_searc root	FINISHED	0.25
4b4a3cb9d1444d0ea513dc	LOCAL	param_searc root	FINISHED	0.25
b6e9936503d4456e87951	LOCAL	param_searc root	FINISHED	0.25
dbf788685fbf4d3c98b401d	LOCAL	param_searc root	FINISHED	0.25
cd8aecdf30154869a9498d	LOCAL	param_searc root	FINISHED	0.25
a2f49ca6e58848b5a4322e	LOCAL	param_searc root	FINISHED	0.25
e79c01f441734dcfbcd8d60	LOCAL	param_searc root	FINISHED	0.25
693c3c636ea345b8913c05	LOCAL	param_searc root	FINISHED	0.25

© 2021 Buda M et al. *JAMA Network Open.*

0ef87b234994438ca8d9eb	LOCAL	param_searc	root	FINISHED	0.25
7d6d315fb99644138e3e4e	LOCAL	param_searc	root	FINISHED	0.25
f4a760f95f5842edb13f24a	LOCAL	param_searc	root	FINISHED	0.25
b5b71cf9c1ff45b3bb9eebe	LOCAL	param_searc	root	FINISHED	0.25
67c2001adf4245a9bef241d	LOCAL	param_searc	root	FINISHED	0.25
e9b27c61b8394a558f8d26	LOCAL	param_searc	root	FINISHED	0.25
bb2a7c5d33224e1da95491	LOCAL	param_searc	root	FINISHED	0.25
d2c2d937e2eb405388f11a	LOCAL	param_searc	root	FINISHED	0.25
a24c389b0c6e4d15a37716	LOCAL	param_searc	root	FINISHED	0.25
71c996814e7d4032877c32	LOCAL	param_searc	root	FINISHED	0.25
35c529288fda45d189d21e	LOCAL	param_searc	root	FINISHED	0.25
cd1cdc002b4247f989a2d4	LOCAL	param_searc	root	FINISHED	0.25
7f183284a7b9454aa81f34f	LOCAL	param_searc	root	FINISHED	0.25
cb55f1d7bd9e4e8db17eec	LOCAL	param_searc	root	FINISHED	0.25
19ddd982043e44d48f9e6c	LOCAL	param_searc	root	FINISHED	0.25
dbd82d6f2aa84568a679cd	LOCAL	param_searc	root	FINISHED	0.25
859ccb85160e4c0880cb97	LOCAL	param_searc	root	FINISHED	0.25
666913b1bce844ea8a3e24	LOCAL	param_searc	root	FINISHED	0.25
c08a82784db24f7b9fe317	LOCAL	param_searc	root	FINISHED	0.25
9b1f5489e4f3496998bf07c	LOCAL	param_searc	root	FINISHED	0.25
52ec1947cb6c4171974ce2	LOCAL	param_searc	root	FINISHED	0.25
b50b747fb3784d93b03591	LOCAL	param_searc	root	FINISHED	0.25
7f448dfae6384f8ba04553f	LOCAL	param_searc	root	FINISHED	0.25
15ab6071f77a415e8c9f1f2	LOCAL	param_searc	root	FINISHED	0.25
77f65d3e1361492da51918	LOCAL	param_searc	root	FINISHED	0.25
09d2e321046148e398d80	LOCAL	param_searc	root	FINISHED	0.25
89dba114446940f78a1104	LOCAL	param_searc	root	FINISHED	0.25
86aa5cf39ce143058ccb460	LOCAL	param_searc	root	FINISHED	0.25
26fccc80bf6349ee802c4c4	LOCAL	param_searc	root	FINISHED	0.25
5834f096fe5147cb88e190f	LOCAL	param_searc	root	FINISHED	0.25
80288ae4ea754a1cbb3b0c	LOCAL	param_searc	root	FINISHED	0.25
80bfa4e34ffe44f3b08f9595	LOCAL	param_searc	root	FINISHED	0.25
01fb64ad1fb0427b870374	LOCAL	param_searc	root	FINISHED	0.25
fd153a55b16f438489e0fd3	LOCAL	param_searc	root	FINISHED	0.25
ca278fddeb1540cc9f0c005	LOCAL	param_searc	root	FINISHED	0.25
c3306d52377d4a0c953e50	LOCAL	param_searc	root	FINISHED	0.25
33d7f08162be4eed85ed51	LOCAL	param_searc	root	FINISHED	0.25
fbf992d39b6248eda976ab	LOCAL	param_searc	root	FINISHED	0.25
72a72ee43aab435cac46f5	LOCAL	param_searc	root	FINISHED	0.25
f2d31307df0547959dfcc29	LOCAL	param_searc	root	FINISHED	1
3a7b4098fb264d25a14a09	LOCAL	param_searc	root	FINISHED	1

© 2021 Buda M et al. *JAMA Network Open.*

556e9af1044949a0a0b2f0f	LOCAL	param_searc	root	FINISHED	1
573ae955cbbf48ba9ef3f8b	LOCAL	param_searc	root	FINISHED	1
54e5a80f2cd24b298221b2	LOCAL	param_searc	root	FINISHED	1
93ff2bfa03bb48fa85ea4df3	LOCAL	param_searc	root	FINISHED	1
9a69f161be674b5aacdcc5	LOCAL	param_searc	root	FINISHED	1
ed9f1ab1693a4d7ca11466	LOCAL	param_searc	root	FINISHED	1
07ba5c5770584042876a9b	LOCAL	param_searc	root	FINISHED	1
d3af12d446fa421ba69564	LOCAL	param_searc	root	FINISHED	1
b62f67189db64b8cb0ae2ff	LOCAL	param_searc	root	FINISHED	1
a560d86aae5c409c97113b	LOCAL	param_searc	root	FINISHED	1
9a8c8ecf7945400b8c0719	LOCAL	param_searc	root	FINISHED	1
c5b0022680e44bf9a6c94e	LOCAL	param_searc	root	FINISHED	1
036fbb14cada40aca44540	LOCAL	param_searc	root	FINISHED	1
332d70d1f58d4198a02d3e	LOCAL	param_searc	root	FINISHED	1
f8579be19975415ab0b537	LOCAL	param_searc	root	FINISHED	1
74038285851145c488603b	LOCAL	param_searc	root	FINISHED	1
0e7c636c49c04f8080381a	LOCAL	param_searc	root	FINISHED	1
1091914706f4478bb7b39c	LOCAL	param_searc	root	FINISHED	1
ad2f30500f224a2e8f035f9	LOCAL	param_searc	root	FINISHED	1
224f2832d625494682124b	LOCAL	param_searc	root	FINISHED	1
6f6027bc50fa4bf3a0fd555	LOCAL	param_searc	root	FINISHED	1
e16fd96453bd491db3b17f	LOCAL	param_searc	root	FINISHED	1
5d237b2253df4b6b9cf39e	LOCAL	param_searc	root	FINISHED	1
3dd5110421504e91945d2	LOCAL	param_searc	root	FINISHED	1
6b9e5b0d10364026a4d61	LOCAL	param_searc	root	FINISHED	1
a5fc9cd48de143fca155dda	LOCAL	param_searc	root	FINISHED	1
0fe431acd83b4416b4ec6d	LOCAL	param_searc	root	FINISHED	1
ad3c2cff2397418481dd58b	LOCAL	param_searc	root	FINISHED	1
51bd7ff57d6f4b18b57ade7	LOCAL	param_searc	root	FINISHED	1
e70cabaaa2874fc78a8c142	LOCAL	param_searc	root	FINISHED	1
64531823a65b4859886ba9	LOCAL	param_searc	root	FINISHED	1
80bb4cf44b8d4a73a0e112	LOCAL	param_searc	root	FINISHED	1
4d037d6ff76b473ca32341	LOCAL	param_searc	root	FINISHED	1
748780bac58644ccacef0a4	LOCAL	param_searc	root	FINISHED	1
8a068b38639d4fe5820030	LOCAL	param_searc	root	FINISHED	1
d82208342bcf4818948cfac	LOCAL	param_searc	root	FINISHED	1
071d15cc8d8f4b3db2debd	LOCAL	param_searc	root	FINISHED	1
6eb25c8071614d53b48f1a	LOCAL	param_searc	root	FINISHED	1
b32b01971511477eb57e1	LOCAL	param_searc	root	FINISHED	1
6ff60d5e6fb24d07b223ed1	LOCAL	param_searc	root	FINISHED	1
7fedcf7717d44825b2878d	LOCAL	param_searc	root	FINISHED	1

© 2021 Buda M et al. *JAMA Network Open.*

449d7b3166a44d3889e94c	LOCAL	param_searc	root	FINISHED	1
9b2b345b7ebf47849dfadc	LOCAL	param_searc	root	FINISHED	1
e2164f1b1ba940609e0621	LOCAL	param_searc	root	FINISHED	1
747646e0f2784c3db3b18f	LOCAL	param_searc	root	FINISHED	1
4ca0e848297943f6965d89	LOCAL	param_searc	root	FINISHED	1
6589f1579c604698b92a3c	LOCAL	param_searc	root	FINISHED	0.5
674b210677d148258fabcb	LOCAL	param_searc	root	FINISHED	0.5
9b202ecd293a4d88aeed3f	LOCAL	param_searc	root	FINISHED	0.5
802e853ccf5f40468c50d56	LOCAL	param_searc	root	FINISHED	0.5
dcdbef25a52048b3b6d6d2	LOCAL	param_searc	root	FINISHED	0.5
52d395b15a7d4d82a347d	LOCAL	param_searc	root	FINISHED	0.5
97aecfad039a4c4d95166a	LOCAL	param_searc	root	FINISHED	0.5
133373b835f241d5a7bc1c	LOCAL	param_searc	root	FINISHED	0.5
e2802deb509541d782ea41	LOCAL	param_searc	root	FINISHED	0.5
05942cb30952445ca8b346	LOCAL	param_searc	root	FINISHED	0.5
348eb532d9a344bc9bacdd	LOCAL	param_searc	root	FINISHED	0.5
6121f3ffa8064da59450035	LOCAL	param_searc	root	FINISHED	0.5
60238ec1014a4f3cbe9d0e	LOCAL	param_searc	root	FINISHED	0.5
ea160afb71f54bef820792f	LOCAL	param_searc	root	FINISHED	0.5
22b06b219ed04570a02d6	LOCAL	param_searc	root	FINISHED	0.5
f3dc4087e00946bdbbf377f	LOCAL	param_searc	root	FINISHED	0.5
28604b9bde854e8786599	LOCAL	param_searc	root	FINISHED	0.5
fb36964319b74367aba61d	LOCAL	param_searc	root	FINISHED	0.5
33375f95b82841e49c4376	LOCAL	param_searc	root	FINISHED	0.5
2ed1e90c380e4c1bb8734a	LOCAL	param_searc	root	FINISHED	0.5
ca22ed8074ef48be8140a0	LOCAL	param_searc	root	FINISHED	0.5
be709961ef53485fa6f47a8	LOCAL	param_searc	root	FINISHED	0.5
0b368e3bfd0c48a7961213	LOCAL	param_searc	root	FINISHED	0.5
135eaa3e4d8744a8aae5f2	LOCAL	param_searc	root	FINISHED	0.5
3abe46a58cc54170bb1cb7	LOCAL	param_searc	root	FINISHED	0.5
04fc35193c334abdaf6ca0c	LOCAL	param_searc	root	FINISHED	0.5
0a31b2a7f2764a37abe73d	LOCAL	param_searc	root	FINISHED	0.5
c0517b67ab944b93958ff9	LOCAL	param_searc	root	FINISHED	0.5
2e5d0bf0393749afa5cb3f8	LOCAL	param_searc	root	FINISHED	0.5
fb09251b475b4ff99a7ba8f	LOCAL	param_searc	root	FINISHED	0.5
fce2e77f1da549faa0c5686	LOCAL	param_searc	root	FINISHED	0.5
006c06a42bcb4c71a0deb1	LOCAL	param_searc	root	FINISHED	0.5
a14e6f27cdc244c9861a2a3	LOCAL	param_searc	root	FINISHED	0.5
3415066ad665421480a3ca	LOCAL	param_searc	root	FINISHED	0.5
b445a4e3b1da4afcb9fa903	LOCAL	param_searc	root	FINISHED	0.5
237d873b0c4940dc9c8933	LOCAL	param_searc	root	FINISHED	0.5

© 2021 Buda M et al. *JAMA Network Open.*

02b87d16bc514cebaaa306	LOCAL	param_searc	root	FINISHED	0.5
56006b313d0b47e0a4bf8d	LOCAL	param_searc	root	FINISHED	0.5
769c4e2cb76d44849761e8	LOCAL	param_searc	root	FINISHED	0.5
a07f4ffb7af449268531795	LOCAL	param_searc	root	FINISHED	0.5
3679bbef1da9409fb9e839	LOCAL	param_searc	root	FINISHED	0.5
602200ffbd654dd7b35d55	LOCAL	param_searc	root	FINISHED	0.5
032be7e0a8044998ab22b3	LOCAL	param_searc	root	FINISHED	0.5
b4cc997afca74ba28e06812	LOCAL	param_searc	root	FINISHED	0.5
b0a8077f54e54458a1f596	LOCAL	param_searc	root	FINISHED	0.5
ab3eabc86c844e5e8d044d	LOCAL	param_searc	root	FINISHED	0.5
04698c9ad5e24168844dc2	LOCAL	param_searc	root	FINISHED	0.5
1b8c446cd9f24772a71413	LOCAL	param_searc	root	FINISHED	0.5
e26f220094d9418c92b0e5	LOCAL	param_searc	root	FINISHED	0.25
1d03df4b59df49d1a2f6fae	LOCAL	param_searc	root	FINISHED	0.25
fc18c236d48f4e2cbbd3ccc	LOCAL	param_searc	root	FINISHED	0.25
e3cf565c63db4491acdb2d	LOCAL	param_searc	root	FINISHED	0.25
07671aa2b9cb445387ca35	LOCAL	param_searc	root	FINISHED	0.25
ca19cf4c5af446cdb1f2c28c	LOCAL	param_searc	root	FINISHED	0.25
046ac3f96fa04030858ffba	LOCAL	param_searc	root	FINISHED	0.25
fb782f08391449afa4c82fc0	LOCAL	param_searc	root	FINISHED	0.25
5bc32b84c59541ee96e8c9	LOCAL	param_searc	root	FINISHED	0.25
293661d6b64249ebbda52	LOCAL	param_searc	root	FINISHED	0.25
c9ebc4befe584501900796	LOCAL	param_searc	root	FINISHED	0.25
7240572eedca4aa9a3f880	LOCAL	param_searc	root	FINISHED	0.25
3d19a35ef2c144f29ebf2bc	LOCAL	param_searc	root	FINISHED	0.25
70ee96bf8df64407b6a875	LOCAL	param_searc	root	FINISHED	0.25
6f224ea40194410a91ea1e	LOCAL	param_searc	root	FINISHED	0.25
ed49117560c34058a5576e	LOCAL	param_searc	root	FINISHED	0.25
67c2a987b04c4bd3b6d0ba	LOCAL	param_searc	root	FINISHED	0.25
f16a3f67ac5e44bd8e18001	LOCAL	param_searc	root	FINISHED	0.25
1be3c74299d6447abc78f0	LOCAL	param_searc	root	FINISHED	0.25
bdf40abf1c064173b97edc8	LOCAL	param_searc	root	FINISHED	0.25
435668eb06924c51935e54	LOCAL	param_searc	root	FINISHED	0.25
d76b59a1537e42919ba298	LOCAL	param_searc	root	FINISHED	0.25
2d313c5fd8bc492bbd3d44	LOCAL	param_searc	root	FINISHED	0.25
17a831b198314d8ca59cc8	LOCAL	param_searc	root	FINISHED	0.25
55e1d51e76ca48ebaa2693	LOCAL	param_searc	root	FINISHED	0.25
ddfc01b796794eb78e8101	LOCAL	param_searc	root	FINISHED	0.25
b78e867bf8284cd793033f	LOCAL	param_searc	root	FINISHED	0.25
ed0d6ef1ff5446e3bb45469	LOCAL	param_searc	root	FINISHED	0.25
4c24ec75ef134f868f0c1b5	LOCAL	param_searc	root	FINISHED	0.25

© 2021 Buda M et al. *JAMA Network Open.*

1cd51b64f66a4966aeaa7d	LOCAL	param_searc	root	FINISHED	0.25
feb8c4e761264b2a95ef399	LOCAL	param_searc	root	FINISHED	0.25
ae6171e7dcf640ef91d1344	LOCAL	param_searc	root	FINISHED	0.25
d723f0f355744d45a0bf428	LOCAL	param_searc	root	FINISHED	0.25
26e60f44e90f4a909e9fb22	LOCAL	param_searc	root	FINISHED	0.25
76f3f18eb66a4a58ae9a8b	LOCAL	param_searc	root	FINISHED	0.25
b42938dbe95146999645f0	LOCAL	param_searc	root	FINISHED	0.25
1c3a4449b4fe4370ad5d70	LOCAL	param_searc	root	FINISHED	0.25
9c9eba27f6db4e8289f4c28	LOCAL	param_searc	root	FINISHED	0.25
8cb9a67366ce4a3d9344d0	LOCAL	param_searc	root	FINISHED	0.25
77b7e32cb7774e5aa8c025	LOCAL	param_searc	root	FINISHED	0.25
6baa640f73524f7c8dfb111	LOCAL	param_searc	root	FINISHED	0.25
88ba6d636601438f903e9c	LOCAL	param_searc	root	FINISHED	0.25
9d7b546c03fa49c5b6dbb7	LOCAL	param_searc	root	FINISHED	0.25
45db22deea6c4875b5b72a	LOCAL	param_searc	root	FINISHED	0.25
e15790eb4dae4af3a656d8	LOCAL	param_searc	root	FINISHED	0.25
e08a59b1f057461780ed8a	LOCAL	param_searc	root	FINISHED	0.25
1f41c98a18f645ef83b3c2f	LOCAL	param_searc	root	FINISHED	0.25
c1ad7c03e2834f1b8c7516	LOCAL	param_searc	root	FINISHED	0.25
64cc25498e984bbfbe5d82	LOCAL	param_searc	root	FINISHED	1
4942690d17a74f248f59f20	LOCAL	param_searc	root	FINISHED	1
9cc93165655d42caa7b3c8	LOCAL	param_searc	root	FINISHED	1
2ec70c760ad34305ad583a	LOCAL	param_searc	root	FINISHED	1
5bba4a9e54e8436c86d65f	LOCAL	param_searc	root	FINISHED	1
3c599eafa9e846f98530c42	LOCAL	param_searc	root	FINISHED	1
c3a8b747d996491fac1b2d	LOCAL	param_searc	root	FINISHED	1
12aa4c640e5e4244b36fb0	LOCAL	param_searc	root	FINISHED	1
e79f64124b7244268b7da5	LOCAL	param_searc	root	FINISHED	1
5b1234b80ff04025834a06	LOCAL	param_searc	root	FINISHED	1
6680c8c2faed441095d900	LOCAL	param_searc	root	FINISHED	1
c4e994ee5c4e4b7eba7719	LOCAL	param_searc	root	FINISHED	1
5f8d3bbd350543c5847426	LOCAL	param_searc	root	FINISHED	1
8f9e9e2dd5964e64bd441f	LOCAL	param_searc	root	FINISHED	1
60f8b7a0cd844dbbbbde41	LOCAL	param_searc	root	FINISHED	1
cb0c974484014f9ba8e9ce	LOCAL	param_searc	root	FINISHED	1
7296723e2b7e48e6a56386	LOCAL	param_searc	root	FINISHED	1
96facc844da34718baa88a	LOCAL	param_searc	root	FINISHED	1
a6734d2983934056a409ac	LOCAL	param_searc	root	FINISHED	1
c93df677ac5545e5a97dc8	LOCAL	param_searc	root	FINISHED	1
5221a2def9504629ba7997	LOCAL	param_searc	root	FINISHED	1
fd6c8b15838247e8b75741	LOCAL	param_searc	root	FINISHED	1

© 2021 Buda M et al. *JAMA Network Open.*

05c41e4f521e417583badc	LOCAL	param_searc root	FINISHED	1
3b80b5baf86c48bba72582	LOCAL	param_searc root	FINISHED	1
ec2fd0fad7fd4f239d001af9	LOCAL	param_searc root	FINISHED	1
e74e11889cf6433cacb62b	LOCAL	param_searc root	FINISHED	1
53ea59c56f064ef7bcf4b23	LOCAL	param_searc root	FINISHED	1
ae5e5983ac8b4bf78ec7e3	LOCAL	param_searc root	FINISHED	1
d780c94c37394cf3a4c0ba7	LOCAL	param_searc root	FINISHED	1
b0a95361e71546778639f9	LOCAL	param_searc root	FINISHED	1
87c6677fd6c8467ab4808f9	LOCAL	param_searc root	FINISHED	1
9353d57b14a5464b8ace69	LOCAL	param_searc root	FINISHED	1
2e2b041d14394025b5d92	LOCAL	param_searc root	FINISHED	0.5
53a2b67aa3944ebdb99668	LOCAL	param_searc root	FINISHED	0.5
34368986285e4455b319a8	LOCAL	param_searc root	FINISHED	0.5
49f3451bef7640d994312d	LOCAL	param_searc root	FINISHED	0.5
0f0474cb9da64bda97ad9b	LOCAL	param_searc root	FINISHED	0.5
b321d0ee00514ed2ae7ce3	LOCAL	param_searc root	FINISHED	0.5
2093078e2e9c4a75936a70	LOCAL	param_searc root	FINISHED	0.5
ebcfa762d4464a8ebb09ac	LOCAL	param_searc root	FINISHED	0.5
e35c1fbce71d46f096ab25e	LOCAL	param_searc root	FINISHED	0.5
0119ed6c2113453ea20951	LOCAL	param_searc root	FINISHED	0.5
4df83009202244cc8749fad	LOCAL	param_searc root	FINISHED	0.5
67de63b0048a44d383b4b	LOCAL	param_searc root	FINISHED	0.5
f8c1f221c83241289b45f80	LOCAL	param_searc root	FINISHED	0.5
e29143e280104b6cbc271a	LOCAL	param_searc root	FINISHED	0.5
8ebf4a244b1741968aaed4	LOCAL	param_searc root	FINISHED	0.5
fd487e5b01dc4a188d5522	LOCAL	param_searc root	FINISHED	0.5
ea7334f4f7504b8daac65b4	LOCAL	param_searc root	FINISHED	0.5
c1aa58af5c2c48e295baec7	LOCAL	param_searc root	FINISHED	0.5
eaf73cd2dbee4d68a2f68b	LOCAL	param_searc root	FINISHED	0.5
6060042881114794967887	LOCAL	param_searc root	FINISHED	0.5
b7f9f82e22e249479646de	LOCAL	param_searc root	FINISHED	0.5
85b36ca5fe2f4b6f9370ff05	LOCAL	param_searc root	FINISHED	0.5
224239074eff4b8eaafaf4fa	LOCAL	param_searc root	FINISHED	0.5
7569d1106de44e9f890c15	LOCAL	param_searc root	FINISHED	0.5
ed253d07f05343c5bf67a1	LOCAL	param_searc root	FINISHED	0.5
3e7f9cbd393a44b3b6d29d	LOCAL	param_searc root	FINISHED	0.5
d794ae9eea74444aa7d2ba	LOCAL	param_searc root	FINISHED	0.5
784843218d7946718ab66	LOCAL	param_searc root	FINISHED	0.5
b0e55dde96ad4ddc8ae48e	LOCAL	param_searc root	FINISHED	0.5
dbb58d53c3bd4173aeea6d	LOCAL	param_searc root	FINISHED	0.5
f62937f1d4b244858c639a	LOCAL	param_searc root	FINISHED	0.5

© 2021 Buda M et al. *JAMA Network Open.*

2b0ed2bd990d4ba5b2320	LOCAL	param_searc	root	FINISHED	0.5
5f66d8125bdd40e297d331	LOCAL	param_searc	root	FINISHED	0.25
ba8d5fedea8e4d3c892d2e	LOCAL	param_searc	root	FINISHED	0.25
cef0440a1f714ff4b964aeb	LOCAL	param_searc	root	FINISHED	0.25
766debc1db19448da95ce5	LOCAL	param_searc	root	FINISHED	0.25
989cb886dbda47fa814f70	LOCAL	param_searc	root	FINISHED	0.25
21dca7fd444e4cfbade0d25	LOCAL	param_searc	root	FINISHED	0.25
ae3ed3f286c44cdd82d7dfa	LOCAL	param_searc	root	FINISHED	0.25
bf3bcadbb64544b5add175	LOCAL	param_searc	root	FINISHED	0.25
68c4ac90a95d49c5b7c325	LOCAL	param_searc	root	FINISHED	0.25
d73383bdb76e4d5582624	LOCAL	param_searc	root	FINISHED	0.25
a6b9bffd9690489985f789c	LOCAL	param_searc	root	FINISHED	0.25
9c37afb052634f66be77dd	LOCAL	param_searc	root	FINISHED	0.25
8fecb5756d9048f8b445a4	LOCAL	param_searc	root	FINISHED	0.25
3ae267bc353d4151943919	LOCAL	param_searc	root	FINISHED	0.25
b0cf075b56994a59b2b94a	LOCAL	param_searc	root	FINISHED	0.25
9b6cf0ae99704c5ca29092	LOCAL	param_searc	root	FINISHED	0.25
6507563b5ba34a9ea7b1a6	LOCAL	param_searc	root	FINISHED	0.25
6f1d079a9b6c4a4dad8569	LOCAL	param_searc	root	FINISHED	0.25
0eb6e63ce4c240a499c019	LOCAL	param_searc	root	FINISHED	0.25
2ef3b00737804a3baf96b5	LOCAL	param_searc	root	FINISHED	0.25
aaa834e7143349c6a1706b	LOCAL	param_searc	root	FINISHED	0.25
8c02c509fcd24653a435c8a	LOCAL	param_searc	root	FINISHED	0.25
4e94af83358842479ea955	LOCAL	param_searc	root	FINISHED	0.25
9231891b48ac4f1ea620a8	LOCAL	param_searc	root	FINISHED	0.25
d1db252cd603464ab55178	LOCAL	param_searc	root	FINISHED	0.25
81fe34147b5e4534a65e3f	LOCAL	param_searc	root	FINISHED	0.25
731943b2f9ee401b953eea	LOCAL	param_searc	root	FINISHED	0.25
9f47571393d14dba96a2c2	LOCAL	param_searc	root	FINISHED	0.25
d732c822b3fd4c448de137	LOCAL	param_searc	root	FINISHED	0.25
58cf7bdb531743a2a0b811	LOCAL	param_searc	root	FINISHED	0.25
1c3bc38295ed4fde8a5f012	LOCAL	param_searc	root	FINISHED	0.25
72483f2dcf9949f5aa2d6d7	LOCAL	param_searc	root	FINISHED	0.25

© 2021 Buda M et al. *JAMA Network Open.*

block_config	bn_size	gamma	growth_rate	loss	loss_fun	network
(2, 6, 4, 12, 8	4	2	16	reduced-foca	{'alpha': 1.0, '	{'block_confi
(2, 6, 4, 12, 8	4	2	16	reduced-foca	{'alpha': 1.0, '	{'block_confi
(2, 6, 4, 12, 8	4	2	8	reduced-foca	{'alpha': 1.0, '	{'block_confi
(2, 6, 4, 12, 8	4	2	8	reduced-foca	{'alpha': 1.0, '	{'block_confi
(2, 6, 4, 12, 8	2	2	16	reduced-foca	{'alpha': 1.0, '	{'block_confi
(2, 6, 4, 12, 8	2	2	16	reduced-foca	{'alpha': 1.0, '	{'block_confi
(2, 6, 4, 12, 8	2	2	8	reduced-foca	{'alpha': 1.0, '	{'block_confi
(2, 6, 4, 12, 8	2	2	8	reduced-foca	{'alpha': 1.0, '	{'block_confi
(1, 3, 2, 6, 4)	4	2	16	reduced-foca	{'alpha': 1.0, '	{'block_confi
(1, 3, 2, 6, 4)	4	2	16	reduced-foca	{'alpha': 1.0, '	{'block_confi
(1, 3, 2, 6, 4)	4	2	8	reduced-foca	{'alpha': 1.0, '	{'block_confi
(1, 3, 2, 6, 4)	4	2	8	reduced-foca	{'alpha': 1.0, '	{'block_confi
(1, 3, 2, 6, 4)	2	2	16	reduced-foca	{'alpha': 1.0, '	{'block_confi
(1, 3, 2, 6, 4)	2	2	16	reduced-foca	{'alpha': 1.0, '	{'block_confi
(1, 3, 2, 6, 4)	2	2	8	reduced-foca	{'alpha': 1.0, '	{'block_confi
(1, 3, 2, 6, 4)	2	2	8	reduced-foca	{'alpha': 1.0, '	{'block_confi
(2, 6, 4, 12, 8	4	1	16	reduced-foca	{'alpha': 1.0, '	{'block_confi
(2, 6, 4, 12, 8	4	1	16	reduced-foca	{'alpha': 1.0, '	{'block_confi
(2, 6, 4, 12, 8	4	1	8	reduced-foca	{'alpha': 1.0, '	{'block_confi
(2, 6, 4, 12, 8	4	1	8	reduced-foca	{'alpha': 1.0, '	{'block_confi
(2, 6, 4, 12, 8	2	1	16	reduced-foca	{'alpha': 1.0, '	{'block_confi
(2, 6, 4, 12, 8	2	1	16	reduced-foca	{'alpha': 1.0, '	{'block_confi
(2, 6, 4, 12, 8	2	1	8	reduced-foca	{'alpha': 1.0, '	{'block_confi
(2, 6, 4, 12, 8	2	1	8	reduced-foca	{'alpha': 1.0, '	{'block_confi
(1, 3, 2, 6, 4)	4	1	16	reduced-foca	{'alpha': 1.0, '	{'block_confi
(1, 3, 2, 6, 4)	4	1	16	reduced-foca	{'alpha': 1.0, '	{'block_confi
(1, 3, 2, 6, 4)	4	1	8	reduced-foca	{'alpha': 1.0, '	{'block_confi
(1, 3, 2, 6, 4)	4	1	8	reduced-foca	{'alpha': 1.0, '	{'block_confi
(1, 3, 2, 6, 4)	2	1	16	reduced-foca	{'alpha': 1.0, '	{'block_confi
(1, 3, 2, 6, 4)	2	1	16	reduced-foca	{'alpha': 1.0, '	{'block_confi
(1, 3, 2, 6, 4)	2	1	8	reduced-foca	{'alpha': 1.0, '	{'block_confi
(1, 3, 2, 6, 4)	2	1	8	reduced-foca	{'alpha': 1.0, '	{'block_confi
(2, 6, 4, 12, 8	4	0.5	16	reduced-foca	{'alpha': 1.0, '	{'block_confi
(2, 6, 4, 12, 8	4	0.5	16	reduced-foca	{'alpha': 1.0, '	{'block_confi
(2, 6, 4, 12, 8	4	0.5	8	reduced-foca	{'alpha': 1.0, '	{'block_confi
(2, 6, 4, 12, 8	4	0.5	8	reduced-foca	{'alpha': 1.0, '	{'block_confi
(2, 6, 4, 12, 8	2	0.5	16	reduced-foca	{'alpha': 1.0, '	{'block_confi
(2, 6, 4, 12, 8	2	0.5	16	reduced-foca	{'alpha': 1.0, '	{'block_confi
(2, 6, 4, 12, 8	2	0.5	8	reduced-foca	{'alpha': 1.0, '	{'block_confi
(2, 6, 4, 12, 8	2	0.5	8	reduced-foca	{'alpha': 1.0, '	{'block_confi

© 2021 Buda M et al. *JAMA Network Open.*

(1, 3, 2, 6, 4)	4	0.5	16	reduced-foca {'alpha': 1.0, '{'block_confi
(1, 3, 2, 6, 4)	4	0.5	16	reduced-foca {'alpha': 1.0, '{'block_confi
(1, 3, 2, 6, 4)	4	0.5	8	reduced-foca {'alpha': 1.0, '{'block_confi
(1, 3, 2, 6, 4)	4	0.5	8	reduced-foca {'alpha': 1.0, '{'block_confi
(1, 3, 2, 6, 4)	2	0.5	16	reduced-foca {'alpha': 1.0, '{'block_confi
(1, 3, 2, 6, 4)	2	0.5	16	reduced-foca {'alpha': 1.0, '{'block_confi
(1, 3, 2, 6, 4)	2	0.5	8	reduced-foca {'alpha': 1.0, '{'block_confi
(1, 3, 2, 6, 4)	2	0.5	8	reduced-foca {'alpha': 1.0, '{'block_confi
(2, 6, 4, 12, 8	4	2	16	reduced-foca {'alpha': 0.5, '{'block_confi
(2, 6, 4, 12, 8	4	2	16	reduced-foca {'alpha': 0.5, '{'block_confi
(2, 6, 4, 12, 8	4	2	8	reduced-foca {'alpha': 0.5, '{'block_confi
(2, 6, 4, 12, 8	4	2	8	reduced-foca {'alpha': 0.5, '{'block_confi
(2, 6, 4, 12, 8	2	2	16	reduced-foca {'alpha': 0.5, '{'block_confi
(2, 6, 4, 12, 8	2	2	16	reduced-foca {'alpha': 0.5, '{'block_confi
(2, 6, 4, 12, 8	2	2	8	reduced-foca {'alpha': 0.5, '{'block_confi
(2, 6, 4, 12, 8	2	2	8	reduced-foca {'alpha': 0.5, '{'block_confi
(1, 3, 2, 6, 4)	4	2	16	reduced-foca {'alpha': 0.5, '{'block_confi
(1, 3, 2, 6, 4)	4	2	16	reduced-foca {'alpha': 0.5, '{'block_confi
(1, 3, 2, 6, 4)	4	2	8	reduced-foca {'alpha': 0.5, '{'block_confi
(1, 3, 2, 6, 4)	4	2	8	reduced-foca {'alpha': 0.5, '{'block_confi
(1, 3, 2, 6, 4)	2	2	16	reduced-foca {'alpha': 0.5, '{'block_confi
(1, 3, 2, 6, 4)	2	2	16	reduced-foca {'alpha': 0.5, '{'block_confi
(1, 3, 2, 6, 4)	2	2	8	reduced-foca {'alpha': 0.5, '{'block_confi
(1, 3, 2, 6, 4)	2	2	8	reduced-foca {'alpha': 0.5, '{'block_confi
(2, 6, 4, 12, 8	4	1	16	reduced-foca {'alpha': 0.5, '{'block_confi
(2, 6, 4, 12, 8	4	1	16	reduced-foca {'alpha': 0.5, '{'block_confi
(2, 6, 4, 12, 8	4	1	8	reduced-foca {'alpha': 0.5, '{'block_confi
(2, 6, 4, 12, 8	4	1	8	reduced-foca {'alpha': 0.5, '{'block_confi
(2, 6, 4, 12, 8	2	1	16	reduced-foca {'alpha': 0.5, '{'block_confi
(2, 6, 4, 12, 8	2	1	16	reduced-foca {'alpha': 0.5, '{'block_confi
(2, 6, 4, 12, 8	2	1	8	reduced-foca {'alpha': 0.5, '{'block_confi
(2, 6, 4, 12, 8	2	1	8	reduced-foca {'alpha': 0.5, '{'block_confi
(1, 3, 2, 6, 4)	4	1	16	reduced-foca {'alpha': 0.5, '{'block_confi
(1, 3, 2, 6, 4)	4	1	16	reduced-foca {'alpha': 0.5, '{'block_confi
(1, 3, 2, 6, 4)	4	1	8	reduced-foca {'alpha': 0.5, '{'block_confi
(1, 3, 2, 6, 4)	4	1	8	reduced-foca {'alpha': 0.5, '{'block_confi
(1, 3, 2, 6, 4)	2	1	16	reduced-foca {'alpha': 0.5, '{'block_confi
(1, 3, 2, 6, 4)	2	1	16	reduced-foca {'alpha': 0.5, '{'block_confi
(1, 3, 2, 6, 4)	2	1	8	reduced-foca {'alpha': 0.5, '{'block_confi
(1, 3, 2, 6, 4)	2	1	8	reduced-foca {'alpha': 0.5, '{'block_confi
(2, 6, 4, 12, 8	4	0.5	16	reduced-foca {'alpha': 0.5, '{'block_confi

© 2021 Buda M et al. *JAMA Network Open.*

(2, 6, 4, 12, 8	4	0.5	16	reduced-foca	{'alpha': 0.5, '	{'block_confi
(2, 6, 4, 12, 8	4	0.5	8	reduced-foca	{'alpha': 0.5, '	{'block_confi
(2, 6, 4, 12, 8	4	0.5	8	reduced-foca	{'alpha': 0.5, '	{'block_confi
(2, 6, 4, 12, 8	2	0.5	16	reduced-foca	{'alpha': 0.5, '	{'block_confi
(2, 6, 4, 12, 8	2	0.5	16	reduced-foca	{'alpha': 0.5, '	{'block_confi
(2, 6, 4, 12, 8	2	0.5	8	reduced-foca	{'alpha': 0.5, '	{'block_confi
(2, 6, 4, 12, 8	2	0.5	8	reduced-foca	{'alpha': 0.5, '	{'block_confi
(1, 3, 2, 6, 4)	4	0.5	16	reduced-foca	{'alpha': 0.5, '	{'block_confi
(1, 3, 2, 6, 4)	4	0.5	16	reduced-foca	{'alpha': 0.5, '	{'block_confi
(1, 3, 2, 6, 4)	4	0.5	8	reduced-foca	{'alpha': 0.5, '	{'block_confi
(1, 3, 2, 6, 4)	4	0.5	8	reduced-foca	{'alpha': 0.5, '	{'block_confi
(1, 3, 2, 6, 4)	2	0.5	16	reduced-foca	{'alpha': 0.5, '	{'block_confi
(1, 3, 2, 6, 4)	2	0.5	16	reduced-foca	{'alpha': 0.5, '	{'block_confi
(1, 3, 2, 6, 4)	2	0.5	8	reduced-foca	{'alpha': 0.5, '	{'block_confi
(1, 3, 2, 6, 4)	2	0.5	8	reduced-foca	{'alpha': 0.5, '	{'block_confi
(2, 6, 4, 12, 8	4	2	16	reduced-foca	{'alpha': 0.25	{'block_confi
(2, 6, 4, 12, 8	4	2	16	reduced-foca	{'alpha': 0.25	{'block_confi
(2, 6, 4, 12, 8	4	2	8	reduced-foca	{'alpha': 0.25	{'block_confi
(2, 6, 4, 12, 8	4	2	8	reduced-foca	{'alpha': 0.25	{'block_confi
(2, 6, 4, 12, 8	2	2	16	reduced-foca	{'alpha': 0.25	{'block_confi
(2, 6, 4, 12, 8	2	2	16	reduced-foca	{'alpha': 0.25	{'block_confi
(2, 6, 4, 12, 8	2	2	8	reduced-foca	{'alpha': 0.25	{'block_confi
(2, 6, 4, 12, 8	2	2	8	reduced-foca	{'alpha': 0.25	{'block_confi
(1, 3, 2, 6, 4)	4	2	16	reduced-foca	{'alpha': 0.25	{'block_confi
(1, 3, 2, 6, 4)	4	2	16	reduced-foca	{'alpha': 0.25	{'block_confi
(1, 3, 2, 6, 4)	4	2	8	reduced-foca	{'alpha': 0.25	{'block_confi
(1, 3, 2, 6, 4)	4	2	8	reduced-foca	{'alpha': 0.25	{'block_confi
(1, 3, 2, 6, 4)	2	2	16	reduced-foca	{'alpha': 0.25	{'block_confi
(1, 3, 2, 6, 4)	2	2	16	reduced-foca	{'alpha': 0.25	{'block_confi
(1, 3, 2, 6, 4)	2	2	8	reduced-foca	{'alpha': 0.25	{'block_confi
(1, 3, 2, 6, 4)	2	2	8	reduced-foca	{'alpha': 0.25	{'block_confi
(2, 6, 4, 12, 8	4	1	16	reduced-foca	{'alpha': 0.25	{'block_confi
(2, 6, 4, 12, 8	4	1	16	reduced-foca	{'alpha': 0.25	{'block_confi
(2, 6, 4, 12, 8	4	1	8	reduced-foca	{'alpha': 0.25	{'block_confi
(2, 6, 4, 12, 8	4	1	8	reduced-foca	{'alpha': 0.25	{'block_confi
(2, 6, 4, 12, 8	2	1	16	reduced-foca	{'alpha': 0.25	{'block_confi
(2, 6, 4, 12, 8	2	1	16	reduced-foca	{'alpha': 0.25	{'block_confi
(2, 6, 4, 12, 8	2	1	8	reduced-foca	{'alpha': 0.25	{'block_confi
(2, 6, 4, 12, 8	2	1	8	reduced-foca	{'alpha': 0.25	{'block_confi
(1, 3, 2, 6, 4)	4	1	16	reduced-foca	{'alpha': 0.25	{'block_confi
(1, 3, 2, 6, 4)	4	1	16	reduced-foca	{'alpha': 0.25	{'block_confi

© 2021 Buda M et al. *JAMA Network Open.*

(1, 3, 2, 6, 4)	4	1	8	reduced-foca	{'alpha': 0.25	{'block_confi
(1, 3, 2, 6, 4)	4	1	8	reduced-foca	{'alpha': 0.25	{'block_confi
(1, 3, 2, 6, 4)	2	1	16	reduced-foca	{'alpha': 0.25	{'block_confi
(1, 3, 2, 6, 4)	2	1	16	reduced-foca	{'alpha': 0.25	{'block_confi
(1, 3, 2, 6, 4)	2	1	8	reduced-foca	{'alpha': 0.25	{'block_confi
(1, 3, 2, 6, 4)	2	1	8	reduced-foca	{'alpha': 0.25	{'block_confi
(2, 6, 4, 12, 8	4	0.5	16	reduced-foca	{'alpha': 0.25	{'block_confi
(2, 6, 4, 12, 8	4	0.5	16	reduced-foca	{'alpha': 0.25	{'block_confi
(2, 6, 4, 12, 8	4	0.5	8	reduced-foca	{'alpha': 0.25	{'block_confi
(2, 6, 4, 12, 8	4	0.5	8	reduced-foca	{'alpha': 0.25	{'block_confi
(2, 6, 4, 12, 8	2	0.5	16	reduced-foca	{'alpha': 0.25	{'block_confi
(2, 6, 4, 12, 8	2	0.5	16	reduced-foca	{'alpha': 0.25	{'block_confi
(2, 6, 4, 12, 8	2	0.5	8	reduced-foca	{'alpha': 0.25	{'block_confi
(2, 6, 4, 12, 8	2	0.5	8	reduced-foca	{'alpha': 0.25	{'block_confi
(1, 3, 2, 6, 4)	4	0.5	16	reduced-foca	{'alpha': 0.25	{'block_confi
(1, 3, 2, 6, 4)	4	0.5	16	reduced-foca	{'alpha': 0.25	{'block_confi
(1, 3, 2, 6, 4)	4	0.5	8	reduced-foca	{'alpha': 0.25	{'block_confi
(1, 3, 2, 6, 4)	4	0.5	8	reduced-foca	{'alpha': 0.25	{'block_confi
(1, 3, 2, 6, 4)	2	0.5	16	reduced-foca	{'alpha': 0.25	{'block_confi
(1, 3, 2, 6, 4)	2	0.5	16	reduced-foca	{'alpha': 0.25	{'block_confi
(1, 3, 2, 6, 4)	2	0.5	8	reduced-foca	{'alpha': 0.25	{'block_confi
(1, 3, 2, 6, 4)	2	0.5	8	reduced-foca	{'alpha': 0.25	{'block_confi
(2, 6, 4, 12, 8	4	2	16	focal	{'alpha': 1.0, '	{'block_confi
(2, 6, 4, 12, 8	4	2	16	focal	{'alpha': 1.0, '	{'block_confi
(2, 6, 4, 12, 8	4	2	8	focal	{'alpha': 1.0, '	{'block_confi
(2, 6, 4, 12, 8	4	2	8	focal	{'alpha': 1.0, '	{'block_confi
(2, 6, 4, 12, 8	2	2	16	focal	{'alpha': 1.0, '	{'block_confi
(2, 6, 4, 12, 8	2	2	16	focal	{'alpha': 1.0, '	{'block_confi
(2, 6, 4, 12, 8	2	2	8	focal	{'alpha': 1.0, '	{'block_confi
(2, 6, 4, 12, 8	2	2	8	focal	{'alpha': 1.0, '	{'block_confi
(1, 3, 2, 6, 4)	4	2	16	focal	{'alpha': 1.0, '	{'block_confi
(1, 3, 2, 6, 4)	4	2	16	focal	{'alpha': 1.0, '	{'block_confi
(1, 3, 2, 6, 4)	4	2	8	focal	{'alpha': 1.0, '	{'block_confi
(1, 3, 2, 6, 4)	4	2	8	focal	{'alpha': 1.0, '	{'block_confi
(1, 3, 2, 6, 4)	2	2	16	focal	{'alpha': 1.0, '	{'block_confi
(1, 3, 2, 6, 4)	2	2	16	focal	{'alpha': 1.0, '	{'block_confi
(1, 3, 2, 6, 4)	2	2	8	focal	{'alpha': 1.0, '	{'block_confi
(1, 3, 2, 6, 4)	2	2	8	focal	{'alpha': 1.0, '	{'block_confi
(2, 6, 4, 12, 8	4	1	16	focal	{'alpha': 1.0, '	{'block_confi
(2, 6, 4, 12, 8	4	1	16	focal	{'alpha': 1.0, '	{'block_confi
(2, 6, 4, 12, 8	4	1	8	focal	{'alpha': 1.0, '	{'block_confi

© 2021 Buda M et al. *JAMA Network Open.*

(2, 6, 4, 12, 8	4	1	8	focal	{'alpha': 1.0, '{'block_confi
(2, 6, 4, 12, 8	2	1	16	focal	{'alpha': 1.0, '{'block_confi
(2, 6, 4, 12, 8	2	1	16	focal	{'alpha': 1.0, '{'block_confi
(2, 6, 4, 12, 8	2	1	8	focal	{'alpha': 1.0, '{'block_confi
(2, 6, 4, 12, 8	2	1	8	focal	{'alpha': 1.0, '{'block_confi
(1, 3, 2, 6, 4)	4	1	16	focal	{'alpha': 1.0, '{'block_confi
(1, 3, 2, 6, 4)	4	1	16	focal	{'alpha': 1.0, '{'block_confi
(1, 3, 2, 6, 4)	4	1	8	focal	{'alpha': 1.0, '{'block_confi
(1, 3, 2, 6, 4)	4	1	8	focal	{'alpha': 1.0, '{'block_confi
(1, 3, 2, 6, 4)	2	1	16	focal	{'alpha': 1.0, '{'block_confi
(1, 3, 2, 6, 4)	2	1	16	focal	{'alpha': 1.0, '{'block_confi
(1, 3, 2, 6, 4)	2	1	8	focal	{'alpha': 1.0, '{'block_confi
(1, 3, 2, 6, 4)	2	1	8	focal	{'alpha': 1.0, '{'block_confi
(2, 6, 4, 12, 8	4	0.5	16	focal	{'alpha': 1.0, '{'block_confi
(2, 6, 4, 12, 8	4	0.5	16	focal	{'alpha': 1.0, '{'block_confi
(2, 6, 4, 12, 8	4	0.5	8	focal	{'alpha': 1.0, '{'block_confi
(2, 6, 4, 12, 8	4	0.5	8	focal	{'alpha': 1.0, '{'block_confi
(2, 6, 4, 12, 8	2	0.5	16	focal	{'alpha': 1.0, '{'block_confi
(2, 6, 4, 12, 8	2	0.5	16	focal	{'alpha': 1.0, '{'block_confi
(2, 6, 4, 12, 8	2	0.5	8	focal	{'alpha': 1.0, '{'block_confi
(2, 6, 4, 12, 8	2	0.5	8	focal	{'alpha': 1.0, '{'block_confi
(1, 3, 2, 6, 4)	4	0.5	16	focal	{'alpha': 1.0, '{'block_confi
(1, 3, 2, 6, 4)	4	0.5	16	focal	{'alpha': 1.0, '{'block_confi
(1, 3, 2, 6, 4)	4	0.5	8	focal	{'alpha': 1.0, '{'block_confi
(1, 3, 2, 6, 4)	4	0.5	8	focal	{'alpha': 1.0, '{'block_confi
(1, 3, 2, 6, 4)	2	0.5	16	focal	{'alpha': 1.0, '{'block_confi
(1, 3, 2, 6, 4)	2	0.5	16	focal	{'alpha': 1.0, '{'block_confi
(1, 3, 2, 6, 4)	2	0.5	8	focal	{'alpha': 1.0, '{'block_confi
(1, 3, 2, 6, 4)	2	0.5	8	focal	{'alpha': 1.0, '{'block_confi
(2, 6, 4, 12, 8	4	2	16	focal	{'alpha': 0.5, '{'block_confi
(2, 6, 4, 12, 8	4	2	16	focal	{'alpha': 0.5, '{'block_confi
(2, 6, 4, 12, 8	4	2	8	focal	{'alpha': 0.5, '{'block_confi
(2, 6, 4, 12, 8	4	2	8	focal	{'alpha': 0.5, '{'block_confi
(2, 6, 4, 12, 8	2	2	16	focal	{'alpha': 0.5, '{'block_confi
(2, 6, 4, 12, 8	2	2	16	focal	{'alpha': 0.5, '{'block_confi
(2, 6, 4, 12, 8	2	2	8	focal	{'alpha': 0.5, '{'block_confi
(2, 6, 4, 12, 8	2	2	8	focal	{'alpha': 0.5, '{'block_confi
(1, 3, 2, 6, 4)	4	2	16	focal	{'alpha': 0.5, '{'block_confi
(1, 3, 2, 6, 4)	4	2	16	focal	{'alpha': 0.5, '{'block_confi
(1, 3, 2, 6, 4)	4	2	8	focal	{'alpha': 0.5, '{'block_confi
(1, 3, 2, 6, 4)	4	2	8	focal	{'alpha': 0.5, '{'block_confi

© 2021 Buda M et al. *JAMA Network Open.*

(1, 3, 2, 6, 4)	2	2	16	focal	{'alpha': 0.5, '{'block_confi
(1, 3, 2, 6, 4)	2	2	16	focal	{'alpha': 0.5, '{'block_confi
(1, 3, 2, 6, 4)	2	2	8	focal	{'alpha': 0.5, '{'block_confi
(1, 3, 2, 6, 4)	2	2	8	focal	{'alpha': 0.5, '{'block_confi
(2, 6, 4, 12, 8	4	1	16	focal	{'alpha': 0.5, '{'block_confi
(2, 6, 4, 12, 8	4	1	16	focal	{'alpha': 0.5, '{'block_confi
(2, 6, 4, 12, 8	4	1	8	focal	{'alpha': 0.5, '{'block_confi
(2, 6, 4, 12, 8	4	1	8	focal	{'alpha': 0.5, '{'block_confi
(2, 6, 4, 12, 8	2	1	16	focal	{'alpha': 0.5, '{'block_confi
(2, 6, 4, 12, 8	2	1	16	focal	{'alpha': 0.5, '{'block_confi
(2, 6, 4, 12, 8	2	1	8	focal	{'alpha': 0.5, '{'block_confi
(2, 6, 4, 12, 8	2	1	8	focal	{'alpha': 0.5, '{'block_confi
(1, 3, 2, 6, 4)	4	1	16	focal	{'alpha': 0.5, '{'block_confi
(1, 3, 2, 6, 4)	4	1	16	focal	{'alpha': 0.5, '{'block_confi
(1, 3, 2, 6, 4)	4	1	8	focal	{'alpha': 0.5, '{'block_confi
(1, 3, 2, 6, 4)	4	1	8	focal	{'alpha': 0.5, '{'block_confi
(1, 3, 2, 6, 4)	2	1	16	focal	{'alpha': 0.5, '{'block_confi
(1, 3, 2, 6, 4)	2	1	16	focal	{'alpha': 0.5, '{'block_confi
(1, 3, 2, 6, 4)	2	1	8	focal	{'alpha': 0.5, '{'block_confi
(1, 3, 2, 6, 4)	2	1	8	focal	{'alpha': 0.5, '{'block_confi
(2, 6, 4, 12, 8	4	0.5	16	focal	{'alpha': 0.5, '{'block_confi
(2, 6, 4, 12, 8	4	0.5	16	focal	{'alpha': 0.5, '{'block_confi
(2, 6, 4, 12, 8	4	0.5	8	focal	{'alpha': 0.5, '{'block_confi
(2, 6, 4, 12, 8	4	0.5	8	focal	{'alpha': 0.5, '{'block_confi
(2, 6, 4, 12, 8	2	0.5	16	focal	{'alpha': 0.5, '{'block_confi
(2, 6, 4, 12, 8	2	0.5	16	focal	{'alpha': 0.5, '{'block_confi
(2, 6, 4, 12, 8	2	0.5	8	focal	{'alpha': 0.5, '{'block_confi
(2, 6, 4, 12, 8	2	0.5	8	focal	{'alpha': 0.5, '{'block_confi
(1, 3, 2, 6, 4)	4	0.5	16	focal	{'alpha': 0.5, '{'block_confi
(1, 3, 2, 6, 4)	4	0.5	16	focal	{'alpha': 0.5, '{'block_confi
(1, 3, 2, 6, 4)	4	0.5	8	focal	{'alpha': 0.5, '{'block_confi
(1, 3, 2, 6, 4)	4	0.5	8	focal	{'alpha': 0.5, '{'block_confi
(1, 3, 2, 6, 4)	2	0.5	16	focal	{'alpha': 0.5, '{'block_confi
(1, 3, 2, 6, 4)	2	0.5	16	focal	{'alpha': 0.5, '{'block_confi
(1, 3, 2, 6, 4)	2	0.5	8	focal	{'alpha': 0.5, '{'block_confi
(1, 3, 2, 6, 4)	2	0.5	8	focal	{'alpha': 0.5, '{'block_confi
(2, 6, 4, 12, 8	4	2	16	focal	{'alpha': 0.25	{'block_confi
(2, 6, 4, 12, 8	4	2	16	focal	{'alpha': 0.25	{'block_confi
(2, 6, 4, 12, 8	4	2	8	focal	{'alpha': 0.25	{'block_confi
(2, 6, 4, 12, 8	4	2	8	focal	{'alpha': 0.25	{'block_confi
(2, 6, 4, 12, 8	2	2	16	focal	{'alpha': 0.25	{'block_confi

© 2021 Buda M et al. *JAMA Network Open.*

(2, 6, 4, 12, 8	2	2	16	focal	{'alpha': 0.25	{'block_confi
(2, 6, 4, 12, 8	2	2	8	focal	{'alpha': 0.25	{'block_confi
(2, 6, 4, 12, 8	2	2	8	focal	{'alpha': 0.25	{'block_confi
(1, 3, 2, 6, 4)	4	2	16	focal	{'alpha': 0.25	{'block_confi
(1, 3, 2, 6, 4)	4	2	16	focal	{'alpha': 0.25	{'block_confi
(1, 3, 2, 6, 4)	4	2	8	focal	{'alpha': 0.25	{'block_confi
(1, 3, 2, 6, 4)	4	2	8	focal	{'alpha': 0.25	{'block_confi
(1, 3, 2, 6, 4)	2	2	16	focal	{'alpha': 0.25	{'block_confi
(1, 3, 2, 6, 4)	2	2	16	focal	{'alpha': 0.25	{'block_confi
(1, 3, 2, 6, 4)	2	2	8	focal	{'alpha': 0.25	{'block_confi
(1, 3, 2, 6, 4)	2	2	8	focal	{'alpha': 0.25	{'block_confi
(2, 6, 4, 12, 8	4	1	16	focal	{'alpha': 0.25	{'block_confi
(2, 6, 4, 12, 8	4	1	16	focal	{'alpha': 0.25	{'block_confi
(2, 6, 4, 12, 8	4	1	8	focal	{'alpha': 0.25	{'block_confi
(2, 6, 4, 12, 8	4	1	8	focal	{'alpha': 0.25	{'block_confi
(2, 6, 4, 12, 8	2	1	16	focal	{'alpha': 0.25	{'block_confi
(2, 6, 4, 12, 8	2	1	16	focal	{'alpha': 0.25	{'block_confi
(2, 6, 4, 12, 8	2	1	8	focal	{'alpha': 0.25	{'block_confi
(2, 6, 4, 12, 8	2	1	8	focal	{'alpha': 0.25	{'block_confi
(1, 3, 2, 6, 4)	4	1	16	focal	{'alpha': 0.25	{'block_confi
(1, 3, 2, 6, 4)	4	1	16	focal	{'alpha': 0.25	{'block_confi
(1, 3, 2, 6, 4)	4	1	8	focal	{'alpha': 0.25	{'block_confi
(1, 3, 2, 6, 4)	4	1	8	focal	{'alpha': 0.25	{'block_confi
(1, 3, 2, 6, 4)	2	1	16	focal	{'alpha': 0.25	{'block_confi
(1, 3, 2, 6, 4)	2	1	16	focal	{'alpha': 0.25	{'block_confi
(1, 3, 2, 6, 4)	2	1	8	focal	{'alpha': 0.25	{'block_confi
(1, 3, 2, 6, 4)	2	1	8	focal	{'alpha': 0.25	{'block_confi
(2, 6, 4, 12, 8	4	0.5	16	focal	{'alpha': 0.25	{'block_confi
(2, 6, 4, 12, 8	4	0.5	16	focal	{'alpha': 0.25	{'block_confi
(2, 6, 4, 12, 8	4	0.5	8	focal	{'alpha': 0.25	{'block_confi
(2, 6, 4, 12, 8	4	0.5	8	focal	{'alpha': 0.25	{'block_confi
(2, 6, 4, 12, 8	2	0.5	16	focal	{'alpha': 0.25	{'block_confi
(2, 6, 4, 12, 8	2	0.5	16	focal	{'alpha': 0.25	{'block_confi
(2, 6, 4, 12, 8	2	0.5	8	focal	{'alpha': 0.25	{'block_confi
(2, 6, 4, 12, 8	2	0.5	8	focal	{'alpha': 0.25	{'block_confi
(1, 3, 2, 6, 4)	4	0.5	16	focal	{'alpha': 0.25	{'block_confi
(1, 3, 2, 6, 4)	4	0.5	16	focal	{'alpha': 0.25	{'block_confi
(1, 3, 2, 6, 4)	4	0.5	8	focal	{'alpha': 0.25	{'block_confi
(1, 3, 2, 6, 4)	4	0.5	8	focal	{'alpha': 0.25	{'block_confi
(1, 3, 2, 6, 4)	2	0.5	16	focal	{'alpha': 0.25	{'block_confi
(1, 3, 2, 6, 4)	2	0.5	16	focal	{'alpha': 0.25	{'block_confi

© 2021 Buda M et al. *JAMA Network Open.*

(1, 3, 2, 6, 4)	2	0.5	8	focal	{'alpha': 0.25	{'block_confi
(1, 3, 2, 6, 4)	2	0.5	8	focal	{'alpha': 0.25	{'block_confi
(2, 6, 4, 12, 8	4		16	weighted-CE	{'alpha': 1.0, '	{'block_confi
(2, 6, 4, 12, 8	4		16	weighted-CE	{'alpha': 1.0, '	{'block_confi
(2, 6, 4, 12, 8	4		8	weighted-CE	{'alpha': 1.0, '	{'block_confi
(2, 6, 4, 12, 8	4		8	weighted-CE	{'alpha': 1.0, '	{'block_confi
(2, 6, 4, 12, 8	2		16	weighted-CE	{'alpha': 1.0, '	{'block_confi
(2, 6, 4, 12, 8	2		16	weighted-CE	{'alpha': 1.0, '	{'block_confi
(2, 6, 4, 12, 8	2		8	weighted-CE	{'alpha': 1.0, '	{'block_confi
(2, 6, 4, 12, 8	2		8	weighted-CE	{'alpha': 1.0, '	{'block_confi
(1, 3, 2, 6, 4)	4		16	weighted-CE	{'alpha': 1.0, '	{'block_confi
(1, 3, 2, 6, 4)	4		16	weighted-CE	{'alpha': 1.0, '	{'block_confi
(1, 3, 2, 6, 4)	4		8	weighted-CE	{'alpha': 1.0, '	{'block_confi
(1, 3, 2, 6, 4)	4		8	weighted-CE	{'alpha': 1.0, '	{'block_confi
(1, 3, 2, 6, 4)	2		16	weighted-CE	{'alpha': 1.0, '	{'block_confi
(1, 3, 2, 6, 4)	2		16	weighted-CE	{'alpha': 1.0, '	{'block_confi
(1, 3, 2, 6, 4)	2		8	weighted-CE	{'alpha': 1.0, '	{'block_confi
(1, 3, 2, 6, 4)	2		8	weighted-CE	{'alpha': 1.0, '	{'block_confi
(2, 6, 4, 12, 8	4		16	CE	{'alpha': 1.0, '	{'block_confi
(2, 6, 4, 12, 8	4		16	CE	{'alpha': 1.0, '	{'block_confi
(2, 6, 4, 12, 8	4		8	CE	{'alpha': 1.0, '	{'block_confi
(2, 6, 4, 12, 8	4		8	CE	{'alpha': 1.0, '	{'block_confi
(2, 6, 4, 12, 8	2		16	CE	{'alpha': 1.0, '	{'block_confi
(2, 6, 4, 12, 8	2		16	CE	{'alpha': 1.0, '	{'block_confi
(2, 6, 4, 12, 8	2		8	CE	{'alpha': 1.0, '	{'block_confi
(2, 6, 4, 12, 8	2		8	CE	{'alpha': 1.0, '	{'block_confi
(1, 3, 2, 6, 4)	4		16	CE	{'alpha': 1.0, '	{'block_confi
(1, 3, 2, 6, 4)	4		16	CE	{'alpha': 1.0, '	{'block_confi
(1, 3, 2, 6, 4)	4		8	CE	{'alpha': 1.0, '	{'block_confi
(1, 3, 2, 6, 4)	4		8	CE	{'alpha': 1.0, '	{'block_confi
(1, 3, 2, 6, 4)	2		16	CE	{'alpha': 1.0, '	{'block_confi
(1, 3, 2, 6, 4)	2		16	CE	{'alpha': 1.0, '	{'block_confi
(1, 3, 2, 6, 4)	2		8	CE	{'alpha': 1.0, '	{'block_confi
(1, 3, 2, 6, 4)	2		8	CE	{'alpha': 1.0, '	{'block_confi
(2, 6, 4, 12, 8	4		16	weighted-CE	{'alpha': 0.5, '	{'block_confi
(2, 6, 4, 12, 8	4		16	weighted-CE	{'alpha': 0.5, '	{'block_confi
(2, 6, 4, 12, 8	4		8	weighted-CE	{'alpha': 0.5, '	{'block_confi
(2, 6, 4, 12, 8	4		8	weighted-CE	{'alpha': 0.5, '	{'block_confi
(2, 6, 4, 12, 8	2		16	weighted-CE	{'alpha': 0.5, '	{'block_confi
(2, 6, 4, 12, 8	2		16	weighted-CE	{'alpha': 0.5, '	{'block_confi
(2, 6, 4, 12, 8	2		8	weighted-CE	{'alpha': 0.5, '	{'block_confi

© 2021 Buda M et al. *JAMA Network Open.*

(2, 6, 4, 12, 8	2	8	weighted-CE	{'alpha': 0.5, '{'block_confi
(1, 3, 2, 6, 4)	4	16	weighted-CE	{'alpha': 0.5, '{'block_confi
(1, 3, 2, 6, 4)	4	16	weighted-CE	{'alpha': 0.5, '{'block_confi
(1, 3, 2, 6, 4)	4	8	weighted-CE	{'alpha': 0.5, '{'block_confi
(1, 3, 2, 6, 4)	4	8	weighted-CE	{'alpha': 0.5, '{'block_confi
(1, 3, 2, 6, 4)	2	16	weighted-CE	{'alpha': 0.5, '{'block_confi
(1, 3, 2, 6, 4)	2	16	weighted-CE	{'alpha': 0.5, '{'block_confi
(1, 3, 2, 6, 4)	2	8	weighted-CE	{'alpha': 0.5, '{'block_confi
(1, 3, 2, 6, 4)	2	8	weighted-CE	{'alpha': 0.5, '{'block_confi
(2, 6, 4, 12, 8	4	16	CE	{'alpha': 0.5, '{'block_confi
(2, 6, 4, 12, 8	4	16	CE	{'alpha': 0.5, '{'block_confi
(2, 6, 4, 12, 8	4	8	CE	{'alpha': 0.5, '{'block_confi
(2, 6, 4, 12, 8	4	8	CE	{'alpha': 0.5, '{'block_confi
(2, 6, 4, 12, 8	2	16	CE	{'alpha': 0.5, '{'block_confi
(2, 6, 4, 12, 8	2	16	CE	{'alpha': 0.5, '{'block_confi
(2, 6, 4, 12, 8	2	8	CE	{'alpha': 0.5, '{'block_confi
(2, 6, 4, 12, 8	2	8	CE	{'alpha': 0.5, '{'block_confi
(1, 3, 2, 6, 4)	4	16	CE	{'alpha': 0.5, '{'block_confi
(1, 3, 2, 6, 4)	4	16	CE	{'alpha': 0.5, '{'block_confi
(1, 3, 2, 6, 4)	4	8	CE	{'alpha': 0.5, '{'block_confi
(1, 3, 2, 6, 4)	4	8	CE	{'alpha': 0.5, '{'block_confi
(1, 3, 2, 6, 4)	2	16	CE	{'alpha': 0.5, '{'block_confi
(1, 3, 2, 6, 4)	2	16	CE	{'alpha': 0.5, '{'block_confi
(1, 3, 2, 6, 4)	2	8	CE	{'alpha': 0.5, '{'block_confi
(1, 3, 2, 6, 4)	2	8	CE	{'alpha': 0.5, '{'block_confi
(2, 6, 4, 12, 8	4	16	weighted-CE	{'alpha': 0.25 {'block_confi
(2, 6, 4, 12, 8	4	16	weighted-CE	{'alpha': 0.25 {'block_confi
(2, 6, 4, 12, 8	4	8	weighted-CE	{'alpha': 0.25 {'block_confi
(2, 6, 4, 12, 8	4	8	weighted-CE	{'alpha': 0.25 {'block_confi
(2, 6, 4, 12, 8	2	16	weighted-CE	{'alpha': 0.25 {'block_confi
(2, 6, 4, 12, 8	2	16	weighted-CE	{'alpha': 0.25 {'block_confi
(2, 6, 4, 12, 8	2	8	weighted-CE	{'alpha': 0.25 {'block_confi
(2, 6, 4, 12, 8	2	8	weighted-CE	{'alpha': 0.25 {'block_confi
(1, 3, 2, 6, 4)	4	16	weighted-CE	{'alpha': 0.25 {'block_confi
(1, 3, 2, 6, 4)	4	16	weighted-CE	{'alpha': 0.25 {'block_confi
(1, 3, 2, 6, 4)	4	8	weighted-CE	{'alpha': 0.25 {'block_confi
(1, 3, 2, 6, 4)	4	8	weighted-CE	{'alpha': 0.25 {'block_confi
(1, 3, 2, 6, 4)	2	16	weighted-CE	{'alpha': 0.25 {'block_confi
(1, 3, 2, 6, 4)	2	16	weighted-CE	{'alpha': 0.25 {'block_confi
(1, 3, 2, 6, 4)	2	8	weighted-CE	{'alpha': 0.25 {'block_confi
(1, 3, 2, 6, 4)	2	8	weighted-CE	{'alpha': 0.25 {'block_confi

© 2021 Buda M et al. *JAMA Network Open.*

(2, 6, 4, 12, 8	4		16	CE	{'alpha': 0.25	{'block_confi
(2, 6, 4, 12, 8	4		16	CE	{'alpha': 0.25	{'block_confi
(2, 6, 4, 12, 8	4		8	CE	{'alpha': 0.25	{'block_confi
(2, 6, 4, 12, 8	4		8	CE	{'alpha': 0.25	{'block_confi
(2, 6, 4, 12, 8	2		16	CE	{'alpha': 0.25	{'block_confi
(2, 6, 4, 12, 8	2		16	CE	{'alpha': 0.25	{'block_confi
(2, 6, 4, 12, 8	2		8	CE	{'alpha': 0.25	{'block_confi
(2, 6, 4, 12, 8	2		8	CE	{'alpha': 0.25	{'block_confi
(1, 3, 2, 6, 4)	4		16	CE	{'alpha': 0.25	{'block_confi
(1, 3, 2, 6, 4)	4		16	CE	{'alpha': 0.25	{'block_confi
(1, 3, 2, 6, 4)	4		8	CE	{'alpha': 0.25	{'block_confi
(1, 3, 2, 6, 4)	4		8	CE	{'alpha': 0.25	{'block_confi
(1, 3, 2, 6, 4)	2		16	CE	{'alpha': 0.25	{'block_confi
(1, 3, 2, 6, 4)	2		16	CE	{'alpha': 0.25	{'block_confi
(1, 3, 2, 6, 4)	2		8	CE	{'alpha': 0.25	{'block_confi
(1, 3, 2, 6, 4)	2		8	CE	{'alpha': 0.25	{'block_confi
(2, 6, 4, 12, 8	4	2	16	reduced-foca	{'alpha': 1.0, '	{'block_confi
(2, 6, 4, 12, 8	4	2	16	reduced-foca	{'alpha': 1.0, '	{'block_confi
(2, 6, 4, 12, 8	4	2	8	reduced-foca	{'alpha': 1.0, '	{'block_confi
(2, 6, 4, 12, 8	4	2	8	reduced-foca	{'alpha': 1.0, '	{'block_confi
(2, 6, 4, 12, 8	2	2	16	reduced-foca	{'alpha': 1.0, '	{'block_confi
(2, 6, 4, 12, 8	2	2	16	reduced-foca	{'alpha': 1.0, '	{'block_confi
(2, 6, 4, 12, 8	2	2	8	reduced-foca	{'alpha': 1.0, '	{'block_confi
(2, 6, 4, 12, 8	2	2	8	reduced-foca	{'alpha': 1.0, '	{'block_confi
(1, 3, 2, 6, 4)	4	2	16	reduced-foca	{'alpha': 1.0, '	{'block_confi
(1, 3, 2, 6, 4)	4	2	16	reduced-foca	{'alpha': 1.0, '	{'block_confi
(1, 3, 2, 6, 4)	4	2	8	reduced-foca	{'alpha': 1.0, '	{'block_confi
(1, 3, 2, 6, 4)	4	2	8	reduced-foca	{'alpha': 1.0, '	{'block_confi
(1, 3, 2, 6, 4)	2	2	16	reduced-foca	{'alpha': 1.0, '	{'block_confi
(1, 3, 2, 6, 4)	2	2	16	reduced-foca	{'alpha': 1.0, '	{'block_confi
(1, 3, 2, 6, 4)	2	2	8	reduced-foca	{'alpha': 1.0, '	{'block_confi
(1, 3, 2, 6, 4)	2	2	8	reduced-foca	{'alpha': 1.0, '	{'block_confi
(2, 6, 4, 12, 8	4	1	16	reduced-foca	{'alpha': 1.0, '	{'block_confi
(2, 6, 4, 12, 8	4	1	16	reduced-foca	{'alpha': 1.0, '	{'block_confi
(2, 6, 4, 12, 8	4	1	8	reduced-foca	{'alpha': 1.0, '	{'block_confi
(2, 6, 4, 12, 8	4	1	8	reduced-foca	{'alpha': 1.0, '	{'block_confi
(2, 6, 4, 12, 8	2	1	16	reduced-foca	{'alpha': 1.0, '	{'block_confi
(2, 6, 4, 12, 8	2	1	16	reduced-foca	{'alpha': 1.0, '	{'block_confi
(2, 6, 4, 12, 8	2	1	8	reduced-foca	{'alpha': 1.0, '	{'block_confi
(2, 6, 4, 12, 8	2	1	8	reduced-foca	{'alpha': 1.0, '	{'block_confi
(1, 3, 2, 6, 4)	4	1	16	reduced-foca	{'alpha': 1.0, '	{'block_confi

© 2021 Buda M et al. *JAMA Network Open.*

(1, 3, 2, 6, 4)	4	1	16	reduced-foca	{'alpha': 1.0, '	{'block_confi
(1, 3, 2, 6, 4)	4	1	8	reduced-foca	{'alpha': 1.0, '	{'block_confi
(1, 3, 2, 6, 4)	4	1	8	reduced-foca	{'alpha': 1.0, '	{'block_confi
(1, 3, 2, 6, 4)	2	1	16	reduced-foca	{'alpha': 1.0, '	{'block_confi
(1, 3, 2, 6, 4)	2	1	16	reduced-foca	{'alpha': 1.0, '	{'block_confi
(1, 3, 2, 6, 4)	2	1	8	reduced-foca	{'alpha': 1.0, '	{'block_confi
(1, 3, 2, 6, 4)	2	1	8	reduced-foca	{'alpha': 1.0, '	{'block_confi
(2, 6, 4, 12, 8	4	0.5	16	reduced-foca	{'alpha': 1.0, '	{'block_confi
(2, 6, 4, 12, 8	4	0.5	16	reduced-foca	{'alpha': 1.0, '	{'block_confi
(2, 6, 4, 12, 8	4	0.5	8	reduced-foca	{'alpha': 1.0, '	{'block_confi
(2, 6, 4, 12, 8	4	0.5	8	reduced-foca	{'alpha': 1.0, '	{'block_confi
(2, 6, 4, 12, 8	2	0.5	16	reduced-foca	{'alpha': 1.0, '	{'block_confi
(2, 6, 4, 12, 8	2	0.5	16	reduced-foca	{'alpha': 1.0, '	{'block_confi
(2, 6, 4, 12, 8	2	0.5	8	reduced-foca	{'alpha': 1.0, '	{'block_confi
(2, 6, 4, 12, 8	2	0.5	8	reduced-foca	{'alpha': 1.0, '	{'block_confi
(1, 3, 2, 6, 4)	4	0.5	16	reduced-foca	{'alpha': 1.0, '	{'block_confi
(1, 3, 2, 6, 4)	4	0.5	16	reduced-foca	{'alpha': 1.0, '	{'block_confi
(1, 3, 2, 6, 4)	4	0.5	8	reduced-foca	{'alpha': 1.0, '	{'block_confi
(1, 3, 2, 6, 4)	4	0.5	8	reduced-foca	{'alpha': 1.0, '	{'block_confi
(1, 3, 2, 6, 4)	2	0.5	16	reduced-foca	{'alpha': 1.0, '	{'block_confi
(1, 3, 2, 6, 4)	2	0.5	16	reduced-foca	{'alpha': 1.0, '	{'block_confi
(1, 3, 2, 6, 4)	2	0.5	8	reduced-foca	{'alpha': 1.0, '	{'block_confi
(1, 3, 2, 6, 4)	2	0.5	8	reduced-foca	{'alpha': 1.0, '	{'block_confi
(2, 6, 4, 12, 8	4	2	16	reduced-foca	{'alpha': 0.5, '	{'block_confi
(2, 6, 4, 12, 8	4	2	16	reduced-foca	{'alpha': 0.5, '	{'block_confi
(2, 6, 4, 12, 8	4	2	8	reduced-foca	{'alpha': 0.5, '	{'block_confi
(2, 6, 4, 12, 8	4	2	8	reduced-foca	{'alpha': 0.5, '	{'block_confi
(2, 6, 4, 12, 8	2	2	16	reduced-foca	{'alpha': 0.5, '	{'block_confi
(2, 6, 4, 12, 8	2	2	16	reduced-foca	{'alpha': 0.5, '	{'block_confi
(2, 6, 4, 12, 8	2	2	8	reduced-foca	{'alpha': 0.5, '	{'block_confi
(2, 6, 4, 12, 8	2	2	8	reduced-foca	{'alpha': 0.5, '	{'block_confi
(1, 3, 2, 6, 4)	4	2	16	reduced-foca	{'alpha': 0.5, '	{'block_confi
(1, 3, 2, 6, 4)	4	2	16	reduced-foca	{'alpha': 0.5, '	{'block_confi
(1, 3, 2, 6, 4)	4	2	8	reduced-foca	{'alpha': 0.5, '	{'block_confi
(1, 3, 2, 6, 4)	4	2	8	reduced-foca	{'alpha': 0.5, '	{'block_confi
(1, 3, 2, 6, 4)	2	2	16	reduced-foca	{'alpha': 0.5, '	{'block_confi
(1, 3, 2, 6, 4)	2	2	16	reduced-foca	{'alpha': 0.5, '	{'block_confi
(1, 3, 2, 6, 4)	2	2	8	reduced-foca	{'alpha': 0.5, '	{'block_confi
(1, 3, 2, 6, 4)	2	2	8	reduced-foca	{'alpha': 0.5, '	{'block_confi
(2, 6, 4, 12, 8	4	1	16	reduced-foca	{'alpha': 0.5, '	{'block_confi
(2, 6, 4, 12, 8	4	1	16	reduced-foca	{'alpha': 0.5, '	{'block_confi

© 2021 Buda M et al. *JAMA Network Open.*

(2, 6, 4, 12, 8	4	1	8	reduced-foca	{'alpha': 0.5, '	{'block_confi
(2, 6, 4, 12, 8	4	1	8	reduced-foca	{'alpha': 0.5, '	{'block_confi
(2, 6, 4, 12, 8	2	1	16	reduced-foca	{'alpha': 0.5, '	{'block_confi
(2, 6, 4, 12, 8	2	1	16	reduced-foca	{'alpha': 0.5, '	{'block_confi
(2, 6, 4, 12, 8	2	1	8	reduced-foca	{'alpha': 0.5, '	{'block_confi
(2, 6, 4, 12, 8	2	1	8	reduced-foca	{'alpha': 0.5, '	{'block_confi
(1, 3, 2, 6, 4)	4	1	16	reduced-foca	{'alpha': 0.5, '	{'block_confi
(1, 3, 2, 6, 4)	4	1	16	reduced-foca	{'alpha': 0.5, '	{'block_confi
(1, 3, 2, 6, 4)	4	1	8	reduced-foca	{'alpha': 0.5, '	{'block_confi
(1, 3, 2, 6, 4)	4	1	8	reduced-foca	{'alpha': 0.5, '	{'block_confi
(1, 3, 2, 6, 4)	2	1	16	reduced-foca	{'alpha': 0.5, '	{'block_confi
(1, 3, 2, 6, 4)	2	1	16	reduced-foca	{'alpha': 0.5, '	{'block_confi
(1, 3, 2, 6, 4)	2	1	8	reduced-foca	{'alpha': 0.5, '	{'block_confi
(1, 3, 2, 6, 4)	2	1	8	reduced-foca	{'alpha': 0.5, '	{'block_confi
(2, 6, 4, 12, 8	4	0.5	16	reduced-foca	{'alpha': 0.5, '	{'block_confi
(2, 6, 4, 12, 8	4	0.5	16	reduced-foca	{'alpha': 0.5, '	{'block_confi
(2, 6, 4, 12, 8	4	0.5	8	reduced-foca	{'alpha': 0.5, '	{'block_confi
(2, 6, 4, 12, 8	4	0.5	8	reduced-foca	{'alpha': 0.5, '	{'block_confi
(2, 6, 4, 12, 8	2	0.5	16	reduced-foca	{'alpha': 0.5, '	{'block_confi
(2, 6, 4, 12, 8	2	0.5	16	reduced-foca	{'alpha': 0.5, '	{'block_confi
(2, 6, 4, 12, 8	2	0.5	8	reduced-foca	{'alpha': 0.5, '	{'block_confi
(2, 6, 4, 12, 8	2	0.5	8	reduced-foca	{'alpha': 0.5, '	{'block_confi
(1, 3, 2, 6, 4)	4	0.5	16	reduced-foca	{'alpha': 0.5, '	{'block_confi
(1, 3, 2, 6, 4)	4	0.5	16	reduced-foca	{'alpha': 0.5, '	{'block_confi
(1, 3, 2, 6, 4)	4	0.5	8	reduced-foca	{'alpha': 0.5, '	{'block_confi
(1, 3, 2, 6, 4)	4	0.5	8	reduced-foca	{'alpha': 0.5, '	{'block_confi
(1, 3, 2, 6, 4)	2	0.5	16	reduced-foca	{'alpha': 0.5, '	{'block_confi
(1, 3, 2, 6, 4)	2	0.5	16	reduced-foca	{'alpha': 0.5, '	{'block_confi
(1, 3, 2, 6, 4)	2	0.5	8	reduced-foca	{'alpha': 0.5, '	{'block_confi
(1, 3, 2, 6, 4)	2	0.5	8	reduced-foca	{'alpha': 0.5, '	{'block_confi
(2, 6, 4, 12, 8	4	2	16	reduced-foca	{'alpha': 0.25	{'block_confi
(2, 6, 4, 12, 8	4	2	16	reduced-foca	{'alpha': 0.25	{'block_confi
(2, 6, 4, 12, 8	4	2	8	reduced-foca	{'alpha': 0.25	{'block_confi
(2, 6, 4, 12, 8	4	2	8	reduced-foca	{'alpha': 0.25	{'block_confi
(2, 6, 4, 12, 8	2	2	16	reduced-foca	{'alpha': 0.25	{'block_confi
(2, 6, 4, 12, 8	2	2	16	reduced-foca	{'alpha': 0.25	{'block_confi
(2, 6, 4, 12, 8	2	2	8	reduced-foca	{'alpha': 0.25	{'block_confi
(2, 6, 4, 12, 8	2	2	8	reduced-foca	{'alpha': 0.25	{'block_confi
(1, 3, 2, 6, 4)	4	2	16	reduced-foca	{'alpha': 0.25	{'block_confi
(1, 3, 2, 6, 4)	4	2	16	reduced-foca	{'alpha': 0.25	{'block_confi
(1, 3, 2, 6, 4)	4	2	8	reduced-foca	{'alpha': 0.25	{'block_confi

© 2021 Buda M et al. *JAMA Network Open.*

(1, 3, 2, 6, 4)	4	2	8	reduced-foca	{'alpha': 0.25	{'block_confi
(1, 3, 2, 6, 4)	2	2	16	reduced-foca	{'alpha': 0.25	{'block_confi
(1, 3, 2, 6, 4)	2	2	16	reduced-foca	{'alpha': 0.25	{'block_confi
(1, 3, 2, 6, 4)	2	2	8	reduced-foca	{'alpha': 0.25	{'block_confi
(1, 3, 2, 6, 4)	2	2	8	reduced-foca	{'alpha': 0.25	{'block_confi
(2, 6, 4, 12, 8	4	1	16	reduced-foca	{'alpha': 0.25	{'block_confi
(2, 6, 4, 12, 8	4	1	16	reduced-foca	{'alpha': 0.25	{'block_confi
(2, 6, 4, 12, 8	4	1	8	reduced-foca	{'alpha': 0.25	{'block_confi
(2, 6, 4, 12, 8	4	1	8	reduced-foca	{'alpha': 0.25	{'block_confi
(2, 6, 4, 12, 8	2	1	16	reduced-foca	{'alpha': 0.25	{'block_confi
(2, 6, 4, 12, 8	2	1	16	reduced-foca	{'alpha': 0.25	{'block_confi
(2, 6, 4, 12, 8	2	1	8	reduced-foca	{'alpha': 0.25	{'block_confi
(2, 6, 4, 12, 8	2	1	8	reduced-foca	{'alpha': 0.25	{'block_confi
(1, 3, 2, 6, 4)	4	1	16	reduced-foca	{'alpha': 0.25	{'block_confi
(1, 3, 2, 6, 4)	4	1	16	reduced-foca	{'alpha': 0.25	{'block_confi
(1, 3, 2, 6, 4)	4	1	8	reduced-foca	{'alpha': 0.25	{'block_confi
(1, 3, 2, 6, 4)	4	1	8	reduced-foca	{'alpha': 0.25	{'block_confi
(1, 3, 2, 6, 4)	2	1	16	reduced-foca	{'alpha': 0.25	{'block_confi
(1, 3, 2, 6, 4)	2	1	16	reduced-foca	{'alpha': 0.25	{'block_confi
(1, 3, 2, 6, 4)	2	1	8	reduced-foca	{'alpha': 0.25	{'block_confi
(1, 3, 2, 6, 4)	2	1	8	reduced-foca	{'alpha': 0.25	{'block_confi
(2, 6, 4, 12, 8	4	0.5	16	reduced-foca	{'alpha': 0.25	{'block_confi
(2, 6, 4, 12, 8	4	0.5	16	reduced-foca	{'alpha': 0.25	{'block_confi
(2, 6, 4, 12, 8	4	0.5	8	reduced-foca	{'alpha': 0.25	{'block_confi
(2, 6, 4, 12, 8	4	0.5	8	reduced-foca	{'alpha': 0.25	{'block_confi
(2, 6, 4, 12, 8	2	0.5	16	reduced-foca	{'alpha': 0.25	{'block_confi
(2, 6, 4, 12, 8	2	0.5	16	reduced-foca	{'alpha': 0.25	{'block_confi
(2, 6, 4, 12, 8	2	0.5	8	reduced-foca	{'alpha': 0.25	{'block_confi
(2, 6, 4, 12, 8	2	0.5	8	reduced-foca	{'alpha': 0.25	{'block_confi
(1, 3, 2, 6, 4)	4	0.5	16	reduced-foca	{'alpha': 0.25	{'block_confi
(1, 3, 2, 6, 4)	4	0.5	16	reduced-foca	{'alpha': 0.25	{'block_confi
(1, 3, 2, 6, 4)	4	0.5	8	reduced-foca	{'alpha': 0.25	{'block_confi
(1, 3, 2, 6, 4)	4	0.5	8	reduced-foca	{'alpha': 0.25	{'block_confi
(1, 3, 2, 6, 4)	2	0.5	16	reduced-foca	{'alpha': 0.25	{'block_confi
(1, 3, 2, 6, 4)	2	0.5	16	reduced-foca	{'alpha': 0.25	{'block_confi
(1, 3, 2, 6, 4)	2	0.5	8	reduced-foca	{'alpha': 0.25	{'block_confi
(1, 3, 2, 6, 4)	2	0.5	8	reduced-foca	{'alpha': 0.25	{'block_confi
(2, 6, 4, 12, 8	4	2	16	focal	{'alpha': 1.0, '	{'block_confi
(2, 6, 4, 12, 8	4	2	16	focal	{'alpha': 1.0, '	{'block_confi
(2, 6, 4, 12, 8	4	2	8	focal	{'alpha': 1.0, '	{'block_confi
(2, 6, 4, 12, 8	4	2	8	focal	{'alpha': 1.0, '	{'block_confi

© 2021 Buda M et al. *JAMA Network Open.*

(2, 6, 4, 12, 8	2	2	16	focal	{'alpha': 1.0, '{'block_confi
(2, 6, 4, 12, 8	2	2	16	focal	{'alpha': 1.0, '{'block_confi
(2, 6, 4, 12, 8	2	2	8	focal	{'alpha': 1.0, '{'block_confi
(2, 6, 4, 12, 8	2	2	8	focal	{'alpha': 1.0, '{'block_confi
(1, 3, 2, 6, 4)	4	2	16	focal	{'alpha': 1.0, '{'block_confi
(1, 3, 2, 6, 4)	4	2	16	focal	{'alpha': 1.0, '{'block_confi
(1, 3, 2, 6, 4)	4	2	8	focal	{'alpha': 1.0, '{'block_confi
(1, 3, 2, 6, 4)	4	2	8	focal	{'alpha': 1.0, '{'block_confi
(1, 3, 2, 6, 4)	2	2	16	focal	{'alpha': 1.0, '{'block_confi
(1, 3, 2, 6, 4)	2	2	16	focal	{'alpha': 1.0, '{'block_confi
(1, 3, 2, 6, 4)	2	2	8	focal	{'alpha': 1.0, '{'block_confi
(1, 3, 2, 6, 4)	2	2	8	focal	{'alpha': 1.0, '{'block_confi
(2, 6, 4, 12, 8	4	1	16	focal	{'alpha': 1.0, '{'block_confi
(2, 6, 4, 12, 8	4	1	16	focal	{'alpha': 1.0, '{'block_confi
(2, 6, 4, 12, 8	4	1	8	focal	{'alpha': 1.0, '{'block_confi
(2, 6, 4, 12, 8	4	1	8	focal	{'alpha': 1.0, '{'block_confi
(2, 6, 4, 12, 8	2	1	16	focal	{'alpha': 1.0, '{'block_confi
(2, 6, 4, 12, 8	2	1	16	focal	{'alpha': 1.0, '{'block_confi
(2, 6, 4, 12, 8	2	1	8	focal	{'alpha': 1.0, '{'block_confi
(2, 6, 4, 12, 8	2	1	8	focal	{'alpha': 1.0, '{'block_confi
(1, 3, 2, 6, 4)	4	1	16	focal	{'alpha': 1.0, '{'block_confi
(1, 3, 2, 6, 4)	4	1	16	focal	{'alpha': 1.0, '{'block_confi
(1, 3, 2, 6, 4)	4	1	8	focal	{'alpha': 1.0, '{'block_confi
(1, 3, 2, 6, 4)	4	1	8	focal	{'alpha': 1.0, '{'block_confi
(1, 3, 2, 6, 4)	2	1	16	focal	{'alpha': 1.0, '{'block_confi
(1, 3, 2, 6, 4)	2	1	16	focal	{'alpha': 1.0, '{'block_confi
(1, 3, 2, 6, 4)	2	1	8	focal	{'alpha': 1.0, '{'block_confi
(1, 3, 2, 6, 4)	2	1	8	focal	{'alpha': 1.0, '{'block_confi
(2, 6, 4, 12, 8	4	0.5	16	focal	{'alpha': 1.0, '{'block_confi
(2, 6, 4, 12, 8	4	0.5	16	focal	{'alpha': 1.0, '{'block_confi
(2, 6, 4, 12, 8	4	0.5	8	focal	{'alpha': 1.0, '{'block_confi
(2, 6, 4, 12, 8	4	0.5	8	focal	{'alpha': 1.0, '{'block_confi
(2, 6, 4, 12, 8	2	0.5	16	focal	{'alpha': 1.0, '{'block_confi
(2, 6, 4, 12, 8	2	0.5	16	focal	{'alpha': 1.0, '{'block_confi
(2, 6, 4, 12, 8	2	0.5	8	focal	{'alpha': 1.0, '{'block_confi
(2, 6, 4, 12, 8	2	0.5	8	focal	{'alpha': 1.0, '{'block_confi
(1, 3, 2, 6, 4)	4	0.5	16	focal	{'alpha': 1.0, '{'block_confi
(1, 3, 2, 6, 4)	4	0.5	16	focal	{'alpha': 1.0, '{'block_confi
(1, 3, 2, 6, 4)	4	0.5	8	focal	{'alpha': 1.0, '{'block_confi
(1, 3, 2, 6, 4)	4	0.5	8	focal	{'alpha': 1.0, '{'block_confi
(1, 3, 2, 6, 4)	2	0.5	16	focal	{'alpha': 1.0, '{'block_confi

© 2021 Buda M et al. *JAMA Network Open.*

(1, 3, 2, 6, 4)	2	0.5	16	focal	{'alpha': 1.0, '{'block_confi
(1, 3, 2, 6, 4)	2	0.5	8	focal	{'alpha': 1.0, '{'block_confi
(1, 3, 2, 6, 4)	2	0.5	8	focal	{'alpha': 1.0, '{'block_confi
(2, 6, 4, 12, 8	4	2	16	focal	{'alpha': 0.5, '{'block_confi
(2, 6, 4, 12, 8	4	2	16	focal	{'alpha': 0.5, '{'block_confi
(2, 6, 4, 12, 8	4	2	8	focal	{'alpha': 0.5, '{'block_confi
(2, 6, 4, 12, 8	4	2	8	focal	{'alpha': 0.5, '{'block_confi
(2, 6, 4, 12, 8	2	2	16	focal	{'alpha': 0.5, '{'block_confi
(2, 6, 4, 12, 8	2	2	16	focal	{'alpha': 0.5, '{'block_confi
(2, 6, 4, 12, 8	2	2	8	focal	{'alpha': 0.5, '{'block_confi
(2, 6, 4, 12, 8	2	2	8	focal	{'alpha': 0.5, '{'block_confi
(1, 3, 2, 6, 4)	4	2	16	focal	{'alpha': 0.5, '{'block_confi
(1, 3, 2, 6, 4)	4	2	16	focal	{'alpha': 0.5, '{'block_confi
(1, 3, 2, 6, 4)	4	2	8	focal	{'alpha': 0.5, '{'block_confi
(1, 3, 2, 6, 4)	4	2	8	focal	{'alpha': 0.5, '{'block_confi
(1, 3, 2, 6, 4)	2	2	16	focal	{'alpha': 0.5, '{'block_confi
(1, 3, 2, 6, 4)	2	2	16	focal	{'alpha': 0.5, '{'block_confi
(1, 3, 2, 6, 4)	2	2	8	focal	{'alpha': 0.5, '{'block_confi
(1, 3, 2, 6, 4)	2	2	8	focal	{'alpha': 0.5, '{'block_confi
(2, 6, 4, 12, 8	4	1	16	focal	{'alpha': 0.5, '{'block_confi
(2, 6, 4, 12, 8	4	1	16	focal	{'alpha': 0.5, '{'block_confi
(2, 6, 4, 12, 8	4	1	8	focal	{'alpha': 0.5, '{'block_confi
(2, 6, 4, 12, 8	4	1	8	focal	{'alpha': 0.5, '{'block_confi
(2, 6, 4, 12, 8	2	1	16	focal	{'alpha': 0.5, '{'block_confi
(2, 6, 4, 12, 8	2	1	16	focal	{'alpha': 0.5, '{'block_confi
(2, 6, 4, 12, 8	2	1	8	focal	{'alpha': 0.5, '{'block_confi
(2, 6, 4, 12, 8	2	1	8	focal	{'alpha': 0.5, '{'block_confi
(1, 3, 2, 6, 4)	4	1	16	focal	{'alpha': 0.5, '{'block_confi
(1, 3, 2, 6, 4)	4	1	16	focal	{'alpha': 0.5, '{'block_confi
(1, 3, 2, 6, 4)	4	1	8	focal	{'alpha': 0.5, '{'block_confi
(1, 3, 2, 6, 4)	4	1	8	focal	{'alpha': 0.5, '{'block_confi
(1, 3, 2, 6, 4)	2	1	16	focal	{'alpha': 0.5, '{'block_confi
(1, 3, 2, 6, 4)	2	1	16	focal	{'alpha': 0.5, '{'block_confi
(1, 3, 2, 6, 4)	2	1	8	focal	{'alpha': 0.5, '{'block_confi
(1, 3, 2, 6, 4)	2	1	8	focal	{'alpha': 0.5, '{'block_confi
(2, 6, 4, 12, 8	4	0.5	16	focal	{'alpha': 0.5, '{'block_confi
(2, 6, 4, 12, 8	4	0.5	16	focal	{'alpha': 0.5, '{'block_confi
(2, 6, 4, 12, 8	4	0.5	8	focal	{'alpha': 0.5, '{'block_confi
(2, 6, 4, 12, 8	4	0.5	8	focal	{'alpha': 0.5, '{'block_confi
(2, 6, 4, 12, 8	2	0.5	16	focal	{'alpha': 0.5, '{'block_confi
(2, 6, 4, 12, 8	2	0.5	16	focal	{'alpha': 0.5, '{'block_confi

© 2021 Buda M et al. *JAMA Network Open.*

(2, 6, 4, 12, 8	2	0.5	8	focal	{'alpha': 0.5, '{'block_confi
(2, 6, 4, 12, 8	2	0.5	8	focal	{'alpha': 0.5, '{'block_confi
(1, 3, 2, 6, 4)	4	0.5	16	focal	{'alpha': 0.5, '{'block_confi
(1, 3, 2, 6, 4)	4	0.5	16	focal	{'alpha': 0.5, '{'block_confi
(1, 3, 2, 6, 4)	4	0.5	8	focal	{'alpha': 0.5, '{'block_confi
(1, 3, 2, 6, 4)	4	0.5	8	focal	{'alpha': 0.5, '{'block_confi
(1, 3, 2, 6, 4)	2	0.5	16	focal	{'alpha': 0.5, '{'block_confi
(1, 3, 2, 6, 4)	2	0.5	16	focal	{'alpha': 0.5, '{'block_confi
(1, 3, 2, 6, 4)	2	0.5	8	focal	{'alpha': 0.5, '{'block_confi
(1, 3, 2, 6, 4)	2	0.5	8	focal	{'alpha': 0.5, '{'block_confi
(2, 6, 4, 12, 8	4	2	16	focal	{'alpha': 0.25 {'block_confi
(2, 6, 4, 12, 8	4	2	16	focal	{'alpha': 0.25 {'block_confi
(2, 6, 4, 12, 8	4	2	8	focal	{'alpha': 0.25 {'block_confi
(2, 6, 4, 12, 8	4	2	8	focal	{'alpha': 0.25 {'block_confi
(2, 6, 4, 12, 8	2	2	16	focal	{'alpha': 0.25 {'block_confi
(2, 6, 4, 12, 8	2	2	16	focal	{'alpha': 0.25 {'block_confi
(2, 6, 4, 12, 8	2	2	8	focal	{'alpha': 0.25 {'block_confi
(2, 6, 4, 12, 8	2	2	8	focal	{'alpha': 0.25 {'block_confi
(1, 3, 2, 6, 4)	4	2	16	focal	{'alpha': 0.25 {'block_confi
(1, 3, 2, 6, 4)	4	2	16	focal	{'alpha': 0.25 {'block_confi
(1, 3, 2, 6, 4)	4	2	8	focal	{'alpha': 0.25 {'block_confi
(1, 3, 2, 6, 4)	4	2	8	focal	{'alpha': 0.25 {'block_confi
(1, 3, 2, 6, 4)	2	2	16	focal	{'alpha': 0.25 {'block_confi
(1, 3, 2, 6, 4)	2	2	16	focal	{'alpha': 0.25 {'block_confi
(1, 3, 2, 6, 4)	2	2	8	focal	{'alpha': 0.25 {'block_confi
(1, 3, 2, 6, 4)	2	2	8	focal	{'alpha': 0.25 {'block_confi
(2, 6, 4, 12, 8	4	1	16	focal	{'alpha': 0.25 {'block_confi
(2, 6, 4, 12, 8	4	1	16	focal	{'alpha': 0.25 {'block_confi
(2, 6, 4, 12, 8	4	1	8	focal	{'alpha': 0.25 {'block_confi
(2, 6, 4, 12, 8	4	1	8	focal	{'alpha': 0.25 {'block_confi
(2, 6, 4, 12, 8	2	1	16	focal	{'alpha': 0.25 {'block_confi
(2, 6, 4, 12, 8	2	1	16	focal	{'alpha': 0.25 {'block_confi
(2, 6, 4, 12, 8	2	1	8	focal	{'alpha': 0.25 {'block_confi
(2, 6, 4, 12, 8	2	1	8	focal	{'alpha': 0.25 {'block_confi
(1, 3, 2, 6, 4)	4	1	16	focal	{'alpha': 0.25 {'block_confi
(1, 3, 2, 6, 4)	4	1	16	focal	{'alpha': 0.25 {'block_confi
(1, 3, 2, 6, 4)	4	1	8	focal	{'alpha': 0.25 {'block_confi
(1, 3, 2, 6, 4)	4	1	8	focal	{'alpha': 0.25 {'block_confi
(1, 3, 2, 6, 4)	2	1	16	focal	{'alpha': 0.25 {'block_confi
(1, 3, 2, 6, 4)	2	1	16	focal	{'alpha': 0.25 {'block_confi
(1, 3, 2, 6, 4)	2	1	8	focal	{'alpha': 0.25 {'block_confi

© 2021 Buda M et al. *JAMA Network Open.*

(1, 3, 2, 6, 4)	2	1	8	focal	{'alpha': 0.25	{'block_confi
(2, 6, 4, 12, 8	4	0.5	16	focal	{'alpha': 0.25	{'block_confi
(2, 6, 4, 12, 8	4	0.5	16	focal	{'alpha': 0.25	{'block_confi
(2, 6, 4, 12, 8	4	0.5	8	focal	{'alpha': 0.25	{'block_confi
(2, 6, 4, 12, 8	4	0.5	8	focal	{'alpha': 0.25	{'block_confi
(2, 6, 4, 12, 8	2	0.5	16	focal	{'alpha': 0.25	{'block_confi
(2, 6, 4, 12, 8	2	0.5	16	focal	{'alpha': 0.25	{'block_confi
(2, 6, 4, 12, 8	2	0.5	8	focal	{'alpha': 0.25	{'block_confi
(2, 6, 4, 12, 8	2	0.5	8	focal	{'alpha': 0.25	{'block_confi
(1, 3, 2, 6, 4)	4	0.5	16	focal	{'alpha': 0.25	{'block_confi
(1, 3, 2, 6, 4)	4	0.5	16	focal	{'alpha': 0.25	{'block_confi
(1, 3, 2, 6, 4)	4	0.5	8	focal	{'alpha': 0.25	{'block_confi
(1, 3, 2, 6, 4)	4	0.5	8	focal	{'alpha': 0.25	{'block_confi
(1, 3, 2, 6, 4)	2	0.5	16	focal	{'alpha': 0.25	{'block_confi
(1, 3, 2, 6, 4)	2	0.5	16	focal	{'alpha': 0.25	{'block_confi
(1, 3, 2, 6, 4)	2	0.5	8	focal	{'alpha': 0.25	{'block_confi
(1, 3, 2, 6, 4)	2	0.5	8	focal	{'alpha': 0.25	{'block_confi
(2, 6, 4, 12, 8	4		16	weighted-CE	{'alpha': 1.0, '	{'block_confi
(2, 6, 4, 12, 8	4		16	weighted-CE	{'alpha': 1.0, '	{'block_confi
(2, 6, 4, 12, 8	4		8	weighted-CE	{'alpha': 1.0, '	{'block_confi
(2, 6, 4, 12, 8	4		8	weighted-CE	{'alpha': 1.0, '	{'block_confi
(2, 6, 4, 12, 8	2		16	weighted-CE	{'alpha': 1.0, '	{'block_confi
(2, 6, 4, 12, 8	2		16	weighted-CE	{'alpha': 1.0, '	{'block_confi
(2, 6, 4, 12, 8	2		8	weighted-CE	{'alpha': 1.0, '	{'block_confi
(2, 6, 4, 12, 8	2		8	weighted-CE	{'alpha': 1.0, '	{'block_confi
(1, 3, 2, 6, 4)	4		16	weighted-CE	{'alpha': 1.0, '	{'block_confi
(1, 3, 2, 6, 4)	4		16	weighted-CE	{'alpha': 1.0, '	{'block_confi
(1, 3, 2, 6, 4)	4		8	weighted-CE	{'alpha': 1.0, '	{'block_confi
(1, 3, 2, 6, 4)	4		8	weighted-CE	{'alpha': 1.0, '	{'block_confi
(1, 3, 2, 6, 4)	2		16	weighted-CE	{'alpha': 1.0, '	{'block_confi
(1, 3, 2, 6, 4)	2		16	weighted-CE	{'alpha': 1.0, '	{'block_confi
(1, 3, 2, 6, 4)	2		8	weighted-CE	{'alpha': 1.0, '	{'block_confi
(1, 3, 2, 6, 4)	2		8	weighted-CE	{'alpha': 1.0, '	{'block_confi
(2, 6, 4, 12, 8	4		16	CE	{'alpha': 1.0, '	{'block_confi
(2, 6, 4, 12, 8	4		16	CE	{'alpha': 1.0, '	{'block_confi
(2, 6, 4, 12, 8	4		8	CE	{'alpha': 1.0, '	{'block_confi
(2, 6, 4, 12, 8	4		8	CE	{'alpha': 1.0, '	{'block_confi
(2, 6, 4, 12, 8	2		16	CE	{'alpha': 1.0, '	{'block_confi
(2, 6, 4, 12, 8	2		16	CE	{'alpha': 1.0, '	{'block_confi
(2, 6, 4, 12, 8	2		8	CE	{'alpha': 1.0, '	{'block_confi
(2, 6, 4, 12, 8	2		8	CE	{'alpha': 1.0, '	{'block_confi

© 2021 Buda M et al. *JAMA Network Open.*

(1, 3, 2, 6, 4)	4	16	CE	{'alpha': 1.0, '{'block_confi
(1, 3, 2, 6, 4)	4	16	CE	{'alpha': 1.0, '{'block_confi
(1, 3, 2, 6, 4)	4	8	CE	{'alpha': 1.0, '{'block_confi
(1, 3, 2, 6, 4)	4	8	CE	{'alpha': 1.0, '{'block_confi
(1, 3, 2, 6, 4)	2	16	CE	{'alpha': 1.0, '{'block_confi
(1, 3, 2, 6, 4)	2	16	CE	{'alpha': 1.0, '{'block_confi
(1, 3, 2, 6, 4)	2	8	CE	{'alpha': 1.0, '{'block_confi
(1, 3, 2, 6, 4)	2	8	CE	{'alpha': 1.0, '{'block_confi
(2, 6, 4, 12, 8	4	16	weighted-CE	{'alpha': 0.5, '{'block_confi
(2, 6, 4, 12, 8	4	16	weighted-CE	{'alpha': 0.5, '{'block_confi
(2, 6, 4, 12, 8	4	8	weighted-CE	{'alpha': 0.5, '{'block_confi
(2, 6, 4, 12, 8	4	8	weighted-CE	{'alpha': 0.5, '{'block_confi
(2, 6, 4, 12, 8	2	16	weighted-CE	{'alpha': 0.5, '{'block_confi
(2, 6, 4, 12, 8	2	16	weighted-CE	{'alpha': 0.5, '{'block_confi
(2, 6, 4, 12, 8	2	8	weighted-CE	{'alpha': 0.5, '{'block_confi
(2, 6, 4, 12, 8	2	8	weighted-CE	{'alpha': 0.5, '{'block_confi
(1, 3, 2, 6, 4)	4	16	weighted-CE	{'alpha': 0.5, '{'block_confi
(1, 3, 2, 6, 4)	4	16	weighted-CE	{'alpha': 0.5, '{'block_confi
(1, 3, 2, 6, 4)	4	8	weighted-CE	{'alpha': 0.5, '{'block_confi
(1, 3, 2, 6, 4)	4	8	weighted-CE	{'alpha': 0.5, '{'block_confi
(1, 3, 2, 6, 4)	2	16	weighted-CE	{'alpha': 0.5, '{'block_confi
(1, 3, 2, 6, 4)	2	16	weighted-CE	{'alpha': 0.5, '{'block_confi
(1, 3, 2, 6, 4)	2	8	weighted-CE	{'alpha': 0.5, '{'block_confi
(1, 3, 2, 6, 4)	2	8	weighted-CE	{'alpha': 0.5, '{'block_confi
(2, 6, 4, 12, 8	4	16	CE	{'alpha': 0.5, '{'block_confi
(2, 6, 4, 12, 8	4	16	CE	{'alpha': 0.5, '{'block_confi
(2, 6, 4, 12, 8	4	8	CE	{'alpha': 0.5, '{'block_confi
(2, 6, 4, 12, 8	4	8	CE	{'alpha': 0.5, '{'block_confi
(2, 6, 4, 12, 8	2	16	CE	{'alpha': 0.5, '{'block_confi
(2, 6, 4, 12, 8	2	16	CE	{'alpha': 0.5, '{'block_confi
(2, 6, 4, 12, 8	2	8	CE	{'alpha': 0.5, '{'block_confi
(2, 6, 4, 12, 8	2	8	CE	{'alpha': 0.5, '{'block_confi
(1, 3, 2, 6, 4)	4	16	CE	{'alpha': 0.5, '{'block_confi
(1, 3, 2, 6, 4)	4	16	CE	{'alpha': 0.5, '{'block_confi
(1, 3, 2, 6, 4)	4	8	CE	{'alpha': 0.5, '{'block_confi
(1, 3, 2, 6, 4)	4	8	CE	{'alpha': 0.5, '{'block_confi
(1, 3, 2, 6, 4)	2	16	CE	{'alpha': 0.5, '{'block_confi
(1, 3, 2, 6, 4)	2	16	CE	{'alpha': 0.5, '{'block_confi
(1, 3, 2, 6, 4)	2	8	CE	{'alpha': 0.5, '{'block_confi
(1, 3, 2, 6, 4)	2	8	CE	{'alpha': 0.5, '{'block_confi
(2, 6, 4, 12, 8	4	16	weighted-CE	{'alpha': 0.25 {'block_confi

© 2021 Buda M et al. *JAMA Network Open.*

(2, 6, 4, 12, 8	4	16	weighted-CE	{'alpha': 0.25	{'block_confi
(2, 6, 4, 12, 8	4	8	weighted-CE	{'alpha': 0.25	{'block_confi
(2, 6, 4, 12, 8	4	8	weighted-CE	{'alpha': 0.25	{'block_confi
(2, 6, 4, 12, 8	2	16	weighted-CE	{'alpha': 0.25	{'block_confi
(2, 6, 4, 12, 8	2	16	weighted-CE	{'alpha': 0.25	{'block_confi
(2, 6, 4, 12, 8	2	8	weighted-CE	{'alpha': 0.25	{'block_confi
(2, 6, 4, 12, 8	2	8	weighted-CE	{'alpha': 0.25	{'block_confi
(1, 3, 2, 6, 4)	4	16	weighted-CE	{'alpha': 0.25	{'block_confi
(1, 3, 2, 6, 4)	4	16	weighted-CE	{'alpha': 0.25	{'block_confi
(1, 3, 2, 6, 4)	4	8	weighted-CE	{'alpha': 0.25	{'block_confi
(1, 3, 2, 6, 4)	4	8	weighted-CE	{'alpha': 0.25	{'block_confi
(1, 3, 2, 6, 4)	2	16	weighted-CE	{'alpha': 0.25	{'block_confi
(1, 3, 2, 6, 4)	2	16	weighted-CE	{'alpha': 0.25	{'block_confi
(1, 3, 2, 6, 4)	2	8	weighted-CE	{'alpha': 0.25	{'block_confi
(1, 3, 2, 6, 4)	2	8	weighted-CE	{'alpha': 0.25	{'block_confi
(2, 6, 4, 12, 8	4	16	CE	{'alpha': 0.25	{'block_confi
(2, 6, 4, 12, 8	4	16	CE	{'alpha': 0.25	{'block_confi
(2, 6, 4, 12, 8	4	8	CE	{'alpha': 0.25	{'block_confi
(2, 6, 4, 12, 8	4	8	CE	{'alpha': 0.25	{'block_confi
(2, 6, 4, 12, 8	2	16	CE	{'alpha': 0.25	{'block_confi
(2, 6, 4, 12, 8	2	16	CE	{'alpha': 0.25	{'block_confi
(2, 6, 4, 12, 8	2	8	CE	{'alpha': 0.25	{'block_confi
(2, 6, 4, 12, 8	2	8	CE	{'alpha': 0.25	{'block_confi
(1, 3, 2, 6, 4)	4	16	CE	{'alpha': 0.25	{'block_confi
(1, 3, 2, 6, 4)	4	16	CE	{'alpha': 0.25	{'block_confi
(1, 3, 2, 6, 4)	4	8	CE	{'alpha': 0.25	{'block_confi
(1, 3, 2, 6, 4)	4	8	CE	{'alpha': 0.25	{'block_confi
(1, 3, 2, 6, 4)	2	16	CE	{'alpha': 0.25	{'block_confi
(1, 3, 2, 6, 4)	2	16	CE	{'alpha': 0.25	{'block_confi
(1, 3, 2, 6, 4)	2	8	CE	{'alpha': 0.25	{'block_confi
(1, 3, 2, 6, 4)	2	8	CE	{'alpha': 0.25	{'block_confi

num_init_fea	reduce_th	AUC	TPR1	TPR2
16	0.5	1.93448889	0.4	0.58666667
8	0.5	1.62488889	0.36	0.42666667
16	0.5	2.25342222	0.52888889	0.62666667
8	0.5	2.61413333	0.6	0.73333333
16	0.5	1.98364444	0.408	0.52
8	0.5	1.63608889	0.37333333	0.46666667
16	0.5	2.47297778	0.6	0.70666667
8	0.5	2.20195556	0.46666667	0.61333333
16	0.5	2.0656	0.4	0.57333333

© 2021 Buda M et al. *JAMA Network Open.*

8	0.5	2.55653333	0.62666667	0.70666667
16	0.5	2.39591111	0.45333333	0.69333333
8	0.5	2.48133333	0.58666667	0.72
16	0.5	2.59226667	0.57333333	0.69333333
8	0.5	2.65662222	0.57333333	0.70222222
16	0.5	2.48524444	0.52	0.69333333
8	0.5	2.65546667	0.61333333	0.74666667
16	0.5	1.95875556	0.44512821	0.56148148
8	0.5	2.05822222	0.43466667	0.56
16	0.5	2.63342222	0.61333333	0.73333333
8	0.5	2.30364444	0.46666667	0.66666667
16	0.5	2.11875556	0.49333333	0.57333333
8	0.5	1.59866667	0.32	0.44
16	0.5	2.74728889	0.64	0.72
8	0.5	2.60471111	0.60333333	0.70666667
16	0.5	2.78897778	0.65333333	0.77333333
8	0.5	2.72702222	0.64	0.76
16	0.5	2.52826667	0.57333333	0.70666667
8	0.5	2.45653333	0.52	0.69333333
16	0.5	2.66568889	0.58666667	0.74666667
8	0.5	2.49004444	0.49333333	0.70666667
16	0.5	2.46337778	0.58666667	0.68
8	0.5	2.49466667	0.54333333	0.715
16	0.5	1.74417778	0.34666667	0.49333333
8	0.5	2.13813333	0.42060606	0.61333333
16	0.5	2.66177778	0.62333333	0.74666667
8	0.5	2.55315556	0.53666667	0.70666667
16	0.5	1.87786667	0.37777778	0.53333333
8	0.5	2.11262222	0.48	0.61333333
16	0.5	2.61528889	0.6	0.70666667
8	0.5	2.72533333	0.64	0.76
16	0.5	2.58515556	0.58	0.72
8	0.5	2.21715556	0.48	0.58333333
16	0.5	2.65724444	0.61333333	0.76
8	0.5	2.88817778	0.73619048	0.8
16	0.5	2.77831111	0.63666667	0.76
8	0.5	2.17644444	0.48	0.61333333
16	0.5	2.54684444	0.6	0.69333333
8	0.5	2.41173333	0.51	0.68
16	0.5	1.81928889	0.36	0.48571429
8	0.5	2.60266667	0.56	0.74666667

© 2021 Buda M et al. *JAMA Network Open.*

16	0.5	2.6992	0.56	0.74518519
8	0.5	2.62764444	0.58222222	0.72222222
16	0.5	2.30613333	0.50666667	0.65333333
8	0.5	2.31546667	0.505	0.68
16	0.5	2.47262222	0.56	0.69333333
8	0.5	2.59662222	0.63407407	0.72
16	0.5	2.48764444	0.54666667	0.69333333
8	0.5	2.62933333	0.58666667	0.736
16	0.5	2.54222222	0.56	0.72
8	0.5	2.62088889	0.59333333	0.70431373
16	0.5	2.20097778	0.46666667	0.62666667
8	0.5	2.4336	0.57333333	0.69333333
16	0.5	2.61608889	0.55666667	0.74666667
8	0.5	2.59982222	0.58666667	0.70666667
16	0.5	1.99208889	0.46666667	0.52
8	0.5	2.31102222	0.52	0.65333333
16	0.5	2.6952	0.6	0.71777778
8	0.5	2.76275556	0.61333333	0.77333333
16	0.5	1.684	0.33333333	0.46666667
8	0.5	2.02293333	0.44	0.56333333
16	0.5	2.55813333	0.56	0.72
8	0.5	2.43928889	0.57333333	0.69333333
16	0.5	2.3424	0.47555556	0.66666667
8	0.5	2.5696	0.6	0.73333333
16	0.5	2.83306667	0.66666667	0.77960784
8	0.5	2.5312	0.57333333	0.7
16	0.5	1.99964444	0.41333333	0.55466667
8	0.5	2.72693333	0.66	0.73333333
16	0.5	2.48906667	0.55466667	0.70933333
8	0.5	2.64728889	0.64	0.73333333
16	0.5	2.1792	0.46333333	0.64
8	0.5	2.04088889	0.42133333	0.55238095
16	0.5	2.1168	0.42666667	0.61111111
8	0.5	2.26648889	0.44	0.64
16	0.5	1.8232	0.37833333	0.53333333
8	0.5	2.35742222	0.54	0.63333333
16	0.5	2.48524444	0.55555556	0.68
8	0.5	2.68551111	0.61333333	0.75333333
16	0.5	2.40035556	0.56	0.69333333
8	0.5	2.74462222	0.62666667	0.74666667
16	0.5	2.45635556	0.53333333	0.70666667

© 2021 Buda M et al. *JAMA Network Open.*

8	0.5	2.38577778	0.50666667	0.68
16	0.5	0	0	0
8	0.5	2.66266667	0.61833333	0.72
16	0.5	2.53173333	0.61333333	0.70666667
8	0.5	2.45484444	0.52	0.69333333
16	0.5	1.82497778	0.32888889	0.52
8	0.5	1.73146667	0.34444444	0.50666667
16	0.5	2.81031111	0.68444444	0.76
8	0.5	2.78897778	0.66666667	0.73333333
16	0.5	2.09164444	0.48	0.57333333
8	0.5	1.79031111	0.40606061	0.52
16	0.5	2.57511111	0.56	0.73333333
8	0.5	2.54444444	0.54933333	0.72
16	0.5	2.7552	0.64888889	0.78666667
8	0.5	2.68382222	0.63466667	0.74166667
16	0.5	2.38284444	0.52	0.66666667
8	0.5	2.56595556	0.54666667	0.73333333
16	0.5	1.85146667	0.36266667	0.54666667
8	0.5	2.54515556	0.54933333	0.73333333
16	0.5	2.36542222	0.52	0.6562963
8	0.5	2.25617778	0.42222222	0.65333333
16	0.5	1.80764444	0.38666667	0.53333333
8	0.5	1.99057778	0.41333333	0.57333333
16	0.5	2.752	0.62	0.76190476
8	0.5	2.62062222	0.56666667	0.73333333
16	0.5	2.28906667	0.49333333	0.64
8	0.5	1.94151111	0.45333333	0.53333333
16	0.5	2.75093333	0.59619048	0.73333333
8	0.5	2.44711111	0.53333333	0.69777778
16	0.5	2.59235556	0.6	0.70769231
8	0.5	2.19084444	0.49333333	0.62666667
16	0.5	2.43164444	0.53777778	0.68
8	0.5	2.58737778	0.62666667	0.70666667
16	0.5	2.26533333	0.49333333	0.65333333
8	0.5	2.46293333	0.52	0.68
16	0.5	2.58195556	0.57333333	0.705
8	0.5	2.53991111	0.52	0.70666667
16	0.5	2.03262222	0.46666667	0.56
8	0.5	1.79733333	0.38666667	0.52
16	0.5	2.59804444	0.6	0.74666667
8	0.5	2.40186667	0.5362963	0.67733333

© 2021 Buda M et al. *JAMA Network Open.*

16	0.5	1.60835556	0.28	0.46266667
8	0.5	2.22933333	0.47619048	0.65333333
16	0.5	2.64888889	0.61333333	0.70666667
8	0.5	2.65608889	0.58666667	0.76
16	0.5	2.28488889	0.44	0.68
8	0.5	2.45031111	0.52	0.72
16	0.5	2.37902222	0.52	0.66666667
8	0.5	2.51973333	0.58666667	0.72
16	0.5	2.36755556	0.52666667	0.68
8	0.5	2.64133333	0.57333333	0.73333333
16	0.5	2.55671111	0.54666667	0.70666667
8	0.5	2.58942222	0.61333333	0.72
16		2.20853333	0.47589744	0.6
8		1.7448	0.38666667	0.48571429
16		2.47084444	0.52	0.70222222
8		2.43928889	0.53555556	0.68
16		1.89804444	0.39666667	0.544
8		1.80791111	0.38666667	0.50666667
16		2.37822222	0.45333333	0.70666667
8		2.40711111	0.55333333	0.69333333
16		2.50497778	0.54333333	0.66666667
8		2.3632	0.48	0.68
16		2.58613333	0.56571429	0.73393939
8		2.59182222	0.54666667	0.72444444
16		2.68168889	0.60222222	0.74666667
8		2.73955556	0.6	0.74933333
16		2.58435556	0.60444444	0.73333333
8		2.26408889	0.45333333	0.64
16		2.01004444	0.44380952	0.57333333
8		1.63875556	0.34666667	0.44
16		2.37857778	0.4969697	0.68
8		2.39626667	0.50222222	0.66666667
16		1.94764444	0.37904762	0.54666667
8		1.85253333	0.41333333	0.53238095
16		2.59564444	0.61	0.71515152
8		2.51608889	0.58333333	0.72
16		2.668	0.58666667	0.76
8		2.49733333	0.53333333	0.72
16		2.47742222	0.54666667	0.69333333
8		2.5928	0.59111111	0.73333333
16		2.40728889	0.53185185	0.65916667

© 2021 Buda M et al. *JAMA Network Open.*

8	2.50151111	0.57333333	0.68
16	2.54248889	0.52	0.73333333
8	2.67351111	0.6	0.73333333
16	1.9832	0.44	0.56
8	1.89208889	0.38222222	0.53333333
16	2.32035556	0.48666667	0.64
8	2.73955556	0.64	0.73333333
16	1.60471111	0.33333333	0.46666667
8	2.14524444	0.472	0.58666667
16	2.56257778	0.6	0.70666667
8	2.38453333	0.50666667	0.68133333
16	2.80204444	0.67466667	0.76
8	2.62782222	0.58666667	0.73333333
16	0	0	0
8	2.57342222	0.57333333	0.70974359
16	2.77128889	0.65333333	0.76
8	2.48311111	0.545	0.69333333
16	0	0	0
8	2.70382222	0.58666667	0.74666667
16	1.91511111	0.44	0.54666667
8	1.79368889	0.36533333	0.49066667
16	2.78871111	0.62181818	0.77333333
8	2.42684444	0.52	0.69333333
16	1.64293333	0.30358974	0.46666667
8	1.87662222	0.44	0.54666667
16	2.21395556	0.52	0.63703704
8	2.59262222	0.57333333	0.72
16	2.63911111	0.5752381	0.73333333
8	2.31911111	0.45333333	0.66666667
16	2.39431111	0.50564103	0.69333333
8	2.35511111	0.51111111	0.66666667
16	2.56115556	0.53333333	0.72
8	2.77173333	0.66666667	0.76
16	2.50684444	0.54666667	0.69333333
8	2.33626667	0.52	0.66666667
16	2.28915556	0.46666667	0.66666667
8	2.30773333	0.53466667	0.65333333
16	2.57795556	0.58666667	0.72
8	2.68204444	0.62	0.74666667
16	1.83226667	0.37466667	0.49728395
8	1.93848889	0.45333333	0.52

© 2021 Buda M et al. *JAMA Network Open.*

16	2.60124444	0.57333333	0.72
8	2.52471111	0.56	0.7
16	2.5584	0.57833333	0.72
8	2.56871111	0.6	0.70666667
16	2.79804444	0.6	0.78
8	2.83937778	0.66133333	0.77333333
16	2.57973333	0.63333333	0.72
8	2.45324444	0.56	0.69333333
16	2.63937778	0.60888889	0.72727273
8	2.61404444	0.61714286	0.70666667
16	1.94204444	0.42666667	0.51555556
8	1.99173333	0.44	0.5255914
16	2.46826667	0.52	0.7
8	2.60302222	0.6	0.72
16	2.53146667	0.6	0.69333333
8	2.3512	0.54666667	0.64
16	2.67644444	0.61333333	0.73333333
8	2.39724444	0.52	0.69333333
16	2.32231111	0.54333333	0.62666667
8	2.73306667	0.64	0.74666667
16	2.58088889	0.58133333	0.73777778
8	2.49306667	0.54666667	0.70666667
16	2.3472	0.52	0.66666667
8	2.47431111	0.56	0.68
16	2.45511111	0.55333333	0.69333333
8	2.46168889	0.56	0.69333333
16	1.88071111	0.37333333	0.54666667
8	1.67946667	0.37833333	0.46666667
16	2.4992	0.592	0.68
8	2.49608889	0.54666667	0.68833333
16	1.78684444	0.34666667	0.50666667
8	1.99706667	0.464	0.54666667
16	2.27786667	0.49333333	0.64
8	2.33111111	0.48848485	0.66666667
16	2.47048889	0.48888889	0.70666667
8	2.66195556	0.6	0.73333333
16	2.39768889	0.53333333	0.69333333
8	2.63866667	0.62166667	0.69714286
16	2.53795556	0.59333333	0.70666667
8	2.34773333	0.53333333	0.66666667
16	2.29466667	0.52	0.62666667

© 2021 Buda M et al. *JAMA Network Open.*

8	2.0776	0.42	0.62666667
16	1.61377778	0.34666667	0.45333333
8	1.73013333	0.36444444	0.50666667
16	2.60195556	0.58952381	0.72
8	2.45386667	0.54666667	0.664
16	1.96746667	0.44166667	0.57066667
8	2.01404444	0.4	0.57333333
16	2.34302222	0.54666667	0.6762963
8	2.61333333	0.61333333	0.69333333
16	2.74186667	0.64	0.76
8	2.12577778	0.48	0.57333333
16	2.65768889	0.58666667	0.74666667
8	2.37893333	0.56	0.66666667
16	2.58764444	0.58095238	0.70518519
8	2.81946667	0.66666667	0.77333333
16	2.48293333	0.53333333	0.70666667
8	2.61137778	0.57333333	0.72266667
16	1.86773333	0.42666667	0.52
8	2.09075556	0.49333333	0.57333333
16	2.76826667	0.58666667	0.74666667
8	2.33271111	0.52666667	0.62
16	1.78764444	0.4230303	0.50666667
8	1.92666667	0.44	0.54666667
16	2.55208889	0.55333333	0.70666667
8	2.57315556	0.6	0.71428571
16	2.72888889	0.66666667	0.74666667
8	2.57653333	0.6	0.72
16	2.45262222	0.54	0.69333333
8	2.51342222	0.51911111	0.70666667
16	2.16293333	0.44	0.61333333
8	1.94586667	0.42666667	0.58666667
16	2.62275556	0.58666667	0.74666667
8	2.39644444	0.54666667	0.66666667
16	2.26995556	0.48666667	0.64
8	1.94071111	0.42222222	0.53333333
16	2.57466667	0.50666667	0.72
8	2.77733333	0.66666667	0.74666667
16	2.18897778	0.50133333	0.6
8	1.91715556	0.43111111	0.54133333
16	1.724	0.38666667	0.48833333
8	2.74773333	0.64	0.77333333

© 2021 Buda M et al. *JAMA Network Open.*

16	2.50346667	0.59666667	0.70666667
8	2.65973333	0.58666667	0.73333333
16	2.34622222	0.50933333	0.67583333
8	2.38897778	0.48740741	0.68
16	2.61991111	0.56	0.72
8	2.14231111	0.45333333	0.57333333
16	2.4656	0.56	0.70666667
8	2.44995556	0.54666667	0.69333333
16	2.38897778	0.52	0.68
8	1.71475556	0.34666667	0.48
16	2.17866667	0.488	0.59
8	2.68568889	0.64444444	0.70666667
16	2.4152	0.54285714	0.65939394
8	2.55155556	0.56	0.69333333
16	2.47431111	0.54666667	0.68
8	2.58773333	0.64	0.70666667
16	2.32835556	0.53333333	0.66666667
8	2.73191111	0.61444444	0.74666667
16	2.56417778	0.58933333	0.70666667
8	2.72008889	0.62666667	0.73333333
16	2.54853333	0.54666667	0.69212121
8	2.59066667	0.58666667	0.72
16	2.3304	0.46666667	0.68
8	2.536	0.54166667	0.71111111
16	1.72951111	0.30666667	0.49333333
8	1.70231111	0.4	0.45933333
16	2.36764444	0.50666667	0.65333333
8	2.49564444	0.62666667	0.70666667
16	2.39102222	0.576	0.65777778
8	2.19893333	0.45333333	0.64
16	2.67608889	0.65333333	0.75333333
8	2.35617778	0.50666667	0.65333333
16	2.58328889	0.53333333	0.72666667
8	2.32711111	0.47555556	0.67212121
16	2.44088889	0.54666667	0.68
8	2.67582222	0.64952381	0.73333333
16	2.6392	0.57333333	0.74666667
8	2.5616	0.56	0.73333333
16	2.19688889	0.48666667	0.64
8	2.3168	0.49333333	0.6544
16	2.34604444	0.49333333	0.65333333

© 2021 Buda M et al. *JAMA Network Open.*

8	2.32986667	0.51238095	0.62166667
16	2.53644444	0.56	0.72
8	2.59342222	0.5769697	0.71393939
16	2.36053333	0.51333333	0.66666667
8	2.0296	0.43666667	0.57333333
16	2.32568889	0.48	0.66666667
8	2.56017778	0.49333333	0.71619048
16	2.68968889	0.53266667	0.752
8	2.76977778	0.66857143	0.74666667
16	2.54782222	0.6	0.69333333
8	2.53866667	0.54666667	0.70666667
16	2.64391111	0.62133333	0.73333333
8	2.61866667	0.6	0.71111111
16	2.58977778	0.57333333	0.70666667
8	2.57244444	0.54666667	0.73333333
16	1.97475556	0.43238095	0.54666667
8	2.09733333	0.45904762	0.56
16	2.34248889	0.49166667	0.66333333
8	2.37582222	0.53333333	0.68
16	2.23902222	0.47333333	0.61333333
8	2.13146667	0.43151515	0.61333333
16	2.09875556	0.42666667	0.56333333
8	2.37777778	0.56	0.66666667
16	2.22257778	0.40733333	0.64
8	2.69066667	0.59111111	0.76
16	2.41564444	0.50666667	0.67666667
8	2.52017778	0.55111111	0.70666667
16	2.24942222	0.46666667	0.67333333
8	2.29137778	0.50222222	0.66666667
16	2.3848	0.525	0.68848485
8	2.09484444	0.42871795	0.57333333
16	1.92675556	0.37111111	0.54666667
8	1.89573333	0.38666667	0.53333333
16	2.39208889	0.53333333	0.68
8	2.5528	0.61428571	0.72
16	1.93831111	0.40444444	0.54666667
8	2.10026667	0.44	0.58666667
16	2.66142222	0.65333333	0.70666667
8	2.7584	0.61333333	0.74933333
16	2.7288	0.62555556	0.76
8	2.5584	0.58666667	0.70666667

© 2021 Buda M et al. *JAMA Network Open.*

16		2.53635556	0.57333333	0.73333333
8		2.51048889	0.59238095	0.69333333
16		2.55688889	0.57111111	0.70666667
8		2.58817778	0.61333333	0.70666667
16		2.34373333	0.42666667	0.69238095
8		2.53466667	0.54666667	0.72
16	0.5	1.57315556	0.33333333	0.42666667
8	0.5	1.74444444	0.33111111	0.49333333
16	0.5	2.61688889	0.595	0.70666667
8	0.5	2.61875556	0.61333333	0.73333333
16	0.5	2.35253333	0.46666667	0.62666667
8	0.5	2.03911111	0.44	0.55794872
16	0.5	2.5624	0.62666667	0.71733333
8	0.5	2.63911111	0.56	0.73333333
16	0.5	2.58186667	0.58666667	0.70666667
8	0.5	2.55973333	0.56	0.73333333
16	0.5	2.57253333	0.57333333	0.73333333
8	0.5	2.68186667	0.61333333	0.76
16	0.5	2.50577778	0.525	0.70666667
8	0.5	2.66524444	0.62814815	0.73333333
16	0.5	2.65306667	0.61333333	0.72
8	0.5	2.42782222	0.49111111	0.70666667
16	0.5	1.81511111	0.43333333	0.50666667
8	0.5	2.04942222	0.44761905	0.56
16	0.5	2.57137778	0.61666667	0.73333333
8	0.5	2.62844444	0.56	0.755
16	0.5	2.0544	0.45333333	0.56333333
8	0.5	1.47137778	0.28	0.44
16	0.5	2.3416	0.55333333	0.65333333
8	0.5	2.86373333	0.64	0.78666667
16	0.5	2.54737778	0.54666667	0.70666667
8	0.5	1.51697778	0.32444444	0.41333333
16	0.5	2.52853333	0.53866667	0.71733333
8	0.5	2.43848889	0.56	0.68
16	0.5	2.61333333	0.58666667	0.73333333
8	0.5	2.83386667	0.64	0.76
16	0.5	2.48284444	0.56	0.70666667
8	0.5	2.48853333	0.55111111	0.69333333
16	0.5	1.69004444	0.34666667	0.47384615
8	0.5	1.67511111	0.35515152	0.4515942
16	0.5	2.4208	0.56	0.68

© 2021 Buda M et al. *JAMA Network Open.*

8	0.5	2.25733333	0.49777778	0.65333333
16	0.5	1.71555556	0.36095238	0.49333333
8	0.5	2.02942222	0.40952381	0.57166667
16	0.5	2.52924444	0.62666667	0.69166667
8	0.5	1.88426667	0.41333333	0.53333333
16	0.5	2.7416	0.61333333	0.74285714
8	0.5	2.0976	0.42	0.60571429
16	0.5	2.51733333	0.56	0.70666667
8	0.5	2.62195556	0.57333333	0.74666667
16	0.5	2.07662222	0.472	0.6
8	0.5	2.40471111	0.53333333	0.66666667
16	0.5	2.668	0.65333333	0.73333333
8	0.5	2.39075556	0.56952381	0.66666667
16	0.5	1.9056	0.38933333	0.56
8	0.5	1.86293333	0.41333333	0.52
16	0.5	2.47528889	0.53333333	0.69333333
8	0.5	2.69493333	0.6	0.76
16	0.5	2.056	0.43833333	0.58666667
8	0.5	1.9904	0.40888889	0.55111111
16	0.5	2.4632	0.56	0.69555556
8	0.5	2.12506667	0.44	0.62431373
16	0.5	2.57182222	0.59555556	0.72
8	0.5	2.49991111	0.53333333	0.70666667
16	0.5	1.97013333	0.45333333	0.54666667
8	0.5	2.70755556	0.62	0.78666667
16	0.5	2.64088889	0.59	0.72533333
8	0.5	2.50213333	0.54666667	0.68
16	0.5	2.39386667	0.52	0.68
8	0.5	2.37537778	0.49333333	0.70083333
16	0.5	1.69964444	0.36	0.48
8	0.5	1.65093333	0.37333333	0.45333333
16	0.5	2.61271111	0.6	0.72
8	0.5	2.66835556	0.62333333	0.72
16	0.5	2.15022222	0.48	0.6
8	0.5	1.96817778	0.41333333	0.54666667
16	0.5	2.11395556	0.43333333	0.6
8	0.5	2.79848889	0.67272727	0.76
16	0.5	2.68791111	0.61666667	0.73333333
8	0.5	2.63431111	0.58666667	0.74666667
16	0.5	2.53733333	0.61403509	0.69333333
8	0.5	2.81528889	0.63555556	0.77333333

© 2021 Buda M et al. *JAMA Network Open.*

16	0.5	2.45884444	0.51466667	0.66666667
8	0.5	2.47413333	0.51555556	0.72
16	0.5	2.41964444	0.54666667	0.66666667
8	0.5	2.7832	0.65555556	0.77333333
16	0.5	1.76	0.34933333	0.50666667
8	0.5	2.18764444	0.50666667	0.61333333
16	0.5	2.53155556	0.57333333	0.70666667
8	0.5	2.65306667	0.62666667	0.73333333
16	0.5	1.98773333	0.45833333	0.57333333
8	0.5	1.64844444	0.33333333	0.47130435
16	0.5	2.41715556	0.50666667	0.69333333
8	0.5	2.76622222	0.62666667	0.77333333
16	0.5	2.5672	0.58666667	0.70666667
8	0.5	2.63591111	0.58518519	0.70666667
16	0.5	2.60088889	0.62666667	0.70666667
8	0.5	2.59484444	0.62222222	0.685
16	0.5	2.46888889	0.57	0.68
8	0.5	2.52266667	0.53333333	0.69333333
16	0.5	2.22711111	0.43809524	0.66666667
8	0.5	2.67511111	0.59555556	0.73
16	0.5	1.71475556	0.37454545	0.48666667
8	0.5	1.70355556	0.36	0.48
16	0.5	2.65724444	0.63555556	0.74666667
8	0.5	2.74142222	0.58666667	0.74666667
16	0.5	2.10106667	0.45333333	0.57333333
8	0.5	2.13475556	0.50666667	0.56828829
16	0.5	2.74782222	0.65333333	0.74666667
8	0.5	2.55226667	0.49333333	0.73333333
16	0.5	2.4472	0.54666667	0.68
8	0.5	2.46613333	0.54666667	0.68266667
16	0.5	2.31697778	0.49333333	0.66666667
8	0.5	2.40302222	0.49333333	0.66666667
16	0.5	2.51884444	0.57333333	0.67636364
8	0.5	2.55751111	0.54133333	0.73333333
16	0.5	2.55075556	0.59047619	0.72
8	0.5	2.56995556	0.61333333	0.70666667
16	0.5	2.1616	0.49142857	0.61098039
8	0.5	1.74888889	0.36	0.50533333
16	0.5	2.53964444	0.54666667	0.71282051
8	0.5	2.43155556	0.52606061	0.65333333
16	0.5	2.16924444	0.49333333	0.58666667

© 2021 Buda M et al. *JAMA Network Open.*

8	0.5	2.31093333	0.51733333	0.64
16	0.5	2.6056	0.57111111	0.73333333
8	0.5	2.5128	0.57333333	0.70242424
16	0.5	2.46177778	0.54666667	0.66666667
8	0.5	2.6424	0.6	0.74666667
16	0.5	2.82266667	0.62666667	0.766
8	0.5	2.4632	0.54666667	0.69333333
16	0.5	2.6424	0.59272727	0.73333333
8	0.5	2.5384	0.57333333	0.68
16	0.5	2.46568889	0.54666667	0.70666667
8	0.5	2.53546667	0.58666667	0.72
16	0.5	0	0	0
8	0.5	2.56915556	0.54222222	0.73333333
16	0.5	2.59564444	0.55111111	0.76
8	0.5	2.61235556	0.58666667	0.70666667
16	0.5	2.18293333	0.52148148	0.61333333
8	0.5	2.064	0.46333333	0.56
16	0.5	2.57102222	0.58	0.71368421
8	0.5	2.54888889	0.51555556	0.72
16	0.5	2.44026667	0.52	0.73333333
8	0.5	2.38853333	0.51	0.69333333
16	0.5	2.56737778	0.62666667	0.69333333
8	0.5	2.47555556	0.54666667	0.69333333
16	0.5	2.29351111	0.50666667	0.65333333
8	0.5	2.55315556	0.54666667	0.70666667
16	0.5	2.61271111	0.584	0.73333333
8	0.5	2.57475556	0.61333333	0.73333333
16		1.51484444	0.26666667	0.46666667
8		2.33377778	0.47	0.65333333
16		2.40826667	0.49333333	0.70666667
8		2.59546667	0.62444444	0.72444444
16		1.70035556	0.36285714	0.48
8		1.73822222	0.37333333	0.46666667
16		2.48693333	0.53333333	0.70666667
8		2.57831111	0.58666667	0.70666667
16		2.76124444	0.62333333	0.77777778
8		2.50773333	0.57333333	0.68
16		2.77884444	0.56	0.76
8		2.47831111	0.52	0.70782609
16		2.01431111	0.45333333	0.57333333
8		2.35671111	0.52	0.69333333

© 2021 Buda M et al. *JAMA Network Open.*

16	2.42231111	0.56	0.66666667
8	2.49528889	0.53333333	0.72
16	1.93315556	0.36	0.57333333
8	1.64791111	0.29142857	0.46666667
16	2.49048889	0.54666667	0.70666667
8	2.65333333	0.62222222	0.73333333
16	2.00044444	0.46666667	0.57333333
8	1.73866667	0.32	0.48
16	2.49288889	0.53333333	0.72
8	2.58195556	0.62666667	0.72
16	2.66266667	0.57333333	0.74666667
8	2.12044444	0.47407407	0.60733333
16	2.46302222	0.54666667	0.70666667
8	2.75324444	0.62666667	0.76
16	2.61084444	0.58666667	0.73333333
8	2.62408889	0.6	0.73333333
16	2.58533333	0.52666667	0.73333333
8	2.41537778	0.50666667	0.68
16	0	0	0
8	1.93857778	0.44333333	0.56
16	2.70826667	0.65333333	0.73333333
8	2.60088889	0.57333333	0.73333333
16	2.06657778	0.46	0.56
8	1.69742222	0.34933333	0.49333333
16	2.20933333	0.46666667	0.61333333
8	2.67591111	0.66666667	0.73333333
16	2.45333333	0.55384615	0.69333333
8	2.81022222	0.67833333	0.76
16	2.61431111	0.50666667	0.74666667
8	2.50168889	0.51555556	0.72
16	2.69635556	0.64333333	0.74666667
8	2.57137778	0.61333333	0.72
16	2.6568	0.56	0.76
8	2.60302222	0.61904762	0.74933333
16	1.82133333	0.4152381	0.52
8	2.75466667	0.65333333	0.74666667
16	2.38337778	0.50666667	0.68
8	2.54222222	0.53333333	0.73333333
16	1.89386667	0.42666667	0.54666667
8	1.70106667	0.28888889	0.50666667
16	2.52586667	0.57333333	0.70666667

© 2021 Buda M et al. *JAMA Network Open.*

8	2.3296	0.49333333	0.66666667
16	2.46257778	0.46666667	0.72
8	2.40977778	0.50666667	0.68
16	2.46968889	0.56444444	0.69333333
8	2.43635556	0.53333333	0.67111111
16	2.29902222	0.46666667	0.66666667
8	2.66764444	0.58333333	0.73333333
16	2.5144	0.6	0.70666667
8	2.40533333	0.48	0.69333333
16	2.43004444	0.54666667	0.70666667
8	1.56968889	0.31833333	0.45333333
16	2.5584	0.59066667	0.72
8	2.59733333	0.57333333	0.74333333
16	2.14302222	0.47259259	0.58666667
8	2.02693333	0.44666667	0.57333333
16	2.57866667	0.54666667	0.70857143
8	2.38471111	0.57333333	0.68
16	2.38871111	0.49333333	0.69333333
8	2.46897778	0.53904762	0.69333333
16	2.50364444	0.53333333	0.71030303
8	2.33546667	0.53333333	0.65333333
16	2.58257778	0.6	0.73333333
8	2.74364444	0.62666667	0.76
16	2.56782222	0.56380952	0.73333333
8	2.73146667	0.6	0.76
16	1.94977778	0.44	0.5525
8	2.03911111	0.45333333	0.56
16	2.34311111	0.49333333	0.64
8	2.69795556	0.62666667	0.72
16	1.8912	0.41481481	0.52177778
8	2.23475556	0.50666667	0.62877193
16	2.69164444	0.61333333	0.73333333
8	2.63448889	0.58666667	0.74666667
16	2.66631111	0.58666667	0.76
8	2.71004444	0.6	0.74666667
16	2.54328889	0.53333333	0.70666667
8	2.68897778	0.61666667	0.74666667
16	2.70026667	0.615	0.74666667
8	2.16488889	0.45490196	0.61
16	2.68666667	0.61333333	0.73333333
8	2.30906667	0.48	0.66666667

© 2021 Buda M et al. *JAMA Network Open.*

16	1.95253333	0.4	0.56
8	1.94764444	0.45333333	0.52
16	2.6712	0.61866667	0.74666667
8	2.55031111	0.54666667	0.70666667
16	1.91297778	0.43809524	0.54133333
8	1.65155556	0.34666667	0.44
16	2.42835556	0.6	0.65333333
8	2.43155556	0.50666667	0.69333333
16	2.19306667	0.448	0.61333333
8	2.6184	0.61333333	0.73333333
16	2.38791111	0.50666667	0.68
8	2.39662222	0.56444444	0.68
16	2.44328889	0.54666667	0.68848485
8	2.5816	0.56666667	0.70666667
16	2.34062222	0.52	0.68
8	2.5584	0.58909091	0.70666667
16	2.05626667	0.48	0.54333333
8	2.16871111	0.45333333	0.64
16	2.46888889	0.5769697	0.66666667
8	2.6432	0.57666667	0.74666667
16	2.50897778	0.58666667	0.70666667
8	1.87004444	0.36444444	0.54333333
16	2.03937778	0.44777778	0.55
8	2.61084444	0.55555556	0.73333333
16	2.42844444	0.56	0.6647619
8	2.34497778	0.52	0.69333333
16	2.67022222	0.50666667	0.78074074
8	2.44791111	0.54666667	0.69333333
16	2.3696	0.46666667	0.70666667
8	2.58	0.6	0.70666667
16	2.63111111	0.58571429	0.74666667
8	2.4704	0.57333333	0.68
16	1.99937778	0.48	0.56
8	2.15848889	0.48833333	0.6
16	2.51982222	0.54666667	0.70666667
8	2.7072	0.61333333	0.73333333
16	2.47128889	0.52166667	0.70666667
8	2.47946667	0.57333333	0.68
16	2.1064	0.48	0.57333333
8	2.51404444	0.54	0.70666667
16	1.95333333	0.43238095	0.56666667

© 2021 Buda M et al. *JAMA Network Open.*

8	2.53226667	0.53333333	0.70666667
16	2.54133333	0.6	0.70666667
8	2.71048889	0.57333333	0.76
16	2.60124444	0.57333333	0.73333333
8	2.39528889	0.51076923	0.67666667
16	2.41697778	0.57333333	0.68484848
8	2.49146667	0.49333333	0.70666667
16	1.86577778	0.41333333	0.51333333
8	1.99582222	0.4	0.56
16	2.64195556	0.6	0.73333333
8	2.7152	0.61333333	0.73333333
16	1.93573333	0.39333333	0.56
8	1.71431111	0.34333333	0.48848485
16	2.46871111	0.50666667	0.73333333
8	2.75671111	0.68	0.77333333
16	2.34844444	0.53333333	0.66666667
8	2.51031111	0.50666667	0.72
16	2.33502222	0.50666667	0.66285714
8	2.48764444	0.53166667	0.72
16	2.50533333	0.56	0.70666667
8	2.37422222	0.53142857	0.69333333
16	2.55964444	0.55066667	0.72
8	2.64097778	0.58333333	0.73333333
16	2.20853333	0.46666667	0.62666667
8	2.12586667	0.45333333	0.6
16	2.50915556	0.59333333	0.69333333
8	2.51048889	0.59466667	0.68
16	2.09475556	0.45333333	0.57333333
8	2.17146667	0.48	0.61333333
16	2.4056	0.498	0.68
8	2.66231111	0.60533333	0.72
16	2.38924444	0.55666667	0.68
8	2.59297778	0.52	0.72
16	2.67333333	0.60266667	0.73333333
8	2.5672	0.59833333	0.69333333
16	2.73706667	0.655	0.72666667
8	2.65955556	0.59666667	0.74666667
16	2.75048889	0.608	0.77333333
8	2.5288	0.55833333	0.70666667
16	1.90151111	0.39384615	0.56
8	1.91644444	0.38666667	0.53333333

© 2021 Buda M et al. *JAMA Network Open.*

16	2.47795556	0.54666667	0.70666667
8	2.5592	0.56	0.70666667
16	1.87271111	0.408	0.53333333
8	2.03191111	0.465	0.57333333
16	2.57644444	0.55111111	0.76
8	2.57128889	0.56	0.70666667
16	2.42177778	0.56	0.65904762
8	2.27066667	0.53333333	0.622
16	2.34853333	0.56	0.65333333
8	2.62897778	0.61666667	0.73333333
16	2.31662222	0.52	0.66666667
8	2.7328	0.6	0.76
16	2.59111111	0.54962963	0.73333333
8	2.23448889	0.50666667	0.64
16	2.16924444	0.50666667	0.58666667
8	2.39502222	0.52	0.67777778
16	2.47031111	0.52	0.68
8	2.58337778	0.59333333	0.71333333
16	1.78391111	0.38857143	0.49333333
8	2.60408889	0.57333333	0.70833333
16	2.62453333	0.64666667	0.70666667
8	2.67217778	0.58666667	0.74666667
16	2.67573333	0.64	0.74666667
8	2.73324444	0.65333333	0.74666667
16	2.37786667	0.49333333	0.68
8	2.6248	0.62666667	0.74666667
16	2.71075556	0.55555556	0.77066667
8	2.7312	0.64952381	0.74666667
16	2.57511111	0.50666667	0.69333333
8	2.54942222	0.53333333	0.73333333
16	2.14133333	0.52	0.6
8	1.88222222	0.4	0.53333333
16	2.60391111	0.56333333	0.73333333
8	2.77662222	0.6	0.78666667
16	2.30551111	0.52	0.64
8	1.87475556	0.37733333	0.52
16	2.64355556	0.58666667	0.73333333
8	2.45626667	0.54933333	0.70666667
16	2.56168889	0.51809524	0.73333333
8	2.25875556	0.44888889	0.68
16	2.31324444	0.445	0.66666667

© 2021 Buda M et al. *JAMA Network Open.*

8	2.26142222	0.54666667	0.65333333
16	2.35635556	0.46666667	0.69333333
8	2.26035556	0.48	0.64
16	2.15768889	0.46666667	0.61333333
8	2.38755556	0.54666667	0.65333333
16	2.10106667	0.43111111	0.6
8	2.25955556	0.45833333	0.62666667
16	2.87582222	0.68333333	0.78666667
8	2.63955556	0.60888889	0.72
16	1.86675556	0.3875	0.5
8	2.33635556	0.53333333	0.65333333
16	2.62675556	0.56	0.72222222
8	2.42231111	0.52606061	0.66666667
16	2.66471111	0.59246377	0.72
8	2.58764444	0.54666667	0.7047619
16	2.75422222	0.61333333	0.77333333
8	2.40897778	0.51407407	0.68
16	2.64142222	0.65333333	0.73333333
8	2.62408889	0.62666667	0.70666667
16	2.37751111	0.55111111	0.65333333
8	2.45324444	0.50666667	0.69333333

© 2021 Buda M et al. *JAMA Network Open.*
